# Dimension-wise adaptive snake optimizer with Sobol initialization and Lévy spiral search for solar photovoltaic cell model optimization

**DOI:** 10.1038/s41598-026-64941-7

**Published:** 2026-08-11

**Authors:** Manar H. Elgammal, Eman M. El-Gendy, Labib M. Labib, Amira Y. Haikal

**Affiliations:** Computers and Control Systems Engineering Department, Faculty of Engineering, Mansoura University, Mansoura, 35516 Egypt

**Keywords:** Snake optimizer, Dimension-wise, Sobol initialization, Lévy–spiral search operators, PV models., Energy science and technology, Engineering, Mathematics and computing

## Abstract

Metaheuristic algorithms have received significant attention given their ability to handle complex, nonlinear, and high-dimensional optimization problems. Precise parameter identification of photovoltaic (PV) models is crucial for enhancing the performance and reliability of solar energy systems. This study introduces a modification to the Snake Optimizer (SO) algorithm called Dimension-wise Adaptive Snake Optimizer (DASO) with Sobol initialization and Lévy–Spiral search operators to enhance the balance between the exploration and exploitation phases and to strengthen the convergence accuracy of the algorithm. The performance of the proposed approach is tested using a variety of benchmark functions, covering unimodal, multimodal, and composite functions from the CEC test suite. DASO was tested on CEC2017 with various dimensions, and CEC2020. Additionally, non-parametric statistical tests, such as the Wilcoxon signed-rank test for pairwise assessment and the Friedman test for overall comparison, are utilized to confirm the significance of the results. The experimental results indicate that the suggested approach exhibits superior precision, robustness, and convergence capabilities compared to several established metaheuristic techniques. The statistical analysis verifies that the found enhancements are significant at the 5% significance level on the benchmarks. In addition, an ablation study together with exploration–exploitation and population diversity analyses is conducted to validate the contribution of each enhancement mechanism and provide deeper insight into the search behavior of DASO. The proposed method is employed in the estimation of the parameters of the single and double diode photovoltaic cell models. The experimental findings indicate that the proposed method attains lower root mean square error (RMSE) values and enhanced convergence stability in comparison to competing algorithms. The experimental results indicate that the suggested algorithm offers an efficient framework for photovoltaic parameter identification and nonlinear optimization problems.

## Introduction

Metaheuristic algorithms are a strong category of problem-independent optimization methods that have been widely employed to address complex optimization problems defined by nonlinearity, multimodality, discontinuity, and high dimensionality^1^. These challenges commonly occur in engineering design, power systems, signal processing, machine learning, and decision-making applications, where conventional deterministic or gradient-based approaches usually fail to yield satisfying solutions or become computationally impractical. As a result, population-based metaheuristics received significant interest thanks to their flexible nature, absence of derivatives, and powerful global search efficiency^2^.

Population-based metaheuristics function by generating a group of possible solutions through iterative interactions among individuals. By maintaining various search agents, these algorithms are able to concurrently explore different areas of the search space, consequently enhancing robustness against local optima^3^. In the last twenty years, metaheuristics have become a feasible solution for real-world optimization challenges, extensive research efforts have been devoted to developing new methods and improving existing ones to achieve a better balance between exploration and exploitation^4^.

In order to properly explore and exploit s olution spaces, Metaheuristics takes inspiration from social behaviors, biological evolution, and natural events. Because of their adaptability and robust global search capabilities, Metaheuristics have become effective instruments for resolving a wide variety of optimization problems without needing significant problem-specific changes^5^.

Metaheuristic algorithms can be categorized into three main subgroups: Physics-Based Algorithms, Swarm Intelligence Algorithms, and Evolutionary Algorithms. Each category presents unique concepts and techniques that collectively promote the advancement of population-based optimization methods^6^. The first subgroup draw inspiration from fundamental physical laws, utilizing analogies derived from natural processes, while the second subgroup are founded on the collective actions of biological organisms and social beings. The third represents a significant category of stochastic search methodologies derived from the principles of natural evolution and genetic inheritance seen in biological systems^7^.

Over the past decades, a wide range of metaheuristic algorithms have been proposed. Classical population-based approaches include Genetic Algorithms (GA), popular amongst population-based metaheuristics, replicate the mechanisms of natural evolution through selection, crossover, and mutation operators. Genetic Algorithms have been effectively utilized for many discrete and continuous optimization problems. However, they may experience premature convergence and suffer high computing cost when addressing large-scale problems^8^.

Particle Swarm Optimization (PSO) simulates the social behavior of bird flocks and fish schools, in which particles modify their paths according to individual and collective best experiences. Particle Swarm Optimization (PSO) is praised for its rapid convergence and straightforward implementation; nonetheless, its efficiency may decrease in high-dimensional or highly multimodal search spaces because of a rapid decrease in diversity^9^.

Among the different swarm intelligence algorithms, Ant Colony Optimization (ACO) utilizes pheromone-based communication and demonstrates strong efficacy in discrete optimization problems^10^, while Artificial Bee Colony (ABC) mimics the foraging behavior of honeybees through the cooperation of employed, onlooker, and scout bees and has shown competitive performance in multimodal optimization^11^.

The Grey Wolf Optimizer (GWO)^12^ and the Whale Optimization Algorithm (WOA)^13^ are more recent bio-inspired algorithms. They have gained considerable attention thanks to their simplicity and strong exploitation capabilities. They model leadership hierarchy and collective hunting strategies to guide the population toward promising areas in the search space. However, they typically employ uniform update rules across all decision variables, which may limit scalability in high-dimensional optimization problems similar to the performance of GA and PSO.

Regardless of their conceptual diversity, the majority of metaheuristic algorithms exhibit similar limitations when utilized in high-dimensional search spaces. Survey and comparative assessments mostly indicate that premature convergence, low population diversity, and ineffective exploration as the main factors contributing to performance degradation as dimensionality increases. In large-scale problems, different decision variables often exhibit heterogeneous convergence behavior, and uniform search dynamics fail to adapt to these differences^14^. Recent research trends highlight the significance of adaptive control mechanisms, distribution-aware search techniques, and dimension-relevant regulation of the update process to overcome these challenges. Those modifications are intended to enhance convergence stability and scalability without vastly increasing algorithmic complexity^15^.

The Snake Optimizer (SO) has recently emerged as a promising swarm-based metaheuristic inspired by the social behavior of snakes. SO mimics exploration and exploitation through biologically motivated techniques such as searching for food, fighting, and mating behaviors, controlled by food quantity and temperature parameters. Studies have demonstrated that SO achieves competitive performance compared with several established swarm intelligence algorithms^16^. However, like several population-based metaheuristics, the original SO utilizes implicitly uniform movement behavior across all decision variables. In high-dimensional optimization problems, this uniformity can restrict the algorithm’s potential for exploiting heterogeneous convergence patterns, resulting in ineffective refinement in stabilized dimensions or poor exploration in others^17^.

Solar photovoltaic (PV) technology, representing sustainable and renewable energy, has recently attracted significant focus and comprehensive investigation inside the energy sector. The accurate identification of photovoltaic cell performance parameters is critical for optimizing the efficiency of PV systems^18^. It not only enhances system performance and the efficacy of converting energy but also establishes a crucial basis for system monitoring, error diagnostics, and intelligent control^19^. Previous research aimed to tackle several issues in photovoltaic model parameter identification by the implementation of various mathematical models, data fitting algorithms, and machine learning methodologies. Despite this, the complexity of photovoltaic systems, the unpredictability of environmental variations, and the unique characteristics of various cell types are still causing obstacles for the identification of PV parameters^20^.

The parameter identification problem is nonlinear, and the model parameters are strongly coupled, so the standard deterministic methods are often slow or trapped in local optima. Hence, meta-heuristic optimization algorithms are one of the most efficient techniques for PV parameter estimation due to their ability of global searching in the lack of gradient information^21^. Recent research have demonstrated that the accuracy of photovoltaic parameter estimation is not only affected by the optimization technique but also by the mathematical formulation used in the estimate process. The numerical approach used to generate the simulated current is of great importance, strongly influencing the predicted parameters and the resulting root mean square error (RMSE)^22^.

Current research methodologies for determining unknown photovoltaic model parameters are primarily categorized into two types: key point-based methods and current-voltage (I–V) characteristic curve fitting techniques. Key point-based approaches are generally straightforward and easy to implement, yet they frequently lack the precision necessary for accurate parameter extraction^23^. On the other hand, techniques based on I–V characteristic curves aspire to minimize the total error between simulated and measured currents by mathematical optimization techniques^24^.

The first stage in the comprehensive simulation of solar PV systems entails the establishment of a mathematical representation, which is preceded by the determination of essential system parameters. The single-diode (SDM) and double-diode (DDM) configurations are widely utilized in real-world applications. Nonetheless, the contingent aging of the equipment and the undefined parameters predominantly influence the actual performance of the PV models, potentially leading to instability and vulnerability to errors. Consequently, precise estimation of the photovoltaic cell parameters in advance is an essential component of the modeling process^25^.

In this paper, we propose a novel optimizer called Dimension-wise Adaptive Snake Optimizer with Sobol initialization and Lévy–Spiral search operators (DASO), which is an improved variant of SO that increases its overall efficiency by addressing its shortcomings. We have juxtaposed our suggested algorithm against the original SO and some recent algorithms namely Grey Wolf Optimization (GWO)^12^, Sine Cosine Algorithm (SCA)^26^, Harris hawks optimization (HHO)^27^, Moth-flame optimization (MFO)^28^, and the Whale Optimization Algorithm (WOA)^13^. The proposed algorithm is tested on CEC2017 with different dimensions of 10, 30 and 50, and CEC2020. Finally, DASO is used to estimate the parameters of the PV modules, and its performance is compared to SO and other competing algorithms.

This paper primarily contributes in the following practices:

1. Proposing a novel optimization algorithm called DASO for solving engineering problems.
2. Incorporating a Sobol sequence-based initialization strategy to generate a more uniformly distributed initial population, improving search diversity from the early stages of the optimization process.
3. Developing a hybrid search mechanism combining adaptive threshold control, Lévy-flight exploration, and spiral exploitation to achieve a more effective balance between global exploration and local exploitation.
4. Proposing a dimension-wise adaptive step-size strategy to adjust the search intensity of each decision variable independently, improving convergence stability and the algorithm’s performance on high-dimensional optimization problems.
5. Testing the proposed algorithm DASO on CEC2017 with different dimensions of 10, 30 and 50, and CEC2020.
6. Comparing the results of the proposed algorithm with SO and other competing algorithms.
7. Employing the Friedman rank statistical test to evaluate the ranking of the proposed technique in comparison to other algorithms.
8. Applying the Wilcoxon signed-rank test for pairwise statistical comparisons between DASO and other algorithms.
9. Conducting a comprehensive behavioral analysis to investigate the search dynamics of DASO. The analysis includes population diversity and exploration vs. exploitation behavior.
10. Performing an ablation study to quantify the contribution of each enhancement mechanism, demonstrating that the overall performance improvement results from the complementary interaction of the proposed strategies rather than from a single modification.
11. Applying the proposed algorithm DASO to estimate the parameters of PV cells and comparing the results with SO and other state-of-the-art algorithms.

The remainder of this study is structured as follows: Sect. 2 presents a literature review on the original Snake Optimizer (SO), its improved variants, recent state-of-the-art metaheuristic optimization algorithms, learning-assisted optimization approaches, and recent optimization methods for photovoltaic parameter estimation. Section 3 presents the inspiration source and mathematical model of the original SO and the PV cell models. In Sect. 4, the concepts behind our proposed DASO are provided followed by a computational complexity analysis. In Sect. 5, the simulation results of DASO as compared to other algorithms on several benchmark functions are presented and analysed along with a behaviour analysis and an ablation study, followed by a discussion subsection. Section 6 discusses the experimental results of employing DASO for parameter identification of the photovoltaic cell models and analyses its performance in comparison to other competing algorithms. Finally, Sect. 7 provides the conclusion of the study, its limitations, and possible future research.

## Related work

Meta-heuristic optimization algorithms are one of the most successful tools for tackling difficult nonlinear optimization problems because of their flexibility, derivative-free search capacity and robustness in a broad range of application domains^29^. They are capable of effectively searching huge spaces and have been successfully applied to engineering design, renewable energy systems, parameter estimates, scheduling, machine learning, and many other optimization problems^2^.

### Classicalmetaheuristic optimization algorithms

Modern metaheuristic optimization era began with population-based optimization approaches like Genetic Algorithm (GA)^8^ and Particle Swarm optimization (PSO)^9^ that provide collaborative search mechanisms to tackle highly nonlinear optimization problems. Their success has led to the development of a series of nature-inspired algorithms such as Grey Wolf Optimizer (GWO)^12^, Whale Optimization Algorithm (WOA)^13^, Harris Hawks Optimization (HHO)^25^, Moth-Flame Optimization (MFO)^26^, Sine Cosine Algorithm (SCA)^24^, and the recently proposed Snake Optimizer (SO)^16^. These algorithms apply diverse search strategies to explore the candidate solutions towards promising regions while trying to maintain enough diversity in the population.

Although these algorithms have proved competitive results for many engineering applications, they have several typical drawbacks common to them. The primary concerns are premature convergence, loss of diversity in the population, sensitivity to the parameters and a decrease in the effectiveness of the search in the case of high-dimensional optimization problems. Thus, improving convergence stability and keeping a good balance between exploration and exploitation is one of the important study objectives in metaheuristic optimization.

### Recent advances in metaheuristic optimization

Over the past several years, a growing number of studies have focused on adaptive optimization strategies that may change the searching process based on the optimization stage to address the drawbacks of standard optimization algorithms. The most effective are advanced Differential Evolution (DE) variations, such as L-SHADE^30^, LSHADE-cnEpSin, jSO^31^ and NL-SHADE-RSP^32^. These algorithms include adaptive parameter control, lowering of the population size, and enhanced mutation techniques and are consistently ranked among the best in the CEC benchmark contests. Their accomplishment indicates the need of adaptive search techniques to maintain search diversity with improved convergence precision^33^.

Differential Evolution is not the only recent optimization technique; several others have sought inspiration from different mathematics or physics. The Secant Optimization Algorithm (SOA)^34^ uses derivative-free secant approximations to steer the search more efficiently toward potential areas, while keeping the possibility of global exploration. Similarly, the Schrödinger Optimizer (SRA)^35^ draws upon ideas from quantum wave-particle duality to merge probabilistic exploration with adaptive exploitation. These algorithms have been extensively benchmarked and reported in recent papers showing competitive performances on numerical benchmark suites and actual engineering optimization issues. This is a growing trend towards adaptive and mathematically inspired optimization frameworks.

### Machine learning-based optimization algorithms

Recently, machine learning has been used more in combination with metaheuristic optimization algorithms to enhance their search efficiency and flexibility. Different from the metaheuristics, these methods utilize the information of the optimization process to steer the search, adapting the parameters of the algorithm or predicting probable search regions^36^.

Typical examples include reinforcement learning to enable adaptive operator selection, surrogate assisted optimization for objective functions which are computationally expensive, and neural network models for parameter adaptation and search guiding. These learning-based approaches show promising performance for complicated optimization issues regarding convergence speed and solution quality. Machine learning based optimization methods provide benefits, but normally need additional training processes and computer resources, which make implementation more complex^37^.

### Recent hybrid optimization algorithms

Recent studies have put more effort on improving the existing metaheuristic algorithms through the incorporation of adaptive local search and escape mechanisms rather than building new optimization frameworks. The hybrid techniques aim at enhancing the exploration/exploitation balance, the convergence speed and avoiding premature convergence.

This method has been proved to be successful for photovoltaic parameter estimation in several recent research. For instance, a combination of an updated Moth-Flame Optimization method with a Local Escape Operator enhanced the population diversity and resulted in more accurate parameter extraction^38^. Likewise, the accuracy and convergence speed of particle swarm optimization have been further enhanced by employing quadratic interpolation and adaptive local search^39^. These results demonstrate the advantages of integrating complementary search algorithms under a single optimization framework.

Hybrid improvement approaches have also been effectively utilized on large-scale optimization challenges. Recent versions of the Salp Swarm Algorithm have integrated quadratic interpolation and Local Escape Operators to enhance scalability, maintain population diversity, and improve convergence on high-dimensional optimization and feature selection problems^40^.

Likewise, modified Gradient-Based Optimization algorithms that combine quasi-Newton search rules with adaptive local search have shown an enhanced optimization accuracy for photovoltaic parameter extraction. In general, these experiments have demonstrated that combining complimentary enhancement processes is an efficient way to improve the resilience and search ability of metaheuristic algorithms on a wide range of benchmark functions and actual engineering applications^41^.

Motivated by these recent advances, the proposed DASO follows the same direction by enhancing the original Snake Optimizer through multiple complementary adaptive mechanisms. However, DASO combines these strategies within a unified framework to improve population diversity, maintain a better exploration–exploitation balance, and achieve more stable convergence, particularly for complex and high-dimensional optimization problems.

### Variants of the Snake Optimizer SO

SO, by modeling the reproductive and fighting patterns of snakes, achieved competing efficiency thanks to its population division into male and female groups which aims to balance between exploration and exploitation phases^16^. SO is defined by its straightforward structure and little parameter dependency, which improves implementation efficiency and robustness. Despite its simplicity, further studies indicated that the original SO may suffer premature convergence, inadequate population diversity, and poor search efficiency in complex problems, particularly in high-dimensional or highly multimodal optimization problems. Recent research efforts have focused on improving the original SO to improve its convergence stability and global search capability in complex optimization problems^17^.

Yao et al., 2023, proposed an Enhanced Snake Optimizer (ESO) that integrates opposition-based learning and dynamic update mechanisms including sine–cosine composite perturbation factors, Tent-chaos and Cauchy mutation to improve population diversity and reduce premature convergence in real-world engineering optimization problems. Although ESO achieved superior performance across CEC benchmark functions, its multiple enhancement operators increase structural complexity of the algorithms and computational overhead^17^.

Mai et al., 2024, introduced an adaptive Snake Optimizer (ISASO) integrating the Subtraction-Average-Based Optimizer (SABO) to enhance the parameter identification accuracy for photovoltaic cells models. The proposed hybrid method improved the original SO by incorporating a subtraction-average learning operator within the SO structure, using tent chaotic map initialization, a dynamic learning factor and adaptive inertia weight strategy. Their proposed method was tested on CEC2005 benchmark functions and multiple PV cell models, where it outperformed SO and other algorithms in parameter identification accuracy and reliability, achieving the lowest Root Mean Square Error (RMSE). Despite its better precision, its hybrid structure increases computational overhead and introduces cross-algorithm integration complexity^42^.

Liu et al., 2025, proposed an improved adaptive Snake Optimization Evolutionary Algorithm (SOEA) customized for multi-UAV path planning in complex environments. Their approach enhanced SO by incorporating an elite opposition-based learning strategy, an adaptive threshold mechanism. Their method was tested using simulation experiments under dynamic obstacle conditions, and achieved superior path smoothness, shorter travel distance, and faster convergence compared to conventional SO and other metaheuristics. Despite its significantly better performance, the adaptive control mechanisms increased the complexity and required additional parameter tuning, particularly for real-time applications^43^.

Zhu et al., 2025, proposed an Improved Snake Optimizer (ISO), a multi-strategy improved SO incorporating three novel strategies. These strategies are chaotic initialization, a multi-strategy chaotic system (MSCS), an anti-predator strategy (APS), and bidirectional population evolution dynamics (BPED). This method significantly improved the overall performance on CEC-2017 and CEC-2022 benchmarks and various engineering applications. However, ISO depends on the accumulation of multiple enhancement techniques, which may elevate parameter sensitivity and computational complexity^44^.

Zheng et al., 2025, proposed an enhancement to SO called Improved Snake Optimizer (ISO) also applying a multi-strategy technique consisting of Sobol sequence initialization, the RIME algorithm, reverse learning, and Brownian random walk perturbations. This architecture improved population uniformity and exploration capability, but it also introduced extra operators that elevated implementation complexity^45^. Table 1 summarizes some of the variants of the Snake Optimizer (SO).

Despite these enhancements, most SO variants either focus on application-specific effectiveness or introduce additional complexity without consistently balancing exploration and exploitation particularly in high-dimensional or nonlinear parameter estimation problems. This motivates the development of the proposed Dimension-wise Adaptive Snake Optimizer with Sobol initialization and Lévy–Spiral search operators (DASO), which aims to maintain the simplicity of the original SO while strengthening adaptive search behavior, aspiring to accomplish enhanced robustness, better convergence speed and accuracy, and better balance between the exploration and exploitation phases.

**Table 1:** Summary of some variants of the Snake Optimizer (SO).

Author	Methodology	Key advantages	Key limitations
Hashim et al. [16]	Original Snake Optimizer (SO)	Simple structure, few control parameters, balanced exploration–exploitation	May suffer from premature convergence in complex multimodal problems
Yao et al. [17]	Enhanced Snake Optimizer (ESO) with opposition-based learning and dynamic update mechanisms with sine–cosine composite perturbation factors, Tent-chaos and Cauchy mutation	Improved diversity and convergence stability	Increased structural complexity
Mai et al. [42]	Adaptive SO integrating SABO, chaotic initialization and a dynamic learning factor with adaptive inertia weight strategy	Lower RMSE and statistical superiority	Specialized for PV cell modeling
Liu et al. [43][3][3][2][2]	Improved adaptive (SOEA) with elite opposition learning and adaptive threshold	Faster convergence and improved solution accuracy	Application-specific adaptation
Zhu et al. [44]	ISO with multi-strategy enhancement integrating chaotic initialization, (MSCS), (APS), and (BPED)	Superior ranking performance and stability	Higher parameter sensitivity and complexity
Zheng et al. [45]	Improved SO with Sobol initialization, RIME, reverse learning, Brownian random walk perturbation	Enhanced population uniformity and exploration capacity	Multiple integrated operators increase overhead

### Research gap

Although significant progress has been achieved through adaptive metaheuristics, state-of-the-art Differential Evolution variants, learning-assisted optimization frameworks, and hybrid local-search strategies, designing an optimization algorithm that simultaneously maintains population diversity, effectively balances exploration and exploitation, preserves convergence stability, and remains computationally efficient across different optimization landscapes remains a challenging problem. Most existing approaches focus on improving one or two parts of the optimization process, such as adaptive parameter management, local refinement, or escape mechanisms, but very few incorporate many complimentary tactics into a single optimization framework.

These observations motivate this paper to propose the Dimension-wise Adaptive Snake Optimizer (DASO). Rather than introducing a single search operator, DASO combines several mutually complementary mechanisms, including Sobol sequence initialization, adaptive threshold control, Lévy-flight exploration, spiral-based exploitation, and a dimension-wise adaptive step-size mechanism. Together, these strategies improve the quality of the initial population, maintain exploration during the early optimization stages, strengthen local exploitation near convergence, and enhance search stability in high-dimensional optimization problems.

## Background

A background of the basic SO algorithm and PV cell models is provided in this section.

### Snake Optimizer (SO)

The Snake Optimizer (SO), proposed by Hashim et al.^16^, is an optimization algorithm inspired by the mating and foraging behaviours of snakes. By mimicking the fighting and mating behaviors of snake groups, it achieves global optimization. Temperature and food quantity are the two key factors limiting the snakes’ ability to mate. Snakes will only mate when there are enough food and a low temperature. As a result, in SO, the snakes’ primary job is to find food when there is not enough of it, which is referred to as the exploration phase of the optimization algorithm. The snake looks primarily for food in its surroundings in this instance, this emphasizes global search to discover new promising areas in the search space^3,4^. The exploitation phase is more complex and involves multiple sub-phases depending on temperature and food availability. Exploitation focuses on refining existing solutions in promising regions. If food is available and the temperature is high, snakes will concentrate on consuming the available food moving towards the best individuals. If food is available and the temperature is low, the mating process occurs, which can lead to two modes. Fighting mode, in which each male fights to get the best female, and each female tries to select the best male. This involves updating positions based on the best male and female in their respective groups. Mating Mode, on the other hand, takes place between pairs based on food quantity. If mating occurs, there’s a probability that the female lays eggs, leading to new snakes replacing the worst individuals in the population.

Every metaheuristic optimization algorithm requires an initial population. The initial population for the SO is generated by a random uniform distribution with the generation rule described using Eq. (1):

1 $$\:{\mathrm{X}}_{i}={\mathrm{X}}_{min}+{\mathrm{r}}_{i}\times\:\left({\mathrm{X}}_{max}-{\mathrm{X}}_{min}\right),i=\mathrm{1,2},\dots\:,N$$

Where $\:{\mathrm{X}}_{i}$ is the position of the *i-*th individual and $\:{\mathrm{r}}_{i}$ is a randomly generated number $\:\in\:\left[\mathrm{0,1}\right]\:$ and $\:{\mathrm{X}}_{min\:},\:{\mathrm{X}}_{max}\:$ represent the lower and upper bounds of the problem, respectively.

In SO, a simplified gender distribution scenario assuming an equal distribution of male and female snakes is applied, with both genders constituting 50% of the population. The population is then divided into subpopulations of male and female using Eq. (2) and Eq. (3):

2 $$\:{N}_{m}\approx\:\left[\frac{N}{2}\right]$$

3 $$\:{N}_{f}=N-{N}_{m}$$

Where $\:{N,\:N}_{f},{N}_{m}$ are the number of individuals, the number of female individuals, and the number of male individuals, respectively. SO then calculates the best male individual $\:{f}_{{best}_{m}}$, the best female individual $\:{f}_{{best}_{f}}$ and the food location $\:{f}_{food}$ because of its importance as one of the key factors affecting both survival and reproduction.

Temperature and food quantity are the two key factors in SO, as previously stated, that determine snake mating, they are evaluated using Eq. (4) and Eq. (5):

4 $$\:Temp\left(t\right)={e}^{-\frac{t}{T}}$$

5 $$\:Q\left(t\right)={c}_{1}\times\:{e}^{-\frac{t}{T}}$$

Where *t* refers to the current iteration and *T* refers to the maximum number of iterations. $\:{c}_{1}$ is a constant of 0.5.

The exploration phase happens when the food quantity is insufficient. When the food Quantity *Q(t)* is less than a threshold of 0.25, the snakes choose any random location and adjust their position accordingly in order to find food. The exploration phase is applied using Eq. (6) and Eq. (7):

6 $$\:{X}_{i,m}\left(t\:+\:1\right)=\:{X}_{rand,m}\left(t\right)\pm\:\:{c}_{2}\times\:{A}_{m}\:\times\:\:\left(\left({\mathrm{X}}_{max}-{\mathrm{X}}_{min}\right)\times\:rand\:+\:{\mathrm{X}}_{min}\right)$$

7 $$\:{X}_{i,f}\left(t\:+\:1\right)=\:{X}_{rand,f}\left(t\right)\pm\:\:{c}_{2}\times\:{A}_{f}\:\times\:\:\left(\left({\mathrm{X}}_{max}-{\mathrm{X}}_{min}\right)\times\:rand\:+\:{\mathrm{X}}_{min}\right)$$

Where $\:{X}_{i,m}$ and $\:{X}_{i,f}\:$refer to the *i*-th male and female positions respectively, $\:{X}_{rand,m}$ and $\:{X}_{rand,f}$ refer to position of random male and random female respectively, $\:{c}_{2}$ is a constant of 0.05, $\:rand\:$is a random number $\:\in\:\left[\mathrm{0,1}\right].\:\:{A}_{m}$ is the male ability to find food and can be evaluated using Eq. (8), and $\:{A}_{f}$ is the female ability to find food and can be evaluated using Eq. (9):

8 $$\:\:{A}_{m}=\mathrm{exp}\left(\frac{-{f}_{rand,\:m}}{{f}_{i,m}}\right)$$

9 $$\:{A}_{f}=\mathrm{exp}\left(\frac{-{f}_{rand,\:f}}{{f}_{i,f}}\right)$$

Where $\:{f}_{rand,\:m}$ and $\:{f}_{rand,\:f}$ are the fitness values of $\:{X}_{rand,m}$ and $\:{X}_{rand,f}$ respectively, $\:{f}_{i,m}$ is the fitness of *i*-th individual in the male subpopulations and $\:{f}_{i,f}$ is the fitness of *i*-th individual in the female subpopulations.

The exploitation phase occurs when the food quantity is sufficient and the temperature is more than a threshold of 0.6, that indicates that the temperature is hot. In this case, the individuals will concentrate on consuming the available food using Eq. (10):

10 $$\:{X}_{i,j}\left(t\:+\:1\right)=\:{X}_{food}\pm\:\:{c}_{3}\:\times\:Temp\left(t\right)\times\:rand\:\times\:\left({\mathrm{X}}_{food}-{\mathrm{X}}_{i,j}\left(t\right)\right)$$

Where $\:{X}_{i,j}$ is the position of the individual male or the individual female, $\:{\mathrm{X}}_{food}$ is the position of the best individuals, and $\:{c}_{3}$ is a constant of 2.

When the food quantity is sufficient and the temperature is less than the threshold, that indicates that the temperature is cold. In this instance, the snake will go into mating mode. There may be competition between the male and female individuals since both want to complete the mating process with the superior heterosexual. Therefore, there is a fighting mode and a mating mode, the fighting mode is applied using Eq. (11) and Eq. (12):

11 $$\:{X}_{i,m}\left(t\:+\:1\right)=\:{X}_{i,m}\left(t\right)+\:{c}_{3}\times\:{F}_{m}\:\times\:rand\:\times\:\left(Q\left(t\right)\times\:{X}_{best,f}-\:{X}_{i,m}\left(t\right)\right)$$

12 $$\:{X}_{i,f}\left(t\:+\:1\right)=\:{X}_{i,f}\left(t\right)+\:{c}_{3}\times\:{F}_{f}\:\times\:rand\:\times\:\left(Q\left(t\right)\times\:{X}_{best,m}-\:{X}_{i,f}\left(t\right)\right)$$

Where $\:{X}_{best,m}\:$and $\:{X}_{best,f}$ represent the position of the best male and female individual respectively. $\:{F}_{m}$ and $\:{F}_{f}$ refer to the fighting ability of the male and female individuals, respectively. They are evaluated using Eq. (13) and Eq. (14):

13 $$\:{F}_{m}=\mathrm{exp}\left(\frac{-{f}_{best,\:f}}{{f}_{i,m}}\right)$$

14 $$\:{F}_{f}=\mathrm{exp}\left(\frac{-{f}_{best,m}}{{f}_{i,f}}\right)$$

Where $\:{f}_{best,\:m}\:$is the fitness of the best individual in the male subpopulations, and $\:{f}_{best,\:f}$ is the fitness of the best individual in the female subpopulations.

The mating mode is applied using Eq. (15) and Eq. (16):

15 $$\:{X}_{i,m}\left(t\:+\:1\right)=\:{X}_{i,m}\left(t\right)+\:{c}_{3}\times\:{M}_{m}\:\times\:rand\:\times\:\left(Q\left(t\right)\times\:{X}_{i,f}\left(t\right)-\:{X}_{i,m}\left(t\right)\right)$$

16 $$\:{X}_{i,f}\left(t\:+\:1\right)=\:{X}_{i,f}\left(t\right)+\:{c}_{3}\times\:{M}_{f}\:\times\:rand\:\times\:\left(Q\left(t\right)\times\:{X}_{i,m}\left(t\right)-\:{X}_{i,f}\left(t\right)\right)$$

Where $\:{M}_{m}$ and $\:{M}_{f}$ represent the mating ability of the male and female individuals, respectively. They are evaluated using Eq. (17) and Eq. (18):

17 $$\:{M}_{m}=\mathrm{exp}\left(\frac{-{f}_{i,\:f}}{{f}_{i,m}}\right)$$

18 $$\:{M}_{f}=\mathrm{exp}\left(\frac{-{f}_{i,m}}{{f}_{i,f}}\right)$$

After ending the mating process, the female snakes will lay the eggs to obtain new offspring and replace the worst male and female individuals in the original population, depending on the gender of the new snake, respectively. The replacement process is applied using Eq. (19) and Eq. (20):

19 $$\:{\mathrm{X}}_{worst,m}={\mathrm{X}}_{min}+rand\times\:\left({\mathrm{X}}_{max}-{\mathrm{X}}_{min}\right)$$

20 $$\:{\mathrm{X}}_{worst,f}={\mathrm{X}}_{min}+rand\times\:\left({\mathrm{X}}_{max}-{\mathrm{X}}_{min}\right)$$

Where $\:{\mathrm{X}}_{worst,m}\:$is the worst individual in the male subpopulation and $\:{\mathrm{X}}_{worst,f}$ is the worst individual in the female subpopulation. Algorithm 1 shows the pseudo code of the original SO.

**Algorithm 1:**
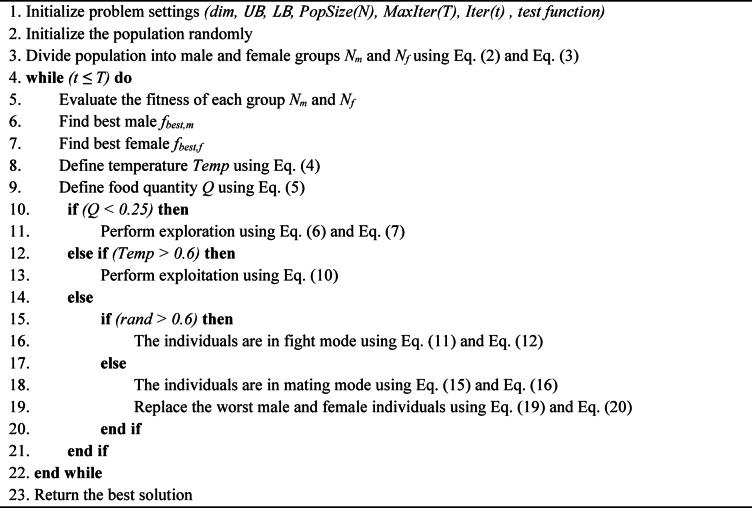
Snake Optimizer Algorithm (SO).

### PV cell

The usage of sources of renewable energy is growing due to several issues linked to climate change and the current energy crisis. Solar energy harvesting is a practical alternative to other clean energy sources, and solar energy systems are currently quickly expanding their market share. Solar power plants are designed with several photovoltaic (PV) cells connected in parallel or serial^46^. PV cells primarily transform absorbed light energy into storable electrical energy via photoelectric or photochemical behavior. The key elements affecting the PV cells’ efficiency are electrical and optical losses^47^. Solar power facilities are integrated into the electrical grid and can operate concurrently with it. Solar photovoltaic plants operate and are sustained at a lower cost than other energy sources. PV generators are complex because to fluctuations in weather^48^.

The regulation of photovoltaic power generation systems relies heavily on the precise and efficient establishment of an equivalent circuit model to represent PV cells, which can ensure optimal power point tracking, enhance performance, and facilitate errors diagnosis^49^. Experimental data and algorithms for optimization can subsequently determine the unidentified parameters^50^.

The voltage, current, and power levels of a solar cell can be evaluated using various methods. These values can be determined numerically using a novel approach that employs the established Newton-Raphson method (NRM). A crucial step in building a model of a PV module is determining the module’s I-V and P-V characteristic curves. PV panels incorporate multiple cells wired in parallel and series arrangements to attain the required current and voltage^51^. The performance of the solar cell is influenced by variations in temperature and solar radiation strength. Numerous models have been produced for the PV cell, including the single-diode model (SDM) and the double-diode model (DDM), which are used in this paper. Of the different mathematical representations of PV modules that have been described in scientific research, the optimum SDM is the most simple one^52^. The model involves three variables: the forward current, reverse current, and diode idealist factor. A lot of work has gone into developing a range of solar cell models that have the potential to accurately forecast performance. Every model has its advantages as well as drawbacks. DDM has an elevated degree of complexity compared with the SDM since SDM requires the identification of five parameters, whereas DDM necessitates seven parameters to be estimated^53^.

The accuracy of the I-V curve computation relies on how many diodes are incorporated in the model. SDMs are frequently employed in the simulation of PV cells or modules because of their significant qualities of reliability, precision, and straightforwardness. This model incorporates the forward current, reverse current, and diode ideality factor as its three variables. However, things are different when there is no series resistance. Researchers have invested significant efforts to establish various solar cell models that offer potential for precise forecasting of performance in real life. Every model has strengths and weaknesses. Previous studies have shown that the DDM is more complicated in structure than the SDM^54,55^.

#### Single-diode model (SDM)

**Fig. 1:**
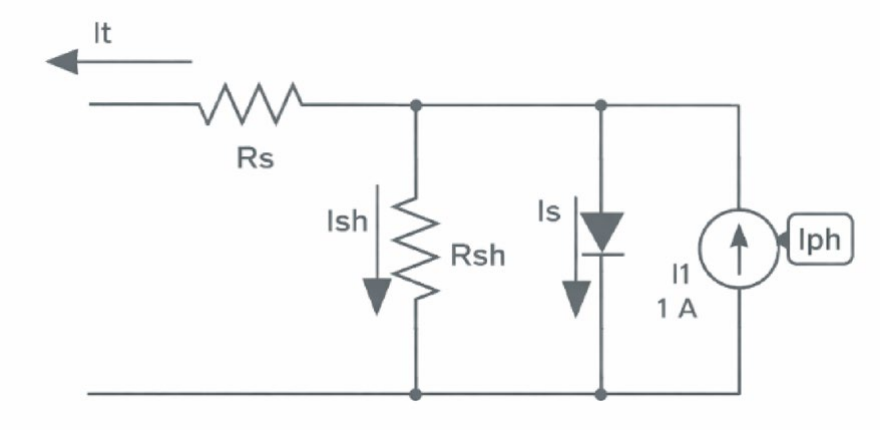
Single-Diode model (SDM).

The SDM structure is straightforward and widely used in real engineering applications. Figure 1 illustrates the corresponding circuit layout, which consists mainly of series and parallel resistors, photovoltaic diode D, and a current source. The mathematics expression for the output current of the SDM model is described using Eq. (21)^56^:

21 $$\:\begin{array}{cccc}&\:{I}_{t}={I}_{ph}-{I}_{sd}\left\{\mathrm{e}\mathrm{x}\mathrm{p}\left[\frac{q\left({V}_{t}+{R}_{s}{I}_{t}\right)}{nkT}\right]-1\right\}-\frac{{V}_{t}+{R}_{s}{I}_{t}}{{R}_{sh}}&\:&\:\end{array}$$

Where $I_{t}$ and $V_{t}$ represent the output current and voltage of the PV cell, respectively. $I_{{ph}}$ is the photocurrent, while $\:{I}_{sd}$ is the reverse saturation current of the PV cell diode. * q* denotes the elementary charge $\:\left( {1.60217646 \times 10^{{ - 19}} \:C} \right)$, * n* is the ideality factor of the diode, * k* is the Boltzmann constant $\:\left( {1.3806503 \times 10^{{ - 23}} \:J/K} \right)$, and * T* is the absolute temperature during the operation of the photovoltaic cell. $\:{R}_{s}$ and $\:{R}_{sh}\:$are the series and shunt resistances, respectively.

The SDM model involves five parameters to be identified: $\:{I}_{ph\:}{,\:I}_{sd},\:n,\:{R}_{s},\:{R}_{sh}$. The minimum and maximum values of these parameters are given in Table 2.

**Table 2:** Lower and Upper bounds for the parameters of the Single-diode model.

Parameter	Lb	Ub
$\:{\boldsymbol{I}}_{\boldsymbol{p}\boldsymbol{h}}$	0	1
$\:{\boldsymbol{I}}_{\boldsymbol{s}\boldsymbol{d}}$	0	1
$\:{\boldsymbol{R}}_{\boldsymbol{s}\boldsymbol{h}\:}$	0	100
$\:{\boldsymbol{R}}_{\boldsymbol{s}\:}$	0	0.5
$\:\boldsymbol{n}$	1	2

#### Double-diode model (SDM)

**Fig. 2:**
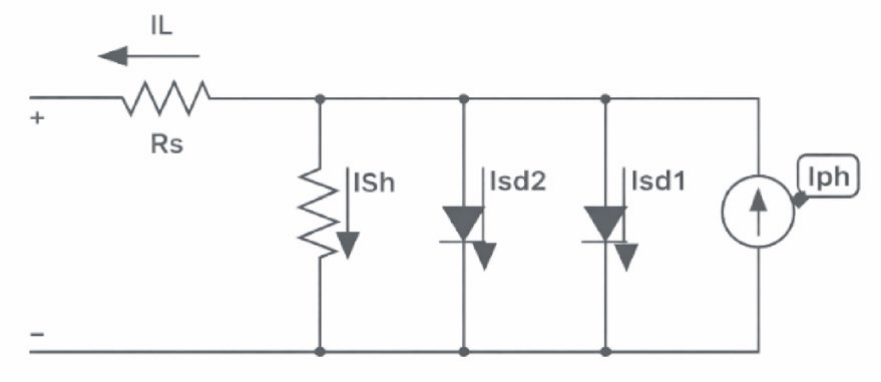
Double Diode model.

The complexity of the DDM is higher than that of the SDM, considering the effects of compound current losses. It offers a more precise assessment of the module’s performance in low-irradiance situations compared to the SDM. Figure 2 illustrates the associated circuit diagram of the DDM. The DDM consists simply of a pair of double diodes, a current generator, and two resistors, where the two parallel function as rectifying devices to emulate compound currents and specific non-ideal situations, allowing for a far more accurate evaluation of PV cell parameters. Furthermore, the DDM entails an expanded list of parameters for identification: $\:{I}_{ph\:}{,\:I}_{sd1}{,\:I}_{sd2},{R}_{s},\:{R}_{sh},\:{n}_{1},\:{n}_{2}$. The upper and lower limits of these parameters are listed in Table 3. The formula for the current output of DDM is as presented in Eq. (22)^57^:

22 $$\:\begin{array}{cccc}&\:{I}_{L}={I}_{ph}-{I}_{sd1}\left\{\mathrm{e}\mathrm{x}\mathrm{p}\left[\frac{q\left({V}_{L}+{R}_{s}{I}_{L}\right)}{{n}_{1}kT}\right]-1\right\}-{I}_{sd2}\left\{\mathrm{e}\mathrm{x}\mathrm{p}\left[\frac{q\left({V}_{L}+{R}_{s}{I}_{L}\right)}{{n}_{2}kT}\right]-1\right\}-\frac{{V}_{L}+{R}_{s}{I}_{L}}{{R}_{sh}}&\:&\:\end{array}$$

Where $\:{I}_{sd1}$ and $\:{I}_{sd2}$ represent the currents of $\:{D}_{1}$ and $\:{D}_{2}$, respectively, while $\:{n}_{1}$ and $\:{n}_{2}$ represent the ideality factors of $\:{D}_{1}$ and $\:{D}_{2}$.

**Table 3:** Lower and Upper bounds for the parameters of the Double-diode model.

Parameter	Lb	Ub
$\:{\boldsymbol{I}}_{\boldsymbol{p}\boldsymbol{h}}$	0	1
$\:{\boldsymbol{I}}_{\boldsymbol{s}\boldsymbol{d}1}$	0	1
$\:{\boldsymbol{I}}_{\boldsymbol{s}\boldsymbol{d}2}$	0	1
$\:{\boldsymbol{R}}_{\boldsymbol{s}\boldsymbol{h}\:}$	0	100
$\:{\boldsymbol{R}}_{\boldsymbol{s}\:}$	0	0.5
$\:{\boldsymbol{n}}_{1}$	1	2
$\:{\boldsymbol{n}}_{2}$	1	2

## Proposed DASO

Although the SO algorithm has accomplished good results in optimization problems, it faces some challenges such as slow convergence rate in the exploration phase which occurs due to the uneven population initialization, poor solution precision, the increased potential of getting trapped in local optima, and that it may stagnate in high dimensions. In order to overcome these challenges, we have added some modifications to the algorithm to enhance its overall performance focusing on enhancing the performance in higher dimensions, the convergence speed, optimization precision, and effectively avoiding falling into local optima. This section describes the DASO algorithm with the four enhancement strategies used based on the weaknesses of SO.

### Initialization using Sobol sequences

 The initial population of SO is generated by a random uniform distribution which may result in the initial snakes being located far from the optimal point, leading to inadequate optimization ability of the algorithm^58^. Sobol sequences are a kind of low-discrepancy sequence that is widely employed in optimization problems and numerical simulations. In order to ensure that the search space is thoroughly searched, especially in high-dimensional issues, these sequences are made to generate points that are uniformly scattered throughout a multidimensional space^59^. This technique guarantees that the starting population is evenly distributed throughout the search space, laying a foundation for the optimization algorithm’s exploration and exploitation stages. Sobol sequences enhance the algorithm’s capability to explore global optima, lower the likelihood of early convergence to suboptimal solutions, and enable a more comprehensive exploration of the problem landscape, which is why it is very beneficial for high-dimensional optimization problems^60^. Each *snake* is initialized using Eq. (23):

23 $$\:{X}_{i}={X}_{min}+{S}_{i}\cdot\:\left({X}_{max}-{X}_{min}\right)$$

Where $\:{S}_{i}\in\:[\mathrm{0,1}{]}^{d}$ represents a low-discrepancy sequence value, each element $\:{S}_{i}$ is a * d*-dimensional vector described by Eq. (24):

24 $$\:{S}_{i}=\left({s}_{i,1},{s}_{i,2},\dots\:,{s}_{i,d}\right)$$

While Sobol initialization enhances the initial population diversity, adaptive management of the search behavior is necessary to preserve this diversity during the optimization process. An adjustable threshold mechanism is subsequently proposed.

### Dynamic parameter updates

In the SO algorithm, the quantity of food is crucial in determining whether the algorithm is in the stage of exploration or exploitation. In Eq. (4), the parameter $\:{c}_{1}$ is defined as a constant value of 0.5, which prevents achieving balance between exploration and exploitation phases, resulting in poor optimization efficiency in the algorithm’s latter stages. At the early iterations, the population lacks knowledge, so exploration should be the main focus, and later, the population has gathered additional data (better individuals) so exploitation should be the main goal. Accordingly, a time-increasing $\:{c}_{1}\left(t\right)\:$is a model of growing confidence, which is more realistic than presuming fixed certainty from the start. To resolve this issue, this study presents a non-linear adaptive coefficient $\:{c}_{1}$ to modify the search effectiveness. The new adaptive $\:{c}_{1}$ is evaluated using Eq. (25):

25 $$\:{c}_{1}\left(t\right)=0.5+{\left(\frac{t}{T}\right)}^{3}$$

An adaptive $\:{c}_{1}\left(t\right)$ reduces sensitivity because it makes $\:Q\left(t\right)$ evolve in a predictable and a bounded way where $\:{c}_{1}\left(t\right)\in\:\left[\mathrm{0.5,1.5}\right]$. Adaptive coefficients dynamically balance between exploration and exploitation phases as iterations go, rather than relying on constant thresholds. An adaptive sigmoid-based threshold is implemented to modify the algorithm’s iteration precision by dynamically regulating the switch between exploration and exploitation phases. Instead of a fixed threshold of 0.25 that controls the exploration phase, *TH1* is evaluated using Eq. (26). TH2 is implemented using Eq. (27) and is used to control the exploitation and mating phase of the methodology instead of a constant value of 0.6.

26 $$\:TH1\left( t \right) = \frac{{0.25}}{{1 + e^{{b\left( {\frac{t}{T} - 0.5} \right)}} }}$$

27 $$\:TH2\left( t \right) = \frac{{0.6}}{{1 + e^{{b\left( {\frac{t}{T} - 0.5} \right)}} }}$$

The adaptive threshold regulates the balance between exploration and exploitation according to the current optimization stage. Larger threshold values during the early iterations encourage global exploration by allowing individuals to investigate diverse regions of the search space. As optimization progresses, the threshold decreases gradually, increasing the probability of exploitation and enabling more accurate refinement around high-quality solutions. Unlike a fixed threshold, the adaptive mechanism provides a smoother and more flexible transition between the two search phases.

While the adaptive threshold regulates the balance between exploration and exploitation, the exploration stage itself is further strengthened through a Lévy-flight search mechanism, and the exploitation capability is enhanced through a spiral search strategy.

### Hybridization strategy

To increase the convergence precision and robustness of the original Snake Optimizer (SO), a hybrid strategy is proposed which combines enhanced exploration using the Lévy flight step with refined exploitation employing spiral motion inspired by the WOA^13^. The proposed hybrid strategy is implemented to resolve the limitations of premature convergence and weak local refinement.

#### Lévy flight-based exploration enhancement

While the original SO offers stochastic exploration, its search steps are primarily constrained and may struggle to escape deep local optima. Lévy flight is utilized to enhance global exploration, thanks to its heavy-tailed step distribution that allows occasional long jumps in the search space^61^. The Lévy flight step is added to the male and female individuals in the exploration phase only if a random ratio is less than a control parameter $\:R\left(t\right)$, which is evaluated using Eq. (28):

28 $$\:R\left(t\right)=1-\frac{t}{T}$$

This parameter decreases linearly from 1 to 0 as the iterations progress. The Lévy distribution parameter $\:\beta\:$ determines the heaviness of the distribution tail and therefore controls the probability of generating long-distance search steps. Following common practice in the metaheuristic optimization literature, $\:\beta\:=1.5$ was adopted to achieve a suitable balance between local search and occasional long jumps. This configuration improves the ability of DASO to escape local optima while maintaining stable convergence behavior. First, the scale parameter $\:\sigma\:$ is evaluated using Eq. (29):

29 $$\:\sigma\:={\left(\frac{\varGamma\:\left(1+\beta\:\right){\hspace{0.17em}}\mathrm{sin}\left(\frac{\pi\:\beta\:}{2}\right)}{\varGamma\:\left(\frac{1+\beta\:}{2}\right){\hspace{0.17em}}\beta\:{\hspace{0.17em}}{2}^{\frac{\beta\:-1}{2}}}\right)}^{\frac{1}{\beta\:}}$$

Where $\:{\Gamma\:}\left(\cdot\:\right)$ indicates the Gamma function. Then, two independent Gaussian random vectors are evaluated using Eq. (30) and the Lévy step is calculated using Eq. (31):

30 $$\:u\sim\:\mathcal{N}\left(0,{\sigma\:}^{2}I\right),\:\:\:\:\:\:\:\:\:\:\:v\sim\:\mathcal{N}\left(0,I\right)$$

31 $$\:step=\frac{u}{\mid\:v{\mid\:}^{\frac{1}{\beta\:}}}$$

Where * I* denotes the identity matrix of size $\:d\times\:d$, indicating that the Gaussian random vectors are independently and identically distributed with zero mean and unit variance across all dimensions. To facilitate a seamless transition from exploration to exploitation, the Lévy step amplitude is scaled by a linearly decaying factor, so that large exploration moves are encouraged early, while the search becomes more conservative near the end. The scaling factor $\:\lambda\:\left(t\right)\:$is defined using Eq. (32):

32 $$\:\lambda \:\left( t \right) = \alpha \: \cdot \:\left( {1 - \frac{t}{T}} \right)$$

Where the scaling coefficient α controls the maximum search step during the exploration stage. A relatively small value (0.05) was selected based on preliminary experiments to maintain sufficient exploration without introducing excessive oscillations. The step-size vector is then defined for the * i*-th male and female individual using Eq. (33) and Eq. (34):

33 $$\:\Delta \:X_{{i,m}} \left( t \right) = \lambda \:\left( t \right)\:.step \odot \:\left( {X_{{i,m}} \left( t \right) - X_{{best,m}} \left( t \right)} \right)$$

34 $$\Delta X_{{i,f}} \left( t \right) = \lambda \:\left( t \right)\:.step \odot \:\left( {X_{{i,f}} \left( t \right) - X_{{best,f}} \left( t \right)} \right)$$

Where $\:\odot\:$ indicates element-wise multiplication. Finally, the new position is updated by applying an additional Gaussian perturbation as described in Eq. (35) and Eq. (36) only when *rand* < $\:R\left(t\right)$:

35 $$\:X_{{i,m}} \left( {t\: + \:1} \right) = \:X_{{i,m}} \left( t \right) + \:\Delta X_{{i,m}} \left( t \right) \odot \: \in ,\:\:\:\:\:\: \in \sim \:{\mathcal{N}}\left( {0,I} \right)$$

36 $$\:X_{{i,f}} \left( {t\: + \:1} \right) = \:X_{{i,f}} \left( t \right) + \:\:\Delta X_{{i,f}} \left( t \right) \odot \in \:\:\:\:\:\:\: \in \sim \:{\mathcal{N}}\left( {0,I} \right)$$

Multiplying by $\:\left( {X_{i} \left( t \right) - X_{{best}} \left( t \right)} \right)$ gives bias to the perturbation concerning the current optimal region, while the additional Gaussian term enhances stochasticity and lowers the probability of deterministic oscillations. This strategy greatly enhances the optimizer’s capacity to investigate uncharted areas and avoid premature convergence.

The proposed search mechanism combines adaptive step-size scaling with Gaussian perturbation to improve the exploration capability of the original Snake Optimizer while maintaining convergence stability. The scaling factor λ(t) decreases linearly throughout the optimization process, allowing larger search steps during the early iterations to encourage global exploration and progressively smaller steps during the later stages to facilitate local refinement around promising regions. This strategy enables DASO to perform broad exploration during the early optimization stages and gradually transition toward accurate local exploitation as convergence progresses, which contributes to improved convergence stability and reduces the likelihood of premature convergence, particularly on multimodal and hybrid optimization problems.

#### Spiral exploitation mechanism

A spiral motion inspired by the WOA^13^ is utilized to improve exploitation accuracy near promising individuals. The original SO exploitation relies on linear attraction, which may cause oscillations. In contrast to linear attraction methods, spiral motion facilitates a smooth and consistent convergence towards the optimal solution. The spiral update rule is applied using Eq. (37) and Eq. (38) only when *rand* > $\:R\left(t\right)$:

37 $$\:{X}_{i,m}\left(t\:+\:1\right)=\mid\:{X}_{best,m}\left(t\right)-{X}_{i,m}\left(t\right)\mid\:\:\cdot\:{e}^{bl}\cdot\:\mathrm{cos}\left(2\pi\:l\right)+{X}_{best,m}\left(t\right)\:$$

38 $$\:{X}_{i,f}\left(t\:+\:1\right)=\mid\:{X}_{best,f}\left(t\right)-{X}_{i,f}\left(t\right)\mid\:\:\cdot\:{e}^{bl}\cdot\:\mathrm{cos}\left(2\pi\:l\right)+{X}_{best,f}\left(t\right)\:$$

The spiral coefficient * b* controls the curvature and intensity of the local search trajectory around promising candidate solutions. Appropriate values enable gradual refinement toward high-quality regions while preventing excessively aggressive movements that could disturb the convergence process. * b* is set to a value of 1 and $\:l\in\:\left[-\mathrm{1,1}\right]$ is a random number. This strategy improves local search precision and enhances convergence stability during the exploitation phase which leads to an improved final solution accuracy.

#### Unified hybrid update rule (summary)

By combining Lévy flight exploration and spiral exploitation under adaptive control and generating a random number $\:\rho\:\sim\:U\left(\mathrm{0,1}\right)$, the overall strategy for updating both the male and female individuals’ positions can be summarized in Eq. (39) as follows:

39 $$\:X_{i} \left( {t\: + \:1} \right) = \left\{ {\begin{array}{*{20}c} {X_{i} \left( t \right) + \:\:\Delta \:X_{i} \left( t \right) \odot \:?} & {\:if\:\exp loration\:is\:selected\:and\:\rho \: < R\left( t \right)} \\ {\:{\mid }\:X_{{best}} \left( t \right) - X_{i} \left( t \right){\mid }\:\: \cdot \:e^{{bl}} \cdot \:\cos \left( {2\pi \:l} \right) + X_{{best}} \left( t \right)} & {\:if\:\exp loitation\:is\:selected\:and\:\rho \:> R\left( t \right)} \\ \end{array} } \right.\:$$

This hybrid strategy delivers a simple yet strong probabilistic framework while preserving the biological inspiration of the original SO, which results in balancing global exploration with local exploitation, facilitating smooth behavioral transition and enhancing convergence performance. Finally, a dimension-wise adaptive step-size mechanism is incorporated.

### Dimension-wise adaptive step size (DASS)

In the original SO, the extent of position updates is uniformly applied to all decision variables. In high-dimensional search spaces, different dimensions frequently display heterogeneous convergence patterns. Utilizing a uniform step size across all dimensions may lead to ineffective exploration, premature convergence, or oscillatory behavior in dimensions that have already stabilized. To address this limitation, a Dimension-Wise Adaptive Step Size (DASS) mechanism is applied at the end of each iteration. This update aims to enhance convergence stability and tailor the search behavior to the characteristics of individual decision variables, while maintaining the original framework of SO.

At each iteration, the variance of the population along each decision variable is computed to quantify the degree of dispersion. A larger variance indicates that the corresponding dimension remains under-explored, whereas a smaller variance suggests that the population has converged in that dimension. Based on this information, an adaptive scaling factor is derived for each dimension. This variance-guided adaptation suppresses unnecessary oscillations in converged dimensions while maintaining effective search pressure in less explored ones. Therefore, the algorithm provides a better balance between exploration and exploitation, specifically for large scale optimization problems where using a uniform step size may negatively impact the performance. To compute the variance for each dimension, we combine all male and female individuals into one population. Concatenating them gives the full population matrix of size $\:N\times\:D$ as described in Eq. (40) where *D* is the number of dimensions:

40 $$\:\mathrm{X}\left(t\right)=\left[\begin{array}{c}{\mathrm{X}}_{m}\left(t\right)\\\:{\mathrm{X}}_{f}\left(t\right)\end{array}\right]$$

Then we evaluate variance for each dimension using Eq. (41) and the dimension-wise adaptive step-size is calculated using Eq. (42):

41 $$\:{\sigma\:}_{d}^{2}\left(t\right)=\mathrm{V}\mathrm{a}\mathrm{r}\left({X}_{1,d}\left(t\right),{X}_{2,d}\left(t\right),\dots\:,{X}_{N,d}\left(t\right)\right),\:\:\:\:\:\:\:\:\:\:\:\:d=1,\dots\:,D$$

42 $$\:{\eta\:}_{d}\left(t\right)=\frac{1}{1+{\sigma\:}_{d}^{2}\left(t\right)}$$

The population variance is used to estimate how well each decision variable has converged during the optimization process. When the variance of a particular dimension is high, the candidate solutions remain widely scattered, indicating that the search in that dimension is still active and additional exploration is needed. In this situation, the adaptive coefficient is reduced to moderate the search step, preventing unstable movements while maintaining sufficient diversity for further exploration. On the other hand, a low variance indicates that the population has largely converged along that dimension. The adaptive coefficient is therefore increased, allowing the search to focus more strongly on refining the current best solution and improving local search accuracy.

The adaptive coefficient $\:{\eta\:}_{d}$ is determined directly from the population variance of each decision variable. Consequently, every dimension receives an independent search intensity according to its convergence state.

Unlike conventional adaptive step-size methods that apply the same adjustment to all decision variables, the proposed strategy adapts the search step independently for each dimension according to its convergence state. As a result, different dimensions are allowed to progress at different rates throughout the optimization process. Dimensions that have not yet converged continue exploring the search space, while those approaching convergence shift naturally toward exploitation. This adaptive behavior enables DASO to distribute the search effort more effectively, improving convergence stability and making the algorithm particularly suitable for high-dimensional optimization problems, where different decision variables often exhibit different convergence characteristics.

The updated position of both male and female individuals is defined using Eq. (43) and Eq. (44):

43 $$\:{X}_{i,m}\left(t\:+\:1\right)=\:{X}_{i,m}\left(t\right)+\:\:\eta\:\left(t\right)\odot\:{r}_{i}\left(t\right)\odot\:\left({X}_{food}\left(t\right)-{X}_{i,m}\left(t\right)\right)$$

44 $$\:{X}_{i,f}\left(t\:+\:1\right)=\:{X}_{i,f}\left(t\right)+\:\:\eta\:\left(t\right)\odot\:{r}_{i}\left(t\right)\odot\:\left({X}_{food}\left(t\right)-{X}_{i,f}\left(t\right)\right)$$

Where $\:{r}_{i}$ $\:\in\:[\mathrm{0,1}{]}^{D}$ introduces stochastic variability into the adaptive update while preserving the direction toward the current best solution. Applying independent random values to each decision prevents synchronized movements among individuals, enhances population diversity, and improves the robustness of the search process.

The adaptive coefficient is incorporated into the original Snake Optimizer update equations through element-wise multiplication, allowing the movement of each decision variable to be adjusted independently. Rather than applying a uniform search intensity across the entire solution vector, DASO continuously adapts the search scale according to the diversity observed in each dimension. This adaptive regulation reduces unnecessary oscillations in converged dimensions while preserving sufficient exploration in dimensions that still require further search. By adapting the search intensity separately for each decision variable instead of using a single global step size, DASO avoids unnecessary updates in already converged dimensions while continuing to explore dimensions that still contain useful search information. This selective adaptation improves search efficiency and contributes to the superior performance observed on high-dimensional benchmark problems.

**Fig. 3:**
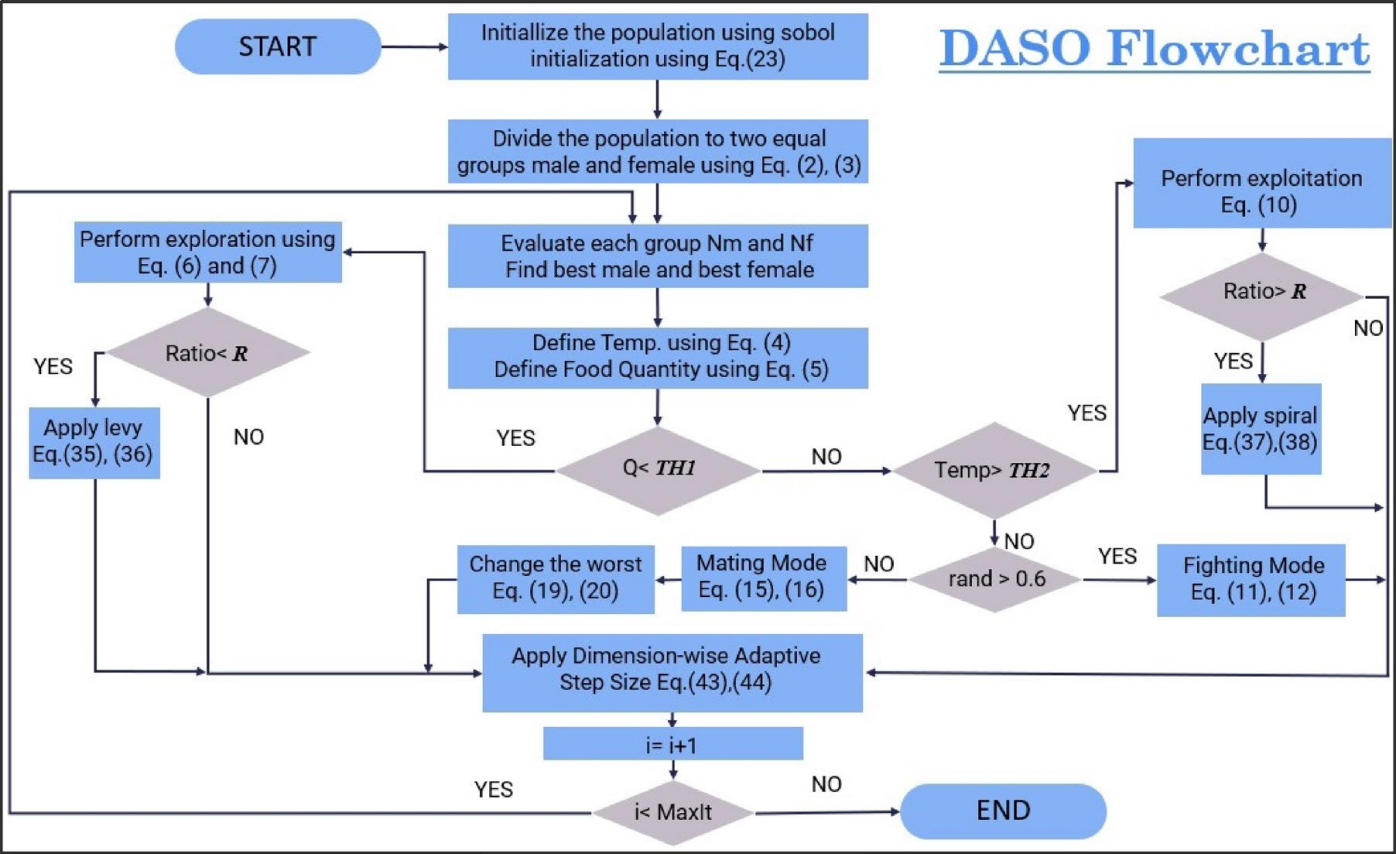
Flowchart of proposed DASO algorithm.

Figure 3 illustrates the flowchart of the proposed DASO, while Algorithm 2 shows the pseudo code of DASO.

**Algorithm 2:**
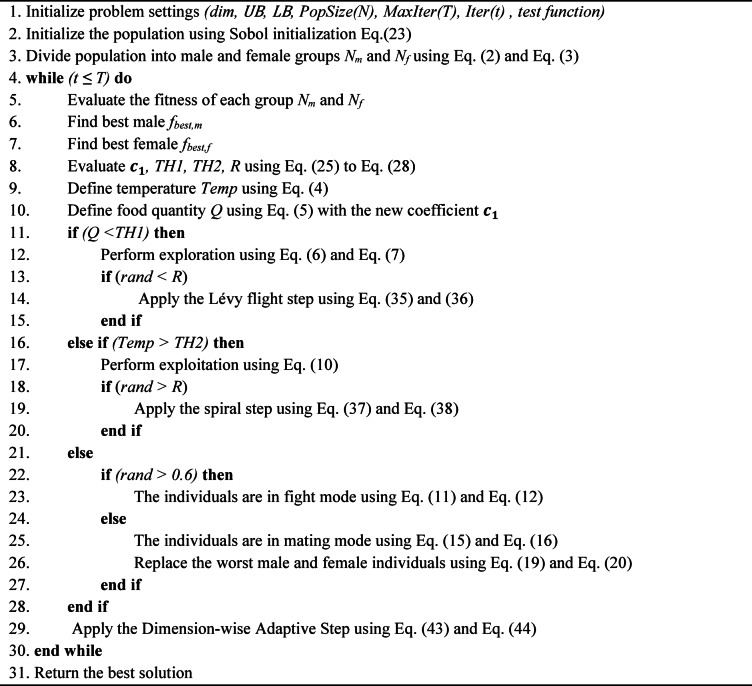
Dimension-wise Adaptive Snake Optimizer Algorithm (DASO).

### Computational complexity analysis

Computational complexity is an important criterion for evaluating the practicality and scalability of metaheuristic optimization algorithms. Although DASO introduces several enhancement strategies to improve the search behavior of the original Snake Optimizer (SO), these modifications are designed to enhance optimization performance without increasing the algorithm’s asymptotic computational complexity.

Let * N*denote the population size, * D*the number of decision variables, and * T*the maximum number of iterations. Similar to SO, the dominant computational cost of DASO comes from evaluating the objective function and updating the positions of all search agents during each iteration.

The initialization stage employs Sobol sequences to generate a uniformly distributed initial population. Since * N*candidate solutions are generated, each with * D*decision variables, the initialization requires

*O(ND).*

Because this step is executed only once before the optimization process begins, its contribution to the total computational cost is negligible.

During each iteration, the objective function is evaluated once for every search agent. If the computational cost of evaluating a single candidate solution is denoted by $\:{C}_{f}$, the complexity of the fitness evaluation is

$$O(NC_{f}).$$

The standard position update of the Snake Optimizer requires updating every decision variable for each search agent, resulting in a computational complexity of

*O(ND).*

The additional mechanisms introduced in DASO incur only lightweight computational overhead. The adaptive threshold is calculated using a few scalar arithmetic operations and therefore contributes a constant complexity of

*O(1).*

Similarly, both the Lévy-flight exploration strategy and the spiral exploitation strategy perform element-wise vector updates for all individuals, each requiring

*O(ND).*

The proposed dimension-wise adaptive step-size strategy first computes the variance of each decision variable across the entire population, which requires

*O(ND).*

The adaptive coefficients are then incorporated into the position update equations using element-wise operations, introducing another computational cost of

*O(ND).*

Therefore, the computational complexity of one optimization iteration can be expressed as

$$O(NC_{f})+O(ND)+O(1)+O(ND)+O(ND)+O(ND).$$

Combining similar terms yields

$$O(NC_{f}+ND).$$

Consequently, the overall computational complexity of DASO over * T*iterations becomes

$$O(T(NC_{f}+ND)).$$

For benchmark optimization problems, the cost of evaluating the objective function is generally proportional to the problem dimension ($\:{C}_{f}=O\left(D\right)$). Under this common assumption, the overall computational complexity can be simplified to

*O(TND),*

which is identical to that of the original Snake Optimizer.

Although DASO incorporates several adaptive mechanisms to improve the search process, these enhancements introduce only a small constant computational overhead while preserving the same asymptotic time complexity as SO. Consequently, the proposed algorithm achieves better convergence behavior and improved optimization performance without compromising computational scalability.

## Experimental results and analysis on different benchmarks

This section discusses the experimental outcomes from employing DASO on multiple benchmarks and comparing its performance to competing algorithms. We test the proposed DASO against other competing optimization techniques, namely the original SO^16^, GWO^12^, SCA^26^, HHO^27^, MFO^28^, and WOA^13^. To guarantee unbiased evaluation, each algorithm has undergone 30 separate runs with iteration count of 500. All algorithms had a unified population size of 30. The setting of parameters for each algorithm used in the experimental comparison can be found in Table 4 and is taken from the original manuscript of each algorithm. All the comparative experiments are executed on a computer with an AMD Ryzen 5 3600 6-Core Processor at 3.6 GHz with 16 GB of RAM, and the simulation platform utilized is MATLAB 2023b.

Non-parametric statistical tests were utilized to statistically evaluate the efficiency differences among the compared algorithms, due to the stochastic characteristics of metaheuristic optimization approaches. The Friedman test was initially applied for a comprehensive comparison across all benchmark functions to determine the occurrence of significant efficiency differences between the algorithms and the proposed DASO. Friedman test entails sorting each block of rows collectively, and subsequently employing the ranked column values. Therefore, the mean ranks of each algorithm is evaluated and sorted^62^. Thereafter, pairwise comparisons were performed utilizing the Wilcoxon signed-rank test with a significance threshold of $\:\alpha\:=0.05$. The Wilcoxon test was utilized to compare the proposed methodology against each competing methodology on a per-function basis^63^.

**Table 4:** Settings of the parameters for the compared algorithms.

Algorithm	Setting
SO	$\:{c}_{1\:}=0.5\:,\:\:\:{c}_{2}=0.05\:,\:\:\:{c}_{3}=2$
DASO	$\:{c}_{2}=0.05\:,{\:\:\:\:\:\:c}_{3}=2$
GWO	$\:{r}_{1}=rand\:\:,\:\:{\:\:\:\:r}_{2}=rand\:,\:\:c=2*{r}_{2}$, *a=2 (linearly decreases over iterations)*
SCA	*a = 2 (linearly decreases over iterations)*
HHO	$\:\beta\:=1.5$
MFO	* b=1*
WOA	$\:{r}_{1}=rand\:,\:\:{r}_{2}=rand\:,\:\:c=2*{r}_{2}$, *b =* 1, *a=2 (linearly decreases over iterations)*

Unless otherwise stated, all competing algorithms were implemented using the parameter settings recommended in their original publications to ensure a fair comparison. For DASO, the inherited parameters from the original Snake Optimizer were kept unchanged, while only the newly introduced parameters ($\:\alpha\:$ and $\:\beta\:$) were fixed throughout all experiments. Their values were selected based on preliminary sensitivity experiments and were subsequently used for all benchmark functions and PV parameter estimation without further tuning. The remaining adaptive components, including the adaptive threshold and the dimension-wise adaptive step-size mechanism, are computed dynamically during the optimization process and therefore do not require additional user-defined parameters.

### CEC 2017 benchmarks

The CEC2017 benchmark functions represent one of the most challenging benchmarks of evaluation^64^, and it consists of twenty-nine test functions in a variety of types, namely single-modal (F1), multi-modal (F3-F9), hybrid (F10-F19), or combination (F20-F30). In this evaluation, we used a variety of dimensions to evaluate the performance of DASO on low and high dimensional problems. The set dimensions are 10, 30, and 50.

#### DASO vs other algorithms on CEC2017 with a dimension of 10

**Fig. 4:**
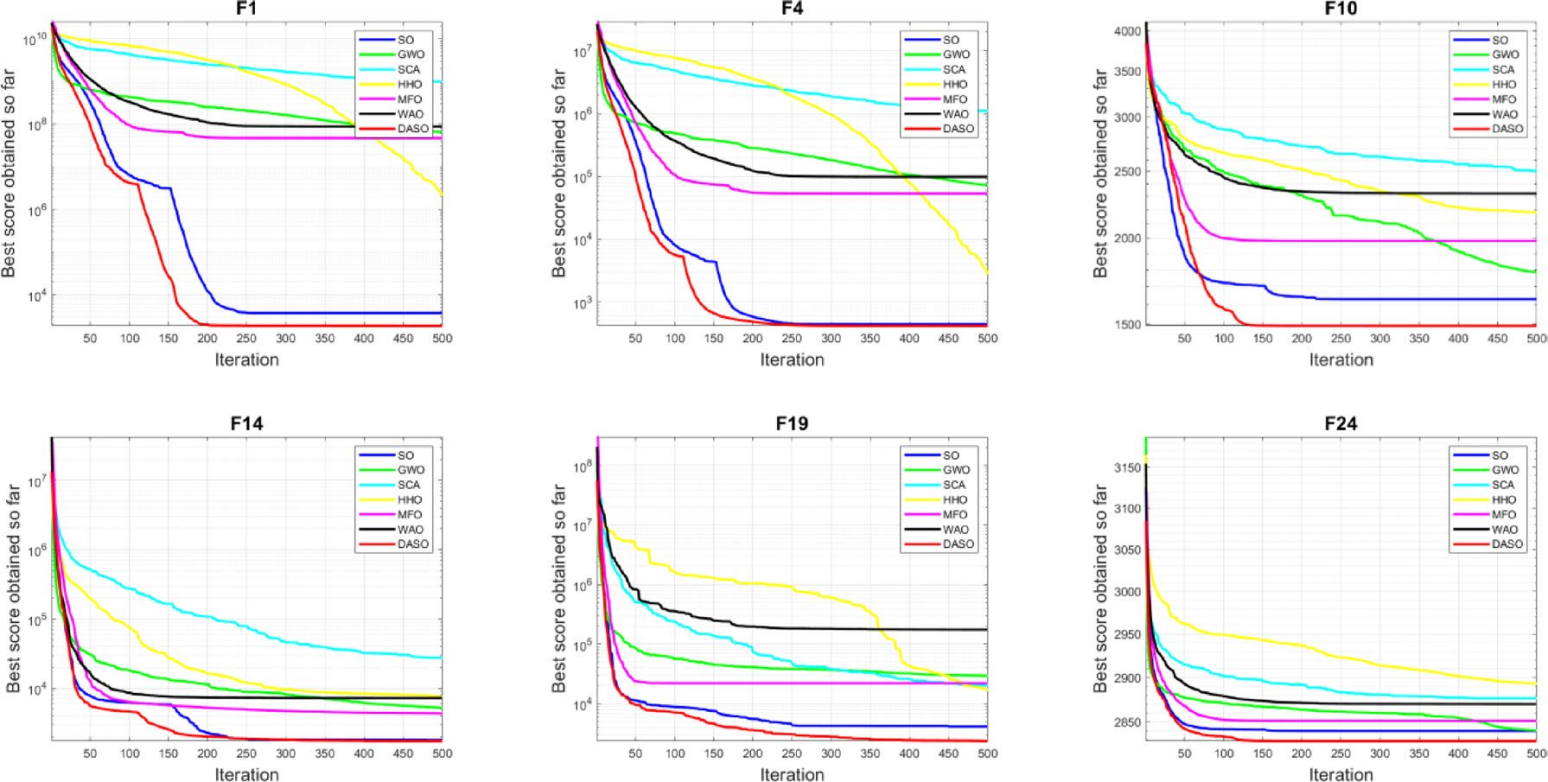
The samples of convergence curves for DASO vs. other algorithms on CEC2017 with Dim = 10.

Experimental findings pertaining to scalability challenges are presented in Table 5, which provides a summary of the optimal objective function, including its optimum value, mean, standard deviation, and the outcomes of the Wilcoxon and Friedman tests. A sample of the compared convergence curves of the various methods are shown in Fig. 4.

According to the results in Table 5, the average values attained by DASO are significantly higher than those obtained using the other algorithms considered in this study. The mean values for DASO ranked first over all the other method in all functions (25 out of 29 functions), except for F21, F27, and F28, DASO ranked second and it ranked third only for F26. DASO obtained an overall average rank of 1.17. This average rank indicates superior overall performance among the competing algorithms in this study. The Wilcoxon’s rank sum test counts are shown in Table 5, which indicates statistically significant superiority to the other competing algorithms.

**Table 5:** Comparison of results between DASO and other algorithms on CEC2017 with Dim = 10.

F(x)		SO	GWO	SCA	HHO	MFO	WOA	DASO
F1	Best	**1.053E + 02**	2.926E + 04	5.130E + 08	2.431E + 05	8.764E + 02	7.920E + 06	1.128E + 02
	Mean	3.678E + 03	6.538E + 07	9.942E + 08	2.105E + 06	4.735E + 07	8.785E + 07	**1.841E + 03**
	Std	4.390E + 03	1.344E + 08	3.919E + 08	4.447E + 06	2.464E + 08	1.104E + 08	**2.242E + 03**
	Rank	2.00	5.00	7.00	3.00	4.00	6.00	1.00
	Wilcoxon	=	+	+	+	+	+	–
	$\:\boldsymbol{p}$-value	5.98E-02	1.73E-06	1.73E-06	1.73E-06	4.73E-06	1.73E-06	–
F3	Best	3.032E + 02	3.922E + 02	1.144E + 03	3.309E + 02	3.003E + 02	6.491E + 02	**3.000E + 02**
	Mean	3.034E + 02	3.234E + 03	3.763E + 03	7.335E + 02	8.403E + 03	9.207E + 03	**3.00E + 02**
	Std	6.402E + 02	2.350E + 03	2.399E + 03	3.942E + 02	9.652E + 03	7.347E + 03	**2.976E + 02**
	Rank	2.00	4.00	5.00	3.00	6.00	7.00	1.00
	Wilcoxon	+	+	+	+	+	+	–
	$\:\boldsymbol{p}$-value	1.73E-06	1.73E-06	1.92E-06	3.11E-05	1.73E-06	1.73E-06	–
F4	Best	4.001E + 02	4.040E + 02	4.301E + 02	4.003E + 02	4.032E + 02	4.058E + 02	**4.000E + 02**
	Mean	4.036E + 02	4.209E + 02	4.757E + 02	4.349E + 02	4.309E + 02	4.649E + 02	**4.020E + 02**
	Std	1.108E + 01	2.100E + 01	4.531E + 01	4.176E + 01	3.617E + 01	4.737E + 01	**1.713E + 00**
	Rank	2.00	3.00	7.00	5.00	4.00	6.00	1.00
	Wilcoxon	+	+	+	+	+	+	–
	$\:\boldsymbol{p}$-value	1.17E-02	2.60E-06	1.73E-06	2.61E-04	2.60E-06	1.73E-06	–
F5	Best	5.080E + 02	5.071E + 02	5.309E + 02	5.164E + 02	5.099E + 02	5.300E + 02	**5.060E + 02**
	Mean	5.189E + 02	5.210E + 02	5.551E + 02	5.556E + 02	5.328E + 02	5.603E + 02	**5.132E + 02**
	Std	6.394E + 00	1.052E + 01	9.539E + 00	2.357E + 01	1.271E + 01	2.112E + 01	**5.201E + 00**
	Rank	2.00	3.00	5.00	6.00	4.00	7.00	1.00
	Wilcoxon	+	+	+	+	+	+	–
	$\:\boldsymbol{p}$-value	5.30E-05	1.71E-03	1.73E-06	2.35E-06	3.52E-06	1.73E-06	–
F6	Best	**6.000E + 02**	6.001E + 02	6.150E + 02	6.086E + 02	**6.000E + 02**	6.167E + 02	**6.000E + 02**
	Mean	6.011E + 02	6.015E + 02	6.237E + 02	6.386E + 02	6.030E + 02	6.388E + 02	**6.001E + 02**
	Std	2.319E + 00	2.101E + 00	5.045E + 00	1.120E + 01	3.412E + 00	1.635E + 01	**4.685E-01**
	Rank	2.00	3.00	5.00	6.00	4.00	7.00	1.00
	Wilcoxon	+	+	+	+	+	+	–
	$\:\boldsymbol{p}$-value	2.56E-02	1.74E-04	1.73E-06	1.73E-06	2.84E-05	1.73E-06	–
F7	Best	7.204E + 02	7.160E + 02	7.649E + 02	7.437E + 02	7.221E + 02	7.410E + 02	**7.149E + 02**
	Mean	7.355E + 02	7.363E + 02	7.832E + 02	7.875E + 02	7.394E + 02	7.862E + 02	**7.284E + 02**
	Std	1.394E + 01	1.627E + 01	1.020E + 01	2.103E + 01	1.164E + 01	2.500E + 01	**8.611E + 00**
	Rank	2.00	3.00	5.00	7.00	4.00	6.00	1.00
	Wilcoxon	+	=	+	+	+	+	–
	$\:\boldsymbol{p}$-value	8.21e-03	6.87E-02	1.73E-06	1.73E-06	1.48E-03	1.73E-06	–
F8	Best	8.080E + 02	8.061E + 02	8.298E + 02	8.134E + 02	8.070E + 02	8.180E + 02	**8.060E + 02**
	Mean	8.194E + 02	8.173E + 02	8.468E + 02	8.312E + 02	8.302E + 02	8.463E + 02	**8.164E + 02**
	Std	6.676E + 00	**6.549E + 00**	9.528E + 00	8.771E + 00	1.465E + 01	1.909E + 01	7.035E + 00
	Rank	3.00	2.00	7.00	5.00	4.00	6.00	1.00
	Wilcoxon	+	=	+	+	+	+	–
	$\:\boldsymbol{p}$-value	4.49E-02	8.61E-01	1.73E-06	6.34E-06	2.05E-04	2.13E-06	–
F9	Best	9.000E + 02	9.001E + 02	9.633E + 02	1.006E + 03	9.000E + 02	1.023E + 03	**9.000E + 02**
	Mean	9.211E + 02	9.186E + 02	1.061E + 03	1.443E + 03	9.743E + 02	1.531E + 03	**9.042E + 02**
	Std	2.894E + 01	2.534E + 01	7.185E + 01	2.471E + 02	1.025E + 02	5.523E + 02	**8.042E + 00**
	Rank	3.00	2.00	5.00	6.00	4.00	7.00	1.00
	Wilcoxon	+	=	+	+	+	+	–
	$\:\boldsymbol{p}$-value	3.85E-03	1.20E-01	1.73E-06	1.73E-06	1.71E-03	1.73E-06	–
F10	Best	1.142E + 03	**1.016E + 03**	1.828E + 03	1.463E + 03	1.285E + 03	1.543E + 03	1.155E + 03
	Mean	1.599E + 03	1.753E + 03	2.469E + 03	2.131E + 03	1.949E + 03	2.270E + 03	**1.460E + 03**
	Std	**1.980E + 02**	3.880E + 02	2.205E + 02	2.939E + 02	3.330E + 02	3.350E + 02	2.140E + 02
	Rank	2.00	3.00	7.00	5.00	4.00	6.00	1.00
	Wilcoxon	+	+	+	+	+	+	–
	$\:\boldsymbol{p}$-value	2.07E-02	2.11E-03	1.73E-06	2.35E-06	1.13E-05	1.73E-06	–
F11	Best	1.104E + 03	1.109E + 03	1.174E + 03	1.114E + 03	1.106E + 03	1.137E + 03	**1.103E + 03**
	Mean	1.123E + 03	1.136E + 03	1.245E + 03	1.173E + 03	1.344E + 03	1.247E + 03	**1.114E + 03**
	Std	1.313E + 01	2.442E + 01	6.653E + 01	4.763E + 01	5.040E + 02	1.090E + 02	**1.249E + 01**
	Rank	2.00	3.00	5.00	4.00	7.00	6.00	1.00
	Wilcoxon	+	+	+	+	+	+	–
	$\:\boldsymbol{p}$-value	3.00E-02	5.31E-05	1.73E-06	2.88E-06	1.89E-04	1.73E-06	–
F12	Best	2.225E + 03	1.454E + 04	2.876E + 06	7.353E + 04	**1.932E + 03**	7.052E + 04	2.426E + 03
	Mean	2.938E + 04	7.166E + 05	2.066E + 07	4.517E + 06	9.054E + 05	3.224E + 06	**1.323E + 04**
	Std	**4.281E + 04**	7.994E + 05	1.348E + 07	5.193E + 06	1.927E + 06	3.981E + 06	7.231E + 04
	Rank	2.00	3.00	7.00	6.00	4.00	5.00	1.00
	Wilcoxon	=	+	+	+	+	+	–
	$\:\boldsymbol{p}$-value	4.17E-01	8.47E-06	1.73E-06	1.73E-06	2.61E-04	1.73E-06	–
F13	Best	1.361E + 03	3.168E + 03	9.135E + 03	3.454E + 03	**1.311E + 03**	1.957E + 03	1.555E + 03
	Mean	4.482E + 03	1.234E + 04	6.954E + 04	1.786E + 04	1.231E + 04	1.967E + 04	**4.286E + 03**
	Std	5.389E + 03	7.722E + 03	5.380E + 04	1.712E + 04	1.192E + 04	1.537E + 04	**4.264E + 03**
	Rank	2.00	4.00	7.00	5.00	3.00	6.00	1.00
	Wilcoxon	=	+	+	+	=	+	–
	$\:\boldsymbol{p}$-value	1.99E-01	3.85E-03	2.13E-06	1.89E-04	6.87E-02	4.53E-04	–
F14	Best	**1.425E + 03**	1.473E + 03	1.529E + 03	1.492E + 03	1.481E + 03	1.477E + 03	1.434E + 03
	Mean	1.504E + 03	4.032E + 03	2.415E + 03	1.961E + 03	2.920E + 03	2.976E + 03	**1.461E + 03**
	Std	9.201E + 01	2.032E + 03	1.003E + 03	8.578E + 02	1.455E + 03	1.838E + 03	**7.709E + 01**
	Rank	2.00	7.00	4.00	3.00	5.00	6.00	1.00
	Wilcoxon	+	+	+	+	+	+	–
	$\:\boldsymbol{p}$-value	1.11E-03	8.47E-06	2.60E-06	1.92E-06	1.92E-06	4.29E-06	–
F15	Best	1.557E + 03	1.581E + 03	1.678E + 03	2.028E + 03	1.840E + 03	1.593E + 03	**1.541E + 03**
	Mean	1.856E + 03	7.397E + 03	3.775E + 03	7.764E + 03	7.535E + 03	1.033E + 04	**1.616E + 03**
	Std	3.543E + 02	5.894E + 03	1.706E + 03	3.326E + 03	7.552E + 03	8.915E + 03	**2.173E + 02**
	Rank	2.00	4.00	3.00	6.00	5.00	7.00	1.00
	Wilcoxon	+	+	+	+	+	+	–
	$\:\boldsymbol{p}$-value	8.18E-05	3.88E-06	1.92E-06	1.73E-06	1.92E-06	3.18E-06	–
F16	Best	1.603E + 03	1.634E + 03	1.623E + 03	1.730E + 03	**1.601E + 03**	1.609E + 03	**1.601E + 03**
	Mean	1.722E + 03	1.788E + 03	1.800E + 03	1.940E + 03	1.792E + 03	1.921E + 03	**1.649E + 03**
	Std	1.003E + 02	1.417E + 02	**8.649E + 01**	1.073E + 02	1.422E + 02	1.475E + 02	9.694E + 01
	Rank	2.00	3.00	5.00	7.00	4.00	6.00	1.00
	Wilcoxon	+	+	+	+	+	+	–
	$\:\boldsymbol{p}$-value	1.31E-02	7.27E-03	1.36E-05	1.73E-06	4.99E-03	3.52E-06	–
F17	Best	**1.703E + 03**	1.734E + 03	1.767E + 03	1.744E + 03	1.721E + 03	1.735E + 03	1.718E + 03
	Mean	1.761E + 03	1.768E + 03	1.796E + 03	1.786E + 03	1.769E + 03	1.813E + 03	**1.736E + 03**
	Std	5.964E + 01	1.992E + 01	1.602E + 01	5.015E + 01	4.690E + 01	6.325E + 01	**1.458E + 01**
	Rank	2.00	3.00	6.00	5.00	4.00	7.00	1.00
	Wilcoxon	+	+	+	+	+	+	–
	$\:\boldsymbol{p}$-value	5.32E-03	8.47E-06	1.73E-06	2.88E-06	2.05E-04	2.13E-06	–
F18	Best	**2.048E + 03**	4.372E + 03	5.066E + 04	2.275E + 03	3.118E + 03	3.136E + 03	2.147E + 03
	Mean	7.807E + 03	2.796E + 04	2.991E + 05	1.623E + 04	2.665E + 04	1.703E + 04	**4.845E + 03**
	Std	**4.525E + 03**	1.462E + 04	3.264E + 05	1.117E + 04	1.591E + 04	1.267E + 04	4.967E + 03
	Rank	2.00	6.00	7.00	3.00	5.00	4.00	1.00
	Wilcoxon	+	+	+	+	+	+	–
	$\:\boldsymbol{p}$-value	1.85E-02	3.88E-06	1.73E-06	2.26E-03	1.49E-05	2.58E-03	–
F19	Best	1.920E + 03	1.962E + 03	2.326E + 03	2.518E + 03	2.094E + 03	2.376E + 03	**1.912E + 03**
	Mean	2.614E + 03	2.847E + 04	1.011E + 04	1.656E + 04	2.133E + 04	1.744E + 05	**1.983E + 03**
	Std	3.053E + 03	5.968E + 04	7.890E + 03	1.315E + 04	2.121E + 04	5.393E + 05	**1.913E + 02**
	Rank	2.00	6.00	3.00	4.00	5.00	7.00	1.00
	Wilcoxon	+	+	+	+	+	+	–
	$\:\boldsymbol{p}$-value	4.07E-05	2.88E-06	1.73E-06	1.73E-06	1.73E-06	1.73E-06	–
F20	Best	2.010E + 03	2.024E + 03	2.071E + 03	2.072E + 03	2.001E + 03	2.074E + 03	**2.000E + 03**
	Mean	2.051E + 03	2.096E + 03	2.132E + 03	2.193E + 03	2.104E + 03	2.214E + 03	**2.029E + 03**
	Std	3.573E + 01	5.531E + 01	3.594E + 01	6.595E + 01	7.618E + 01	7.565E + 01	**1.553E + 01**
	Rank	2.00	3.00	5.00	6.00	4.00	7.00	1.00
	Wilcoxon	+	+	+	+	+	+	–
	$\:\boldsymbol{p}$-value	4.99E-03	1.36E-05	1.73E-06	1.73E-06	1.49E-05	1.73E-06	–
F21	Best	2.309E + 03	2.205E + 03	2.210E + 03	2.208E + 03	2.206E + 03	2.214E + 03	**2.202E + 03**
	Mean	2.322E + 03	2.314E + 03	**2.276E + 03**	2.330E + 03	2.323E + 03	2.327E + 03	2.309E + 03
	Std	**8.413E + 00**	2.202E + 01	6.327E + 01	5.668E + 01	3.844E + 01	5.676E + 01	2.928E + 01
	Rank	4.00	3.00	1.00	7.00	5.00	6.00	2.00
	Wilcoxon	+	=	-	+	+	=	–
	$\:\boldsymbol{p}$-value	2.11E-03	5.30E-01	2.07E-02	3.50E-02	4.99E-03	5.45E-02	–
F22	Best	2.301E + 03	**2.227E + 03**	2.315E + 03	2.307E + 03	2.238E + 03	2.264E + 03	2.268E + 03
	Mean	2.302E + 03	2.306E + 03	2.414E + 03	2.315E + 03	2.307E + 03	2.324E + 03	**2.301E + 03**
	Std	**7.090E-01**	1.691E + 01	4.471E + 01	4.065E + 00	1.956E + 01	1.621E + 01	6.326E + 00
	Rank	2.00	3.00	7.00	5.00	4.00	6.00	1.00
	Wilcoxon	=	+	+	+	+	+	–
	$\:\boldsymbol{p}$-value	2.80E-01	5.79E-05	1.73E-06	1.73E-06	6.42E-03	1.97E-05	–
F23	Best	2.610E + 03	**2.603E + 03**	2.648E + 03	2.617E + 03	2.617E + 03	2.620E + 03	2.606E + 03
	Mean	2.625E + 03	2.628E + 03	2.665E + 03	2.680E + 03	2.630E + 03	2.656E + 03	**2.616E + 03**
	Std	1.072E + 01	1.267E + 01	1.071E + 01	2.884E + 01	8.703E + 00	2.668E + 01	**7.894E + 00**
	Rank	2.00	3.00	6.00	7.00	4.00	5.00	1.00
	Wilcoxon	+	+	+	+	+	+	–
	$\:\boldsymbol{p}$-value	7.71E-04	9.84E-03	1.73E-06	2.35E-06	2.22E-04	1.73E-06	–
F24	Best	2.740E + 03	2.731E + 03	2.570E + 03	2.502E + 03	2.577E + 03	2.573E + 03	**2.500E + 03**
	Mean	2.753E + 03	2.750E + 03	2.785E + 03	2.801E + 03	2.761E + 03	2.779E + 03	**2.739E + 03**
	Std	**7.959E + 00**	1.439E + 01	4.174E + 01	1.303E + 02	3.615E + 01	5.524E + 01	4.558E + 01
	Rank	3.00	2.00	6.00	7.00	4.00	5.00	1.00
	Wilcoxon	+	=	+	+	+	+	–
	$\:\boldsymbol{p}$-value	2.58E-03	2.29E-01	2.84E-05	1.17E-02	2.84E-05	3.06E-04	–
F25	Best	2.800E + 03	2.914E + 03	2.949E + 03	**2.616E + 03**	2.898E + 03	2.693E + 03	2.898E + 03
	Mean	2.923E + 03	2.934E + 03	2.982E + 03	2.928E + 03	2.944E + 03	2.969E + 03	**2.920E + 03**
	Std	3.206E + 01	**1.353E + 01**	2.077E + 01	6.580E + 01	2.633E + 01	6.846E + 01	2.361E + 01
	Rank	2.00	4.00	7.00	3.00	5.00	6.00	1.00
	Wilcoxon	=	+	+	+	+	+	–
	$\:\boldsymbol{p}$-value	5.04E-01	2.07E-02	1.73E-06	1.11E-03	2.61E-04	6.89E-05	–
F26	Best	**2.600E + 03**	2.814E + 03	3.034E + 03	2.825E + 03	2.900E + 03	2.901E + 03	2.800E + 03
	Mean	3.243E + 03	3.052E + 03	3.117E + 03	3.547E + 03	**3.022E + 03**	3.523E + 03	3.107E + 03
	Std	4.455E + 02	2.794E + 02	**4.119E + 01**	5.712E + 02	6.786E + 01	5.173E + 02	2.517E + 02
	Rank	5.00	2.00	4.00	7.00	1.00	6.00	3.00
	Wilcoxon	+	=	=	+	-	+	–
	$\:\boldsymbol{p}$-value	3.33E-02	1.31E-01	1.36E-01	2.96E-03	2.56E-02	1.36E-04	–
F27	Best	3.097E + 03	**3.089E + 03**	3.101E + 03	3.100E + 03	3.091E + 03	3.097E + 03	3.092E + 03
	Mean	3.107E + 03	3.108E + 03	3.106E + 03	3.173E + 03	**3.096E + 03**	3.138E + 03	3.101E + 03
	Std	1.223E + 01	2.473E + 01	**2.713E + 00**	7.140E + 01	4.109E + 00	4.349E + 01	7.396E + 00
	Rank	5.00	4.00	3.00	7.00	1.00	6.00	2.00
	Wilcoxon	+	=	=	+	-	+	–
	$\:\boldsymbol{p}$-value	1.59E-03	4.28E-01	6.56E-02	1.73E-06	7.69E-06	1.06E-04	–
F28	Best	**3.100E + 03**	3.101E + 03	3.234E + 03	3.166E + 03	3.172E + 03	3.194E + 03	3.169E + 03
	Mean	**3.324E + 03**	3.364E + 03	3.342E + 03	3.407E + 03	3.366E + 03	3.501E + 03	3.340E + 03
	Std	1.237E + 02	1.071E + 02	7.739E + 01	1.368E + 02	9.855E + 01	1.858E + 02	**7.337E + 01**
	Rank	1.00	4.00	3.00	6.00	5.00	7.00	2.00
	Wilcoxon	-	=	=	=	=	+	–
	$\:\boldsymbol{p}$-value	3.69E-02	6.58E-01	5.45E-02	3.82E-01	3.93E-01	2.11E-03	–
F29	Best	3.153E + 03	3.152E + 03	3.188E + 03	3.188E + 03	**3.141E + 03**	3.195E + 03	3.153E + 03
	Mean	3.213E + 03	3.204E + 03	3.272E + 03	3.389E + 03	3.246E + 03	3.393E + 03	**3.191E + 03**
	Std	3.650E + 01	5.427E + 01	6.005E + 01	1.068E + 02	7.235E + 01	1.310E + 02	**3.344E + 01**
	Rank	3.00	2.00	5.00	6.00	4.00	7.00	1.00
	Wilcoxon	+	=	+	+	+	+	–
	$\:\boldsymbol{p}$-value	1.75E-02	5.72E-01	1.36E-05	1.92E-06	1.38E-03	2.35E-06	–
F30	Best	5.252E + 03	4.334E + 03	8.697E + 04	1.643E + 04	4.393E + 03	1.086E + 04	**3.636E + 03**
	Mean	1.866E + 05	8.411E + 05	1.547E + 06	9.334E + 05	6.847E + 05	2.044E + 06	**9.165E + 04**
	Std	4.813E + 05	1.134E + 06	7.673E + 05	1.090E + 06	6.299E + 05	2.013E + 06	**2.473E + 05**
	Rank	2.00	4.00	6.00	5.00	3.00	7.00	1.00
	Wilcoxon	+	+	+	+	+	+	–
	$\:\boldsymbol{p}$-value	5.29E-04	3.41E-05	1.73E-06	3.11E-05	1.80E-05	3.88E-06	–
Wilcoxon Win/Tie/Loss		23/5/1	20/9/0	25/3/1	28/1/0	25/2/2	28/1/0	–
Avg Rank		2.37	3.48	5.27	5.34	4.14	6.21	**1.17**
Friedman rank		2	3	5	6	4	7	**1**
Friedman$\:\boldsymbol{p}$-value		* 1.046E-21*						
Friedman$\:{\boldsymbol{\chi\:}}^{2}$		111.3842						

#### DASO vs other algorithms on CEC2017 with a dimension of 30

**Fig. 5:**
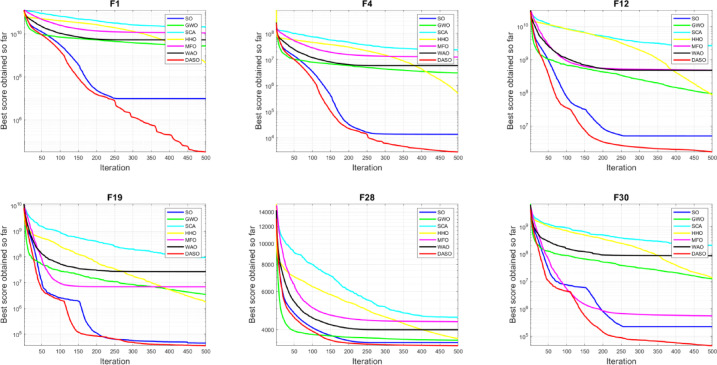
The samples of convergence curves for DASO vs other algorithms on CEC2017 with Dim= 30.

Experimental results of DASO against other algorithms on CEC2017 with 30 dimension are displayed in Table 6, which presents a summary of the optimal objective function, including its optimum value, mean, standard deviation, and the outcomes of the Wilcoxon and Friedman tests. A sample of the generated convergence curves for DASO against the compared algorithms are shown in Fig. 5.

As shown in Table 6, the mean values evaluated by DASO are significantly higher than those calculated using the other algorithms considered in this study. The mean values for DASO ranked first over all the other method in all functions (26 out of 29 functions), except for F10, F17, and F26, DASO ranked second. DASO achieved an average rank of 1.1, which indicates superior overall performance among the algorithms considered. The Wilcoxon’s rank sum test counts are shown in Table 6. These values indicate that DASO is statistically significant compared to the competing algorithms in this study.

**Table 6:** Comparison of results between DASO and other algorithms on CEC2017 with Dim = 30.

F(x)		SO	GWO	SCA	HHO	MFO	WOA	DASO
F1	Best	3.261E + 05	6.226E + 08	1.598E + 10	1.043E + 08	9.222E + 05	2.109E + 09	**3.976E + 02**
	Mean	9.726E + 06	2.765E + 09	2.127E + 10	4.596E + 08	1.123E + 10	5.294E + 09	**3.221E + 04**
	Std	1.393E + 07	1.697E + 09	3.434E + 09	3.495E + 08	1.036E + 10	2.007E + 09	**9.149E + 04**
	Rank	2.00	4.00	7.00	3.00	6.00	5.00	1.00
	Wilcoxon	+	+	+	+	+	+	–
	$\:\boldsymbol{p}$-value	1.73E-06	1.73E-06	1.73E-06	1.73E-06	1.73E-06	1.73E-06	–
F3	Best	5.618E + 04	3.215E + 04	5.862E + 04	4.401E + 04	7.324E + 04	1.415E + 05	**2.107E + 04**
	Mean	6.571E + 04	6.562E + 04	8.825E + 04	5.707E + 04	1.750E + 05	2.620E + 05	**2.675E + 04**
	Std	7.204E + 03	1.431E + 04	1.998E + 04	**6.856E + 03**	6.302E + 04	7.721E + 04	1.675E + 04
	Rank	4.00	3.00	5.00	2.00	6.00	7.00	1.00
	Wilcoxon	+	=	+	=	+	+	–
	$\:\boldsymbol{p}$-value	2.60E-06	1.78E-01	8.94E-04	1.89E-04	1.92E-06	1.73E-06	–
F4	Best	4.899E + 02	4.984E + 02	1.787E + 03	5.852E + 02	5.017E + 02	7.754E + 02	**4.737E + 02**
	Mean	5.588E + 02	6.838E + 02	3.281E + 03	7.103E + 02	1.053E + 03	1.336E + 03	**5.043E + 02**
	Std	4.531E + 01	3.529E + 02	1.020E + 03	8.479E + 01	6.985E + 02	3.409E + 02	**2.951E + 01**
	Rank	2.00	3.00	7.00	4.00	5.00	6.00	1.00
	Wilcoxon	+	+	+	+	+	+	–
	$\:\boldsymbol{p}$-value	1.74E-04	3.52E-06	1.73E-06	1.73E-06	6.34E-06	1.73E-06	–
F5	Best	5.623E + 02	5.754E + 02	7.711E + 02	7.320E + 02	6.369E + 02	7.974E + 02	**5.613E + 02**
	Mean	6.005E + 02	6.172E + 02	8.201E + 02	7.772E + 02	7.023E + 02	8.938E + 02	**5.786E + 02**
	Std	2.315E + 01	3.105E + 01	2.406E + 01	2.500E + 01	4.470E + 01	6.453E + 01	**1.498E + 01**
	Rank	2.00	3.00	6.00	5.00	4.00	7.00	1.00
	Wilcoxon	+	+	+	+	+	+	–
	$\:\boldsymbol{p}$-value	1.11E-03	3.11E-05	1.73E-06	1.73E-06	1.73E-06	1.73E-06	–
F6	Best	6.064E + 02	6.069E + 02	6.552E + 02	6.561E + 02	6.168E + 02	6.552E + 02	**6.020E + 02**
	Mean	6.186E + 02	6.135E + 02	6.667E + 02	6.691E + 02	6.360E + 02	6.847E + 02	**6.082E + 02**
	Std	7.699E + 00	3.998E + 00	9.414E + 00	5.117E + 00	1.100E + 01	1.757E + 01	**3.956E + 00**
	Rank	3.00	2.00	5.00	6.00	4.00	7.00	1.00
	Wilcoxon	+	+	+	+	+	+	–
	$\:\boldsymbol{p}$-value	6.98E-06	9.71E-05	1.73E-06	1.73E-06	1.73E-06	1.73E-06	–
F7	Best	8.221E + 02	8.446E + 02	1.114E + 03	1.211E + 03	7.973E + 02	1.138E + 03	**7.789E + 02**
	Mean	8.709E + 02	9.028E + 02	1.235E + 03	1.346E + 03	1.137E + 03	1.290E + 03	**8.374E + 02**
	Std	4.582E + 01	4.599E + 01	5.967E + 01	6.370E + 01	2.317E + 02	8.359E + 01	**3.143E + 01**
	Rank	2.00	3.00	5.00	7.00	4.00	6.00	1.00
	Wilcoxon	+	+	+	+	+	+	–
	$\:\boldsymbol{p}$-value	2.58E-03	4.07E-05	1.73E-06	1.73E-06	2.88E-06	1.73E-06	–
F8	Best	8.733E + 02	8.573E + 02	1.051E + 03	9.545E + 02	9.403E + 02	9.760E + 02	**8.455E + 02**
	Mean	9.011E + 02	9.056E + 02	1.100E + 03	9.904E + 02	9.998E + 02	1.083E + 03	**8.744E + 02**
	Std	1.801E + 01	2.914E + 01	2.279E + 01	2.379E + 01	4.107E + 01	4.498E + 01	**1.494E + 01**
	Rank	2.00	3.00	7.00	4.00	5.00	6.00	1.00
	Wilcoxon	+	+	+	+	+	+	–
	$\:\boldsymbol{p}$-value	8.47E-06	3.41E-05	1.73E-06	1.73E-06	1.73E-06	1.73E-06	–
F9	Best	1.338E + 03	1.262E + 03	5.466E + 03	6.281E + 03	3.306E + 03	5.747E + 03	**1.061E + 03**
	Mean	1.941E + 03	2.887E + 03	7.757E + 03	8.880E + 03	7.116E + 03	1.174E + 04	**1.445E + 03**
	Std	7.316E + 02	1.406E + 03	1.710E + 03	1.132E + 03	2.334E + 03	4.135E + 03	**4.427E + 02**
	Rank	2.00	3.00	5.00	6.00	4.00	7.00	1.00
	Wilcoxon	+	+	+	+	+	+	–
	$\:\boldsymbol{p}$-value	6.64E-04	6.34E-06	1.73E-06	1.73E-06	1.73E-06	1.73E-06	–
F10	Best	3.212E + 03	3.396E + 03	7.965E + 03	4.832E + 03	3.775E + 03	4.844E + 03	**2.928E + 03**
	Mean	**4.282E + 03**	5.389E + 03	8.834E + 03	6.080E + 03	5.559E + 03	7.592E + 03	4.686E + 03
	Std	6.778E + 02	1.699E + 03	**3.696E + 02**	7.174E + 02	8.151E + 02	7.657E + 02	1.060E + 03
	Rank	1.00	3.00	7.00	5.00	4.00	6.00	2.00
	Wilcoxon	=	=	+	+	+	+	–
	$\:\boldsymbol{p}$-value	1.85E-01	9.78E-02	1.73E-06	1.64E-05	1.38E-03	1.92E-06	–
F11	Best	1.289E + 03	1.373E + 03	2.222E + 03	1.282E + 03	1.449E + 03	3.762E + 03	**1.225E + 03**
	Mean	1.451E + 03	2.439E + 03	4.118E + 03	1.561E + 03	4.399E + 03	1.092E + 04	**1.297E + 03**
	Std	9.832E + 01	1.016E + 03	1.039E + 03	1.741E + 02	4.513E + 03	4.273E + 03	**6.841E + 01**
	Rank	2.00	4.00	5.00	3.00	6.00	7.00	1.00
	Wilcoxon	+	+	+	+	+	+	–
	$\:\boldsymbol{p}$-value	3.11E-05	1.73E-06	1.73E-06	1.73E-06	1.73E-06	1.73E-06	–
F12	Best	1.662E + 05	1.002E + 07	1.159E + 09	7.917E + 06	1.266E + 06	9.137E + 07	**1.033E + 05**
	Mean	5.067E + 06	9.566E + 07	2.629E + 09	8.753E + 07	4.783E + 08	4.757E + 08	**1.679E + 06**
	Std	4.719E + 06	9.483E + 07	8.896E + 08	7.273E + 07	8.439E + 08	2.449E + 08	**2.182E + 06**
	Rank	2.00	4.00	7.00	3.00	6.00	5.00	1.00
	Wilcoxon	+	+	+	+	+	+	–
	$\:\boldsymbol{p}$-value	2.41E-04	1.73E-06	1.73E-06	1.73E-06	2.13E-06	1.73E-06	–
F13	Best	6.385E + 03	6.050E + 04	4.089E + 08	1.082E + 05	1.866E + 04	5.222E + 05	**3.776E + 03**
	Mean	5.042E + 04	3.313E + 07	1.161E + 09	1.987E + 06	7.613E + 07	9.991E + 06	**3.383E + 04**
	Std	3.273E + 04	7.377E + 07	4.060E + 08	3.476E + 06	2.679E + 08	7.558E + 06	**3.099E + 04**
	Rank	2.00	5.00	7.00	3.00	6.00	4.00	1.00
	Wilcoxon	+	+	+	+	+	+	–
	$\:\boldsymbol{p}$-value	3.87E-02	1.73E-06	1.73E-06	1.73E-06	2.13E-06	1.73E-06	–
F14	Best	**2.731E + 03**	1.088E + 04	2.127E + 05	2.180E + 04	1.016E + 04	6.763E + 04	4.857E + 03
	Mean	7.794E + 04	7.940E + 05	7.941E + 05	1.491E + 06	4.840E + 05	2.343E + 06	**3.281E + 04**
	Std	1.106E + 05	1.208E + 06	4.263E + 05	1.274E + 06	6.539E + 05	2.259E + 06	**4.060E + 04**
	Rank	2.00	4.00	5.00	6.00	3.00	7.00	1.00
	Wilcoxon	+	+	+	+	+	+	–
	$\:\boldsymbol{p}$-value	3.00E-02	1.80E-05	1.73E-06	2.35E-06	4.73E-06	1.73E-06	–
F15	Best	2.990E + 03	1.157E + 04	2.356E + 06	3.355E + 04	6.991E + 03	1.499E + 05	**2.027E + 03**
	Mean	1.634E + 04	8.500E + 05	6.351E + 07	1.246E + 05	5.557E + 04	6.143E + 06	**1.164E + 04**
	Std	**1.368E + 04**	1.527E + 06	5.137E + 07	6.655E + 04	4.734E + 04	7.846E + 06	1.794E + 04
	Rank	2.00	5.00	7.00	4.00	3.00	6.00	1.00
	Wilcoxon	+	+	+	+	+	+	–
	$\:\boldsymbol{p}$-value	3.50E-02	5.75E-06	1.73E-06	1.92E-06	3.72E-05	1.73E-06	–
F16	Best	2.145E + 03	2.048E + 03	3.625E + 03	2.883E + 03	2.418E + 03	3.158E + 03	**1.868E + 03**
	Mean	2.599E + 03	2.615E + 03	4.158E + 03	3.658E + 03	3.079E + 03	4.163E + 03	**2.426E + 03**
	Std	**2.174E + 02**	2.565E + 02	3.081E + 02	5.014E + 02	3.912E + 02	6.037E + 02	2.317E + 02
	Rank	2.00	3.00	6.00	5.00	4.00	7.00	1.00
	Wilcoxon	+	+	+	+	+	+	–
	$\:\boldsymbol{p}$-value	1.66E-02	1.04E-02	1.73E-06	1.73E-06	5.22E-06	1.73E-06	–
F17	Best	1.849E + 03	**1.815E + 03**	2.471E + 03	2.299E + 03	2.211E + 03	2.300E + 03	1.829E + 03
	Mean	2.261E + 03	**2.112E + 03**	2.904E + 03	2.736E + 03	2.626E + 03	2.773E + 03	2.142E + 03
	Std	2.216E + 02	2.018E + 02	1.912E + 02	2.549E + 02	2.094E + 02	2.673E + 02	**1.676E + 02**
	Rank	3.00	1.00	7.00	5.00	4.00	6.00	2.00
	Wilcoxon	+	=	+	+	+	+	–
	$\:\boldsymbol{p}$-value	8.73E-03	9.43E-01	1.73E-06	1.73E-06	1.92E-06	1.73E-06	–
F18	Best	7.892E + 04	8.731E + 04	2.850E + 06	4.241E + 05	1.262E + 05	3.180E + 05	**6.214E + 04**
	Mean	9.887E + 05	1.848E + 06	1.643E + 07	5.545E + 06	7.328E + 06	1.060E + 07	**5.295E + 05**
	Std	1.108E + 06	2.446E + 06	1.085E + 07	6.208E + 06	1.162E + 07	9.503E + 06	**1.024E + 06**
	Rank	2.00	3.00	7.00	4.00	5.00	6.00	1.00
	Wilcoxon	=	+	+	+	+	+	–
	$\:\boldsymbol{p}$-value	8.29E-01	3.50E-02	1.73E-06	1.97E-05	6.98E-06	2.35E-06	–
F19	Best	2.220E + 03	1.060E + 04	3.225E + 07	1.583E + 05	4.031E + 03	1.224E + 06	**2.015E + 03**
	Mean	1.176E + 04	3.411E + 06	9.338E + 07	1.558E + 06	6.631E + 06	2.621E + 07	**9.551E + 03**
	Std	1.026E + 04	7.801E + 06	5.367E + 07	1.251E + 06	1.867E + 07	2.373E + 07	**6.419E + 03**
	Rank	2.00	4.00	7.00	3.00	5.00	6.00	1.00
	Wilcoxon	=	+	+	+	+	+	–
	$\:\boldsymbol{p}$-value	5.30E-01	1.92E-06	1.73E-06	1.73E-06	1.49E-05	1.73E-06	–
F20	Best	2.246E + 03	2.214E + 03	2.621E + 03	2.477E + 03	2.409E + 03	2.353E + 03	**2.129E + 03**
	Mean	2.513E + 03	2.531E + 03	2.921E + 03	2.816E + 03	2.753E + 03	2.958E + 03	**2.393E + 03**
	Std	1.498E + 02	1.898E + 02	**1.283E + 02**	2.190E + 02	1.727E + 02	2.768E + 02	1.824E + 02
	Rank	2.00	3.00	6.00	5.00	4.00	7.00	1.00
	Wilcoxon	+	+	+	+	+	+	–
	$\:\boldsymbol{p}$-value	2.30E-02	9.84E-03	1.92E-06	6.34E-06	2.60E-06	2.60E-06	–
F21	Best	2.368E + 03	2.352E + 03	2.571E + 03	2.499E + 03	2.421E + 03	2.526E + 03	**2.344E + 03**
	Mean	2.399E + 03	2.408E + 03	2.604E + 03	2.599E + 03	2.495E + 03	2.638E + 03	**2.374E + 03**
	Std	1.869E + 01	2.811E + 01	2.223E + 01	5.887E + 01	4.140E + 01	5.308E + 01	**1.601E + 01**
	Rank	2.00	3.00	6.00	5.00	4.00	7.00	1.00
	Wilcoxon	+	+	+	+	+	+	–
	$\:\boldsymbol{p}$-value	5.28E-04	3.72E-05	1.73E-06	1.73E-06	1.73E-06	1.73E-06	–
F22	Best	2.320E + 03	2.621E + 03	4.933E + 03	2.572E + 03	3.547E + 03	3.776E + 03	**2.300E + 03**
	Mean	4.342E + 03	5.500E + 03	9.580E + 03	7.435E + 03	6.492E + 03	8.187E + 03	**3.960E + 03**
	Std	1.718E + 03	1.789E + 03	1.860E + 03	1.484E + 03	**9.790E + 02**	1.468E + 03	1.574E + 03
	Rank	2.00	3.00	7.00	5.00	4.00	6.00	1.00
	Wilcoxon	=	+	+	+	+	+	–
	$\:\boldsymbol{p}$-value	5.04E-01	7.51E-05	1.73E-06	5.22E-06	8.47E-06	2.88E-06	–
F23	Best	2.748E + 03	2.741E + 03	2.947E + 03	3.037E + 03	2.767E + 03	2.980E + 03	**2.728E + 03**
	Mean	2.811E + 03	2.796E + 03	3.080E + 03	3.295E + 03	2.838E + 03	3.161E + 03	**2.778E + 03**
	Std	3.716E + 01	4.509E + 01	4.643E + 01	1.454E + 02	**3.631E + 01**	9.957E + 01	3.659E + 01
	Rank	3.00	2.00	5.00	7.00	4.00	6.00	1.00
	Wilcoxon	+	=	+	+	+	+	–
	$\:\boldsymbol{p}$-value	1.29E-03	5.98E-02	1.73E-06	1.73E-06	3.72E-05	1.73E-06	–
F24	Best	2.898E + 03	2.901E + 03	3.181E + 03	3.320E + 03	2.898E + 03	3.056E + 03	**2.890E + 03**
	Mean	2.951E + 03	2.971E + 03	3.256E + 03	3.520E + 03	2.980E + 03	3.281E + 03	**2.927E + 03**
	Std	3.425E + 01	6.266E + 01	3.837E + 01	1.282E + 02	3.990E + 01	1.248E + 02	**2.501E + 01**
	Rank	2.00	3.00	5.00	7.00	4.00	6.00	1.00
	Wilcoxon	+	+	+	+	+	+	–
	$\:\boldsymbol{p}$-value	9.84E-03	1.83E-03	1.73E-06	1.73E-06	6.98E-06	1.73E-06	–
F25	Best	2.892E + 03	2.959E + 03	3.255E + 03	2.947E + 03	2.896E + 03	3.003E + 03	**2.888E + 03**
	Mean	2.941E + 03	3.031E + 03	3.611E + 03	3.017E + 03	3.170E + 03	3.227E + 03	**2.898E + 03**
	Std	2.801E + 01	8.032E + 01	2.571E + 02	2.715E + 01	3.030E + 02	9.264E + 01	**1.262E + 01**
	Rank	2.00	4.00	7.00	3.00	5.00	6.00	1.00
	Wilcoxon	+	+	+	+	+	+	–
	$\:\boldsymbol{p}$-value	3.52E-06	1.73E-06	1.73E-06	1.73E-06	2.35E-06	1.73E-06	–
F26	Best	4.866E + 03	**4.148E + 03**	5.698E + 03	4.247E + 03	4.756E + 03	5.003E + 03	4.344E + 03
	Mean	5.692E + 03	**4.996E + 03**	7.788E + 03	7.997E + 03	5.946E + 03	8.510E + 03	5.226E + 03
	Std	4.858E + 02	5.637E + 02	6.175E + 02	1.215E + 03	5.791E + 02	1.561E + 03	**3.909E + 02**
	Rank	3.00	1.00	5.00	6.00	4.00	7.00	2.00
	Wilcoxon	+	=	+	+	+	+	–
	$\:\boldsymbol{p}$-value	3.06E-04	7.52E-02	1.73E-06	2.60E-06	1.24E-05	2.13E-06	–
F27	Best	3.255E + 03	3.222E + 03	3.409E + 03	3.362E + 03	**3.205E + 03**	3.300E + 03	3.221E + 03
	Mean	3.297E + 03	3.271E + 03	3.562E + 03	3.628E + 03	3.251E + 03	3.515E + 03	**3.248E + 03**
	Std	3.455E + 01	2.967E + 01	9.490E + 01	1.954E + 02	2.852E + 01	1.667E + 02	**1.961E + 01**
	Rank	4.00	3.00	6.00	7.00	2.00	5.00	1.00
	Wilcoxon	+	=	+	+	=	+	–
	$\:\boldsymbol{p}$-value	5.21E-06	1.65E-01	1.73E-06	1.73E-06	5.45E-02	1.73E-06	–
F28	Best	3.273E + 03	3.322E + 03	4.062E + 03	3.336E + 03	3.361E + 03	3.474E + 03	**3.210E + 03**
	Mean	3.373E + 03	3.465E + 03	4.448E + 03	3.519E + 03	4.245E + 03	3.876E + 03	**3.265E + 03**
	Std	5.398E + 01	8.929E + 01	3.324E + 02	1.134E + 02	9.547E + 02	2.768E + 02	**3.628E + 01**
	Rank	2.00	3.00	7.00	4.00	6.00	5.00	1.00
	Wilcoxon	+	+	+	+	+	+	–
	$\:\boldsymbol{p}$-value	1.92E-06	1.73E-06	1.73E-06	1.73E-06	1.73E-06	1.73E-06	–
F29	Best	**3.473E + 03**	3.672E + 03	4.783E + 03	4.222E + 03	3.663E + 03	4.241E + 03	3.641E + 03
	Mean	4.085E + 03	3.935E + 03	5.224E + 03	5.089E + 03	4.044E + 03	5.402E + 03	**3.920E + 03**
	Std	2.370E + 02	**1.735E + 02**	2.531E + 02	5.381E + 02	2.418E + 02	7.368E + 02	1.860E + 02
	Rank	4.00	2.00	6.00	5.00	3.00	7.00	1.00
	Wilcoxon	+	=	+	+	=	+	–
	$\:\boldsymbol{p}$-value	5.32E-03	8.61E-01	1.73E-06	1.92E-06	5.98E-02	1.73E-06	–
F30	Best	9.596E + 03	8.910E + 05	7.064E + 07	7.866E + 05	1.955E + 04	2.143E + 07	**5.408E + 03**
	Mean	2.232E + 05	1.240E + 07	2.069E + 08	1.342E + 07	5.498E + 05	8.550E + 07	**4.538E + 04**
	Std	1.996E + 05	9.471E + 06	7.863E + 07	2.086E + 07	1.101E + 06	5.113E + 07	**5.149E + 04**
	Rank	2.00	4.00	7.00	5.00	3.00	6.00	1.00
	Wilcoxon	+	+	+	+	+	+	–
	$\:\boldsymbol{p}$-value	1.24E-05	1.73E-06	1.73E-06	1.73E-06	2.22E-04	1.73E-06	–
Wilcoxon Win/Tie/Loss		25/4/0	22/7/0	29/0/0	28/1/0	27/2/0	29/0/0	0/0/0
Avg Rank		2.31	3.14	6.17	4.72	4.38	6.17	**1.10**
Friedman rank		2	3	6.5	5	4	6.5	1
Friedman$\:\boldsymbol{p}$-value		* 4.873E-26*						
Friedman$\:{\boldsymbol{\chi\:}}^{2}$		132.0000						

#### DASO vs other algorithms on CEC2017 with a dimension of 50

**Fig. 6:**
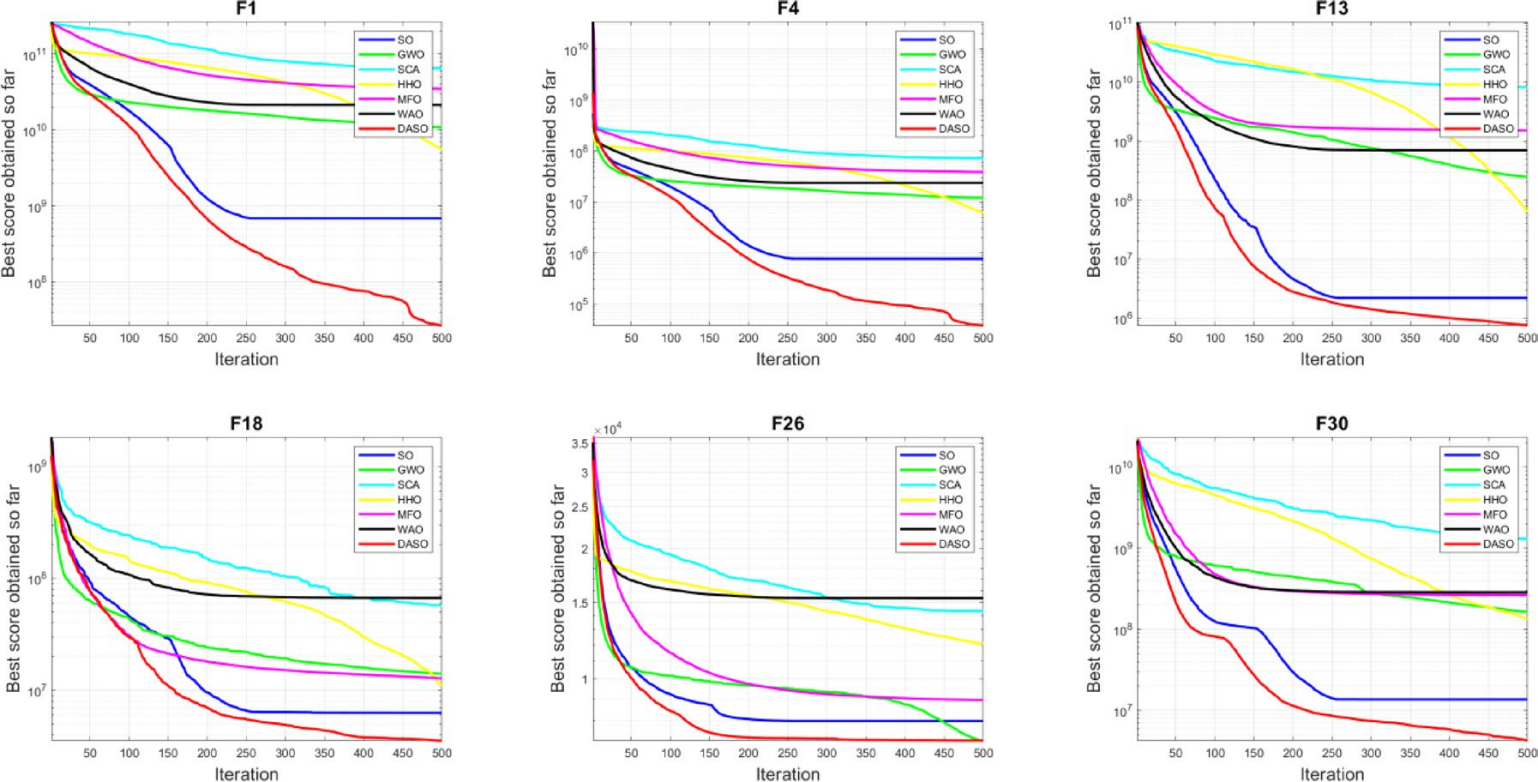
The samples of convergence curves for DASO vs. other algorithms on CEC2017 with Dim = 50.

Experimental results of DASO against other algorithms on CEC2017 with 50 dimension are displayed in Table 7, which shows a summary of the optimal objective function, including its optimum value, mean, standard deviation, and the outcomes of the Wilcoxon and Friedman tests. A sample of the generated convergence curves for DASO against the compared algorithms are displayed in Fig. 6. From this figure, the proposed DASO evidently converges more rapidly to a point than the other algorithms employed. As noted from the previous two experiments and also these experiments, DASO’s performance improved when the dimensions increased.

Table 7 presents that the average values calculated by DASO are significantly better than those calculated using the competing techniques in this study. The objective functions mean values for DASO ranked first over all the other method in all of the benchmark functions (26 out of 29 functions), except for F10 where DASO ranked second, and in F3 and F22 where it ranked third. DASO achieved an average rank of 1.17, which makes its overall performance superior compared with the other considered algorithms in higher dimensions. The Wilcoxon’s rank sum test counts in Table 7 indicate that DASO is statistically significant.

**Table 7:** Comparison of results between DASO and other algorithms on CEC2017, Dim = 50.

F(x)		SO	GWO	SCA	HHO	MFO	WOA	DASO
F1	Best	2.348E + 08	2.665E + 09	5.154E + 10	3.548E + 09	5.573E + 09	1.157E + 10	**5.190E + 05**
	Mean	1.026E + 07	1.093E + 10	6.567E + 10	5.623E + 09	3.489E + 10	2.145E + 10	**1.577E + 06**
	Std	3.921E + 08	4.178E + 09	7.776E + 09	9.735E + 08	2.183E + 10	5.012E + 09	**3.827E + 07**
	Rank	2.00	4.00	7.00	3.00	6.00	5.00	1.00
	Wilcoxon	+	+	+	+	+	+	–
	$\:\boldsymbol{p}$-value	1.47E-04	1.73E-06	1.73E-06	1.73E-06	1.73E-06	1.73E-06	–
F3	Best	1.259E + 05	**1.072E + 05**	1.371E + 05	1.270E + 05	2.702E + 05	1.645E + 05	1.523E + 05
	Mean	**1.642E + 05**	1.698E + 05	2.128E + 05	1.715E + 05	4.358E + 05	3.034E + 05	1.704E + 05
	Std	**1.730E + 04**	3.496E + 04	4.843E + 04	2.253E + 04	1.013E + 05	9.662E + 04	4.641E + 04
	Rank	1.00	2.00	5.00	4.00	7.00	6.00	3.00
	Wilcoxon	-	-	=	=	+	+	–
	$\:\boldsymbol{p}$-value	1.04E-02	7.51E-05	1.99E-01	2.28E-01	1.73E-06	1.59E-03	–
F4	Best	6.910E + 02	9.334E + 02	8.933E + 03	1.234E + 03	1.898E + 03	3.009E + 03	**5.464E + 02**
	Mean	6.654E + 02	1.609E + 03	1.320E + 04	1.741E + 03	5.105E + 03	5.143E + 03	**6.110E + 02**
	Std	1.321E + 02	7.169E + 02	2.793E + 03	5.177E + 02	2.452E + 03	1.317E + 03	**5.721E + 01**
	Rank	2.00	3.00	7.00	4.00	5.00	6.00	1.00
	Wilcoxon	+	+	+	+	+	+	–
	$\:\boldsymbol{p}$-value	2.41E-03	1.73E-06	1.73E-06	1.73E-06	1.73E-06	1.73E-06	–
F5	Best	6.416E + 02	6.668E + 02	1.024E + 03	8.647E + 02	7.076E + 02	9.704E + 02	**6.366E + 02**
	Mean	7.079E + 02	7.581E + 02	1.134E + 03	9.424E + 02	9.640E + 02	1.111E + 03	**6.660E + 02**
	Std	3.455E + 01	5.581E + 01	4.289E + 01	3.523E + 01	1.074E + 02	7.575E + 01	**2.506E + 01**
	Rank	2.00	3.00	7.00	4.00	5.00	6.00	1.00
	Wilcoxon	+	+	+	+	+	+	–
	$\:\boldsymbol{p}$-value	3.40E-05	4.29E-06	1.73E-06	1.73E-06	1.92E-06	1.73E-06	–
F6	Best	6.190E + 02	6.128E + 02	6.752E + 02	6.679E + 02	6.438E + 02	6.810E + 02	**6.091E + 02**
	Mean	6.280E + 02	6.247E + 02	6.850E + 02	6.808E + 02	6.628E + 02	7.002E + 02	**6.145E + 02**
	Std	5.579E + 00	5.622E + 00	6.098E + 00	5.637E + 00	8.929E + 00	1.219E + 01	**5.224E + 00**
	Rank	3.00	2.00	6.00	5.00	4.00	7.00	1.00
	Wilcoxon	+	+	+	+	+	+	–
	$\:\boldsymbol{p}$-value	1.73E-06	2.22E-04	1.73E-06	1.73E-06	1.73E-06	1.73E-06	–
F7	Best	1.032E + 03	1.006E + 03	1.707E + 03	1.722E + 03	1.317E + 03	1.650E + 03	**9.215E + 02**
	Mean	1.067E + 03	1.141E + 03	1.870E + 03	1.889E + 03	1.959E + 03	1.915E + 03	**9.681E + 02**
	Std	7.103E + 01	9.211E + 01	9.428E + 01	9.278E + 01	4.202E + 02	1.131E + 02	**6.955E + 01**
	Rank	2.00	3.00	4.00	5.00	7.00	6.00	1.00
	Wilcoxon	+	+	+	+	+	+	–
	$\:\boldsymbol{p}$-value	4.72E-06	1.60E-04	1.73E-06	1.73E-06	1.73E-06	1.73E-06	–
F8	Best	9.722E + 02	9.588E + 02	1.391E + 03	1.184E + 03	1.075E + 03	1.269E + 03	**9.567E + 02**
	Mean	1.016E + 03	1.078E + 03	1.455E + 03	1.250E + 03	1.277E + 03	1.403E + 03	**9.724E + 02**
	Std	**3.095E + 01**	6.297E + 01	3.447E + 01	3.609E + 01	8.895E + 01	7.791E + 01	3.356E + 01
	Rank	2.00	3.00	7.00	4.00	5.00	6.00	1.00
	Wilcoxon	+	+	+	+	+	+	–
	$\:\boldsymbol{p}$-value	6.32E-05	3.72E-05	1.73E-06	1.73E-06	1.73E-06	1.73E-06	–
F9	Best	3.391E + 03	4.762E + 03	2.103E + 04	2.637E + 04	1.399E + 04	2.468E + 04	**1.947E + 03**
	Mean	5.061E + 03	1.160E + 04	3.203E + 04	3.138E + 04	1.970E + 04	3.698E + 04	**2.833E + 03**
	Std	2.401E + 03	5.404E + 03	4.480E + 03	2.911E + 03	4.407E + 03	9.841E + 03	**1.497E + 03**
	Rank	2.00	3.00	6.00	5.00	4.00	7.00	1.00
	Wilcoxon	+	+	+	+	+	+	–
	$\:\boldsymbol{p}$-value	3.88E-06	1.92E-06	1.73E-06	1.73E-06	1.73E-06	1.73E-06	–
F10	Best	6.376E + 03	6.038E + 03	1.472E + 04	8.013E + 03	7.691E + 03	1.236E + 04	**5.962E + 03**
	Mean	9.624E + 03	**8.275E + 03**	1.552E + 04	1.033E + 04	9.492E + 03	1.323E + 04	9.469E + 03
	Std	2.829E + 03	2.132E + 03	**4.302E + 02**	1.074E + 03	9.349E + 02	8.053E + 02	2.116E + 03
	Rank	4.00	1.00	7.00	5.00	3.00	6.00	2.00
	Wilcoxon	=	-	+	=	=	+	–
	$\:\boldsymbol{p}$-value	8.61E-01	1.11E-02	1.73E-06	7.19E-02	8.61E-01	2.60E-06	–
F11	Best	1.802E + 03	3.493E + 03	8.655E + 03	2.126E + 03	2.909E + 03	4.697E + 03	**1.396E + 03**
	Mean	1.830E + 03	8.052E + 03	1.282E + 04	3.192E + 03	1.491E + 04	8.644E + 03	**1.454E + 03**
	Std	1.126E + 03	3.303E + 03	2.653E + 03	7.237E + 02	9.879E + 03	2.710E + 03	**4.850E + 02**
	Rank	2.00	4.00	6.00	3.00	7.00	5.00	1.00
	Wilcoxon	+	+	+	+	+	+	–
	$\:\boldsymbol{p}$-value	1.92E-06	1.73E-06	1.73E-06	3.52E-06	1.92E-06	1.73E-06	–
F12	Best	2.165E + 07	1.430E + 08	1.543E + 10	2.836E + 08	1.688E + 08	1.947E + 09	**1.614E + 06**
	Mean	2.779E + 07	1.692E + 09	2.298E + 10	9.348E + 08	4.899E + 09	4.736E + 09	**1.258E + 07**
	Std	3.443E + 07	1.555E + 09	5.578E + 09	4.713E + 08	3.443E + 09	1.958E + 09	**2.284E + 07**
	Rank	2.00	4.00	7.00	3.00	6.00	5.00	1.00
	Wilcoxon	+	+	+	+	+	+	–
	$\:\boldsymbol{p}$-value	3.38E-03	1.73E-06	1.73E-06	1.73E-06	1.73E-06	1.73E-06	–
F13	Best	4.321E + 04	2.042E + 06	3.175E + 09	6.574E + 06	7.339E + 04	8.761E + 07	**3.763E + 03**
	Mean	1.025E + 05	1.933E + 08	7.513E + 09	3.137E + 07	1.353E + 09	5.416E + 08	**6.010E + 04**
	Std	3.309E + 05	1.621E + 08	3.171E + 09	2.370E + 07	1.839E + 09	3.417E + 08	**1.584E + 05**
	Rank	2.00	4.00	7.00	3.00	6.00	5.00	1.00
	Wilcoxon	+	+	+	+	+	+	–
	$\:\boldsymbol{p}$-value	7.73E-03	1.73E-06	1.73E-06	1.73E-06	4.73E-06	1.73E-06	–
F14	Best	1.011E + 05	1.593E + 05	2.021E + 06	9.352E + 05	3.764E + 05	7.367E + 05	**3.277E + 04**
	Mean	2.809E + 05	1.634E + 06	8.336E + 06	7.871E + 06	2.603E + 06	8.183E + 06	**1.788E + 05**
	Std	4.701E + 05	1.460E + 06	3.818E + 06	8.928E + 06	2.702E + 06	6.343E + 06	**3.358E + 05**
	Rank	2.00	3.00	7.00	5.00	4.00	6.00	1.00
	Wilcoxon	+	+	+	+	+	+	–
	$\:\boldsymbol{p}$-value	2.30E-02	4.07E-05	1.73E-06	1.73E-06	2.37E-05	1.73E-06	–
F15	Best	9.167E + 03	5.418E + 04	4.265E + 08	6.097E + 05	7.319E + 03	5.975E + 06	**1.951E + 03**
	Mean	4.009E + 04	4.443E + 07	1.143E + 09	3.680E + 06	7.598E + 07	6.809E + 07	**2.227E + 04**
	Std	2.477E + 04	1.189E + 08	4.238E + 08	5.982E + 06	2.936E + 08	7.634E + 07	**1.529E + 04**
	Rank	2.00	4.00	7.00	3.00	6.00	5.00	1.00
	Wilcoxon	+	+	+	+	+	+	–
	$\:\boldsymbol{p}$-value	1.17E-02	1.92E-06	1.73E-06	1.73E-06	1.97E-05	1.73E-06	–
F16	Best	2.747E + 03	2.602E + 03	5.890E + 03	3.800E + 03	3.123E + 03	4.338E + 03	**2.595E + 03**
	Mean	3.432E + 03	3.413E + 03	6.354E + 03	4.929E + 03	4.342E + 03	6.492E + 03	**3.072E + 03**
	Std	4.099E + 02	4.566E + 02	3.001E + 02	6.722E + 02	7.158E + 02	9.325E + 02	**2.958E + 02**
	Rank	3.00	2.00	6.00	5.00	4.00	7.00	1.00
	Wilcoxon	+	=	+	+	+	+	–
	$\:\boldsymbol{p}$-value	2.27E-02	8.59E-02	1.73E-06	1.73E-06	2.35E-06	1.73E-06	–
F17	Best	2.585E + 03	**2.552E + 03**	4.130E + 03	3.086E + 03	2.968E + 03	3.673E + 03	2.624E + 03
	Mean	3.127E + 03	3.125E + 03	5.194E + 03	3.901E + 03	3.964E + 03	4.486E + 03	**2.985E + 03**
	Std	2.987E + 02	**2.859E + 02**	4.411E + 02	4.162E + 02	6.071E + 02	4.539E + 02	2.923E + 02
	Rank	3.00	2.00	7.00	4.00	5.00	6.00	1.00
	Wilcoxon	+	=	+	+	+	+	–
	$\:\boldsymbol{p}$-value	2.56E-02	9.92E-01	1.73E-06	3.18E-06	5.22E-06	1.73E-06	–
F18	Best	1.210E + 06	5.690E + 05	1.866E + 07	1.529E + 06	9.046E + 05	2.825E + 06	**1.657E + 05**
	Mean	6.284E + 06	1.404E + 07	5.755E + 07	1.104E + 07	1.272E + 07	6.673E + 07	**3.543E + 06**
	Std	4.397E + 06	1.397E + 07	2.713E + 07	1.049E + 07	1.363E + 07	4.641E + 07	**2.854E + 06**
	Rank	2.00	5.00	6.00	3.00	4.00	7.00	1.00
	Wilcoxon	+	+	+	+	+	+	–
	$\:\boldsymbol{p}$-value	4.68E-03	1.25E-04	1.73E-06	8.92E-05	1.15E-04	2.35E-06	–
F19	Best	**2.338E + 03**	2.393E + 05	1.822E + 08	2.444E + 05	3.597E + 03	2.122E + 06	3.292E + 03
	Mean	5.312E + 04	1.941E + 07	6.529E + 08	1.699E + 06	5.033E + 07	2.160E + 07	**2.959E + 04**
	Std	**1.133E + 05**	5.054E + 07	3.036E + 08	1.329E + 06	2.280E + 08	2.379E + 07	1.353E + 05
	Rank	2.00	4.00	7.00	3.00	6.00	5.00	1.00
	Wilcoxon	+	+	+	+	+	+	–
	$\:\boldsymbol{p}$-value	2.41E-03	1.73E-06	1.73E-06	1.73E-06	1.48E-04	1.73E-06	–
F20	Best	**2.458E + 03**	2.521E + 03	3.933E + 03	3.093E + 03	2.819E + 03	3.034E + 03	2.592E + 03
	Mean	3.061E + 03	3.148E + 03	4.314E + 03	3.692E + 03	3.590E + 03	3.892E + 03	**2.947E + 03**
	Std	3.274E + 02	4.012E + 02	**1.896E + 02**	3.284E + 02	3.407E + 02	3.821E + 02	3.590E + 02
	Rank	2.00	3.00	7.00	5.00	4.00	6.00	1.00
	Wilcoxon	=	=	+	+	+	+	–
	$\:\boldsymbol{p}$-value	4.16E-01	1.85E-01	1.73E-06	1.06E-04	5.29E-04	5.22E-06	–
F21	Best	2.463E + 03	2.469E + 03	2.853E + 03	2.768E + 03	2.577E + 03	2.915E + 03	**2.445E + 03**
	Mean	2.513E + 03	2.557E + 03	2.961E + 03	2.926E + 03	2.740E + 03	3.095E + 03	**2.475 + 03**
	Std	3.568E + 01	3.973E + 01	4.768E + 01	8.746E + 01	7.912E + 01	1.021E + 02	**3.186E + 01**
	Rank	2.00	3.00	6.00	5.00	4.00	7.00	1.00
	Wilcoxon	+	+	+	+	+	+	–
	$\:\boldsymbol{p}$-value	1.74E-04	1.06E-04	1.73E-06	1.73E-06	1.73E-06	1.73E-06	–
F22	Best	**7.184E + 03**	8.130E + 03	1.605E + 04	1.029E + 04	8.238E + 03	1.273E + 04	8.096E + 03
	Mean	1.139E + 04	**1.045E + 04**	1.706E + 04	1.229E + 04	1.056E + 04	1.477E + 04	1.081E + 04
	Std	2.557E + 03	2.268E + 03	**4.470E + 02**	1.027E + 03	1.120E + 03	1.156E + 03	1.846E + 03
	Rank	4.00	1.00	7.00	5.00	2.00	6.00	3.00
	Wilcoxon	=	=	+	+	=	+	–
	$\:\boldsymbol{p}$-value	4.17E-01	2.29E-01	1.73E-06	1.96E-03	9.59E-01	1.92E-06	–
F23	Best	2.920E + 03	**2.910E + 03**	3.586E + 03	3.645E + 03	3.036E + 03	3.432E + 03	2.942E + 03
	Mean	3.085E + 03	3.055E + 03	3.741E + 03	4.052E + 03	3.196E + 03	3.838E + 03	**3.021E + 03**
	Std	7.154E + 01	8.707E + 01	7.691E + 01	2.530E + 02	7.937E + 01	2.000E + 02	**6.655E + 01**
	Rank	3.00	2.00	5.00	7.00	4.00	6.00	1.00
	Wilcoxon	+	=	+	+	+	+	–
	$\:\boldsymbol{p}$-value	9.63E-04	7.66E-01	1.73E-06	1.73E-06	6.98E-06	1.73E-06	–
F24	Best	3.145E + 03	3.118E + 03	3.720E + 03	3.980E + 03	3.122E + 03	3.650E + 03	**3.055E + 03**
	Mean	3.219E + 03	3.216E + 03	3.877E + 03	4.415E + 03	3.244E + 03	3.918E + 03	**3.145E + 03**
	Std	5.128E + 01	8.720E + 01	9.280E + 01	2.596E + 02	5.449E + 01	1.773E + 02	**4.979E + 01**
	Rank	3.00	2.00	5.00	7.00	4.00	6.00	1.00
	Wilcoxon	+	=	+	+	+	+	–
	$\:\boldsymbol{p}$-value	4.45E-05	6.27E-02	1.73E-06	1.73E-06	8.92E-05	1.73E-06	–
F25	Best	3.170E + 03	3.443E + 03	7.295E + 03	3.398E + 03	3.320E + 03	4.628E + 03	**3.062E + 03**
	Mean	3.338E + 03	3.932E + 03	9.304E + 03	3.805E + 03	5.552E + 03	5.283E + 03	**3.111E + 03**
	Std	1.388E + 02	4.186E + 02	1.068E + 03	2.339E + 02	1.835E + 03	6.296E + 02	**3.306E + 01**
	Rank	2.00	4.00	7.00	3.00	6.00	5.00	1.00
	Wilcoxon	+	+	+	+	+	+	–
	$\:\boldsymbol{p}$-value	1.73E-06	1.73E-06	1.73E-06	1.73E-06	1.73E-06	1.73E-06	–
F26	Best	6.188E + 03	6.083E + 03	1.241E + 04	7.676E + 03	7.237E + 03	1.178E + 04	**5.768E + 03**
	Mean	7.633E + 03	7.073E + 03	1.405E + 04	1.192E + 04	8.756E + 03	1.521E + 04	**6.785E + 03**
	Std	9.095E + 02	7.909E + 02	9.009E + 02	1.339E + 03	7.566E + 02	1.434E + 03	**6.058E + 02**
	Rank	3.00	2.00	6.00	5.00	4.00	7.00	1.00
	Wilcoxon	+	=	+	+	+	+	–
	$\:\boldsymbol{p}$-value	1.64E-05	7.66E-01	1.73E-06	1.73E-06	2.60E-06	1.73E-06	–
F27	Best	3.609E + 03	3.504E + 03	4.609E + 03	4.146E + 03	3.429E + 03	3.968E + 03	**3.400E + 03**
	Mean	3.798E + 03	3.756E + 03	5.028E + 03	4.969E + 03	3.645E + 03	4.892E + 03	**3.641E + 03**
	Std	**1.091E + 02**	1.323E + 02	1.932E + 02	7.008E + 02	1.319E + 02	6.182E + 02	1.289E + 02
	Rank	4.00	3.00	7.00	6.00	2.00	5.00	1.00
	Wilcoxon	+	+	+	+	=	+	–
	$\:\boldsymbol{p}$-value	4.72E-06	2.30E-02	1.73E-06	1.73E-06	3.09E-01	1.73E-06	–
F28	Best	3.714E + 03	3.835E + 03	6.597E + 03	3.791E + 03	3.672E + 03	5.246E + 03	**3.340E + 03**
	Mean	4.243E + 03	4.672E + 03	8.906E + 03	4.916E + 03	8.444E + 03	6.344E + 03	**3.427E + 03**
	Std	4.222E + 02	4.121E + 02	9.639E + 02	4.724E + 02	1.119E + 03	5.880E + 02	**7.143E + 01**
	Rank	2.00	3.00	7.00	4.00	6.00	5.00	1.00
	Wilcoxon	+	+	+	+	+	+	–
	$\:\boldsymbol{p}$-value	1.73E-06	1.73E-06	1.73E-06	1.73E-06	1.73E-06	1.73E-06	–
F29	Best	**4.263E + 03**	4.280E + 03	7.414E + 03	5.560E + 03	4.491E + 03	7.615E + 03	4.274E + 03
	Mean	5.021E + 03	5.124E + 03	9.115E + 03	7.444E + 03	5.397E + 03	9.777E + 03	**4.674E + 03**
	Std	4.078E + 02	4.537E + 02	1.008E + 03	1.228E + 03	5.010E + 02	1.773E + 03	**3.178E + 02**
	Rank	2.00	3.00	6.00	5.00	4.00	7.00	1.00
	Wilcoxon	+	+	+	+	+	+	–
	$\:\boldsymbol{p}$-value	1.11E-03	4.95E-02	1.73E-06	1.73E-06	1.29E-03	1.73E-06	–
F30	Best	3.102E + 06	8.504E + 07	6.661E + 08	3.502E + 07	3.515E + 06	1.668E + 08	**8.498E + 05**
	Mean	1.366E + 07	1.647E + 08	1.314E + 09	1.348E + 08	2.660E + 08	2.870E + 08	**4.240E + 06**
	Std	6.271E + 06	5.712E + 07	3.963E + 08	7.136E + 07	7.255E + 08	9.291E + 07	**3.713E + 06**
	Rank	2.00	4.00	7.00	3.00	5.00	6.00	1.00
	Wilcoxon	+	+	+	+	+	+	–
	$\:\boldsymbol{p}$-value	6.98E-06	1.73E-06	1.73E-06	1.73E-06	1.49E-05	1.73E-06	–
Wilcoxon Win/Tie/Loss		25/3/1	20/7/2	28/1/0	27/1/1	26/3/0	29/0/0	0/0/0
Avg Rank		2.37	2.96	6.41	4.34	4.79	5.93	**1.17**
Friedman rank		2	3	7	4	5	6	1
Friedman$\:\boldsymbol{p}$-value		* 2.551E-25*						
Friedman$\:{\boldsymbol{\chi\:}}^{2}$		128.5862						

### CEC 2020 benchmarks

Algorithms intended for optimizing a singular objective challenge serve as the core components from which more complicated techniques, such as multi-objective, niching, and constrained optimization, are derived. Consequently, enhancing single-objective optimization methods is essential, as it may impact various other areas. Experiments utilizing single-objective benchmark functions are essential for the iterative improvement of these algorithms. The CEC’20 Special Session has been developed in Real Parameter Optimization to strengthen the symbiotic relationship between methodologies and challenges, hence facilitating improvement^65^. F1–F10 possesses identical dimensions to a 10-dimensional bound optimization problem within the [−100, 100] boundary range.

**Fig. 7:**
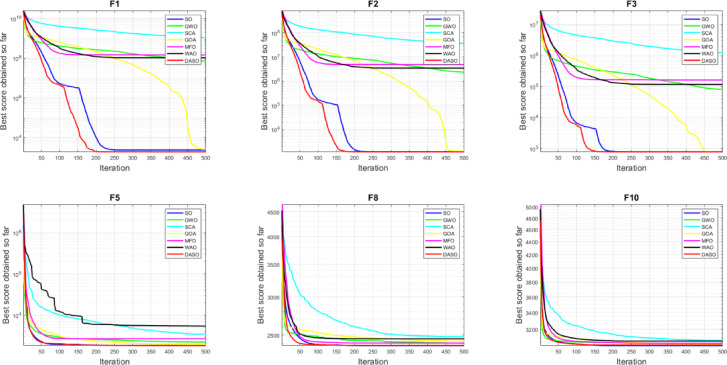
The samples of convergence curves for DASO vs. other algorithms on CEC2020.

Our comparison of DASO to the algorithms considered in this study using CEC2020 functions is demonstrated in Table 8, which provides a summary of the optimal objective function, including its optimum value, mean, standard deviation, and the outcomes of the Wilcoxon and Friedman tests. The results in Table 8 indicate that DASO has an overall average rank of 1.00, which makes it ranked as first in comparison to the other algorithms on each function. The Wilcoxon test results indicate a statistically significant superiority to the competing algorithms considered in this study. Figure 7. presents a sample of the convergence curves of the compared algorithms. From the experimental results, it was found that the suggested DASO algorithm demonstrated superior performance relative to the original SO technique and all other competing techniques considered in this comparison.

**Table 8:** Comparison of results between DASO and other algorithms on CEC2020.

F(x)		SO	GWO	SCA	HHO	MFO	WOA	DASO
F1	Best	1.127E + 02	3.393E + 04	4.756E + 08	2.431E + 05	**1.023E + 02**	4.071E + 06	1.043E + 02
	Mean	3.285E + 03	2.828E + 07	1.020E + 09	2.105E + 06	1.543E + 08	1.376E + 08	**2.818E + 03**
	Std	3.250E + 03	8.898E + 07	3.246E + 08	4.447E + 06	4.648E + 08	1.983E + 08	**2.921E + 03**
	Rank	3.00	4.00	7.00	3.00	6.00	5.00	1.00
	Wilcoxon	=	+	+	+	+	+	=
	$\:\boldsymbol{p}$-value	7.19E-01	1.73E-06	1.73E-06	1.73E-06	1.29E-03	1.73E-06	–
F2	Best	1.104E + 03	1.116E + 03	1.161E + 03	1.114E + 03	1.106E + 03	1.131E + 03	**1.101E + 03**
	Mean	1.124E + 03	1.159E + 03	1.259E + 03	1.173E + 03	1.455E + 03	1.237E + 03	**1.114E + 03**
	Std	2.387E + 01	6.093E + 01	6.759E + 01	4.763E + 01	7.271E + 02	6.845E + 01	**1.278E + 01**
	Rank	2.00	3.00	6.00	4.00	7.00	5.00	1.00
	Wilcoxon	+	+	+	+	+	+	=
	$\:\boldsymbol{p}$-value	3.00E-02	1.36E-05	1.73E-06	2.88E-06	9.32E-06	2.35E-06	–
F3	Best	**7.155E + 02**	7.175E + 02	7.659E + 02	7.437E + 02	7.201E + 02	7.339E + 02	**7.155E + 02**
	Mean	7.407E + 02	7.340E + 02	7.845E + 02	7.875E + 02	7.367E + 02	7.859E + 02	**7.284E + 02**
	Std	1.142E + 01	1.122E + 01	9.246E + 00	2.103E + 01	1.278E + 01	2.693E + 01	**8.890E + 00**
	Rank	4.00	2.00	5.00	7.00	3.00	6.00	1.00
	Wilcoxon	+	=	+	+	=	+	=
	$\:\boldsymbol{p}$-value	3.16E-03	3.60E-01	1.73E-06	1.73E-06	2.21E-01	2.13E-06	–
F4	Best	1.918E + 03	1.922E + 03	2.080E + 03	2.518E + 03	1.953E + 03	2.839E + 03	**1.909E + 03**
	Mean	2.615E + 03	1.537E + 04	1.064E + 04	1.656E + 04	1.799E + 04	8.783E + 04	**1.983E + 03**
	Std	2.244E + 03	3.163E + 04	9.111E + 03	1.315E + 04	2.065E + 04	1.216E + 05	**3.020E + 02**
	Rank	2.00	4.00	3.00	5.00	6.00	7.00	1.00
	Wilcoxon	+	+	+	+	+	+	=
	$\:\boldsymbol{p}$-value	4.77E-03	8.47E-06	3.88E-06	1.73E-06	3.18E-06	1.73E-06	–
F5	Best	1.720E + 03	1.727E + 03	1.762E + 03	1.744E + 03	1.723E + 03	1.759E + 03	**1.704E + 03**
	Mean	1.760E + 03	1.762E + 03	1.803E + 03	1.786E + 03	1.763E + 03	1.845E + 03	**1.741E + 03**
	Std	4.236E + 01	2.709E + 01	2.778E + 01	5.015E + 01	3.775E + 01	7.591E + 01	**1.985E + 01**
	Rank	2.00	3.00	6.00	5.00	4.00	7.00	1.00
	Wilcoxon	+	+	+	+	+	+	=
	$\:\boldsymbol{p}$-value	8.22E-03	1.20E-03	1.73E-06	2.88E-06	7.73E-03	2.60E-06	–
F6	Best	1.602E + 03	1.619E + 03	1.670E + 03	1.730E + 03	1.639E + 03	1.749E + 03	**1.601E + 03**
	Mean	1.763E + 03	1.757E + 03	1.822E + 03	1.940E + 03	1.781E + 03	1.944E + 03	**1.658E + 03**
	Std	1.238E + 02	1.203E + 02	8.625E + 01	1.073E + 02	9.098E + 01	1.540E + 02	**6.738E + 01**
	Rank	3.00	2.00	5.00	6.00	4.00	7.00	1.00
	Wilcoxon	+	+	+	+	+	+	=
	$\:\boldsymbol{p}$-value	1.89E-04	4.90E-04	9.32E-06	1.73E-06	2.60E-05	2.13E-06	–
F7	Best	2.308E + 03	2.205E + 03	2.211E + 03	2.208E + 03	**2.200E + 03**	2.215E + 03	2.200E + 03
	Mean	2.318E + 03	2.315E + 03	2.298E + 03	2.330E + 03	2.319E + 03	2.333E + 03	**2.296E + 03**
	Std	**5.795E + 00**	2.280E + 01	6.450E + 01	5.668E + 01	4.158E + 01	4.826E + 01	2.895E + 01
	Rank	4.00	3.00	2.00	6.00	5.00	7.00	1.00
	Wilcoxon	+	+	=	+	+	+	=
	$\:\boldsymbol{p}$-value	4.39E-03	3.87E-02	2.07E-02	3.50E-02	6.16E-04	1.25E-02	–
F8	Best	2.261E + 03	**2.200E + 03**	2.286E + 03	2.307E + 03	2.229E + 03	2.310E + 03	2.300E + 03
	Mean	2.302E + 03	2.305E + 03	2.386E + 03	2.315E + 03	2.318E + 03	2.509E + 03	**2.301E + 03**
	Std	7.626E + 00	2.792E + 01	3.597E + 01	4.065E + 00	4.374E + 01	4.816E + 02	**1.090E + 00**
	Rank	2.00	3.00	6.00	4.00	5.00	7.00	1.00
	Wilcoxon	=	+	+	+	+	+	=
	$\:\boldsymbol{p}$-value	7.50E-01	4.53E-04	1.92E-06	1.73E-06	2.26E-03	1.73E-06	–
F9	Best	2.739E + 03	2.728E + 03	2.576E + 03	**2.502E + 03**	2.745E + 03	2.581E + 03	2.736E + 03
	Mean	2.752E + 03	2.748E + 03	2.784E + 03	2.801E + 03	2.764E + 03	2.781E + 03	**2.739E + 03**
	Std	9.420E + 00	1.344E + 01	4.014E + 01	1.303E + 02	9.669E + 00	5.948E + 01	**7.079E + 00**
	Rank	3.00	2.00	6.00	7.00	4.00	5.00	1.00
	Wilcoxon	+	=	+	+	+	+	=
	$\:\boldsymbol{p}$-value	2.58E-03	7.50E-01	3.11E-05	1.17E-02	9.32E-06	3.59E-04	–
F10	Best	2.898E + 03	2.899E + 03	2.944E + 03	**2.616E + 03**	2.898E + 03	2.906E + 03	2.898E + 03
	Mean	2.929E + 03	2.939E + 03	2.981E + 03	2.928E + 03	2.942E + 03	2.963E + 03	**2.920E + 03**
	Std	2.247E + 01	**2.161E + 01**	2.205E + 01	6.580E + 01	4.397E + 01	3.466E + 01	2.346E + 01
	Rank	3.00	4.00	7.00	2.00	5.00	6.00	1.00
	Wilcoxon	=	=	+	+	=	+	=
	$\:\boldsymbol{p}$-value	7.66E-01	1.25E-01	1.73E-06	1.11E-03	8.22E-02	1.74E-04	–
Wilcoxon rank sum		7/3/0	7/3/0	9/1/0	10/0/0	8/2/0	10/0/0	0/0/0
Avg Rank		2.80	3.00	5.30	4.90	4.90	6.20	**1.00**
Friedman rank		2	3	6	4.5	4.5	7	1
Friedman$\:\boldsymbol{p}$-value		* 2.864E-06*						
Friedman$\:{\boldsymbol{\chi\:}}^{2}$		35.9143						

### Exploration–exploitation behavior analysis

To gain further insight into the search behavior of DASO, an exploration–exploitation analysis was carried out using the CEC2017 benchmark function F10 with 30 decision variables. Function F10 was selected because it represents a hybrid optimization problem that combines multiple landscape characteristics within a single search space, making it well suited for analyzing the dynamic behavior of metaheuristic algorithms. Unlike the benchmark comparisons presented earlier, this analysis aims to explain how DASO searches for the optimum rather than simply evaluating its optimization accuracy.

**Fig. 8:**
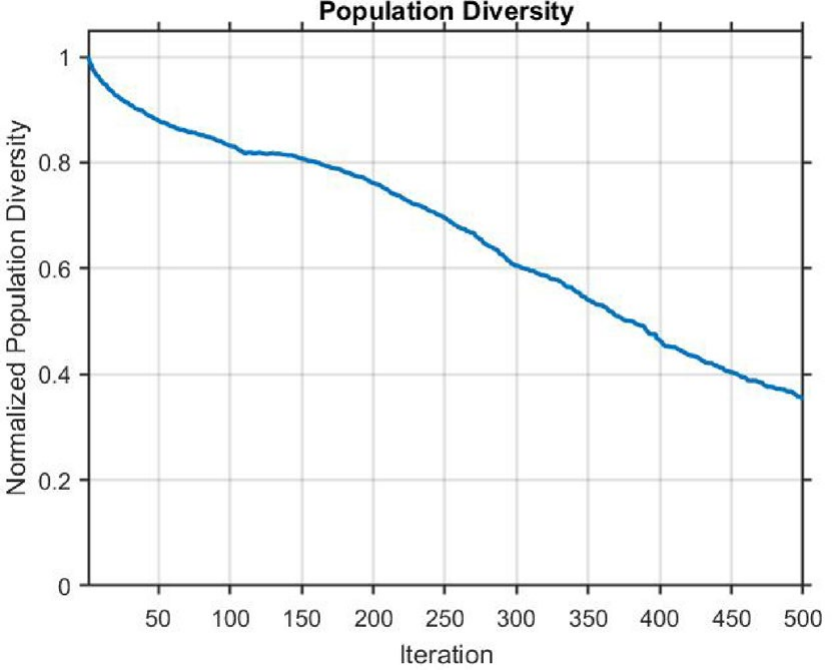
Normalized population diversity.

**Fig. 9:**
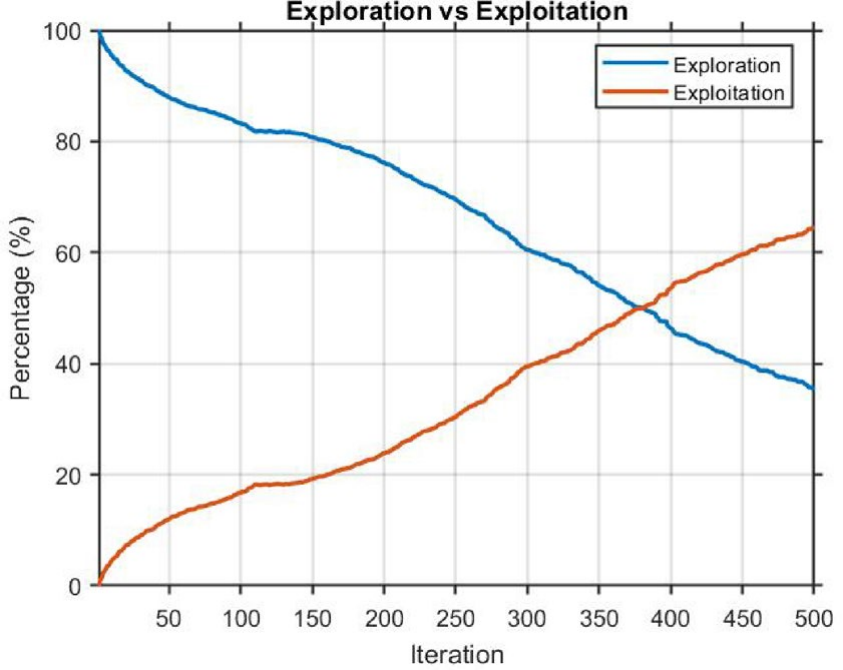
Balance between exploration and exploitation.

**Fig. 10:**
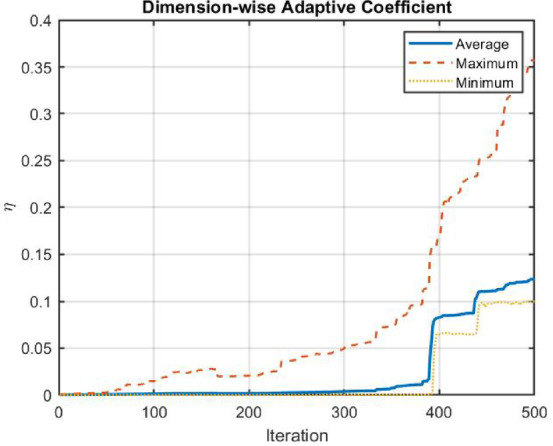
Dimension-wise adaptive step-size coefficient.

Three indicators were monitored during the optimization process: population diversity, the dimension-wise adaptive step-size factor, and the exploration–exploitation ratio. Together, these measures provide a clear picture of how the proposed mechanisms influence the search process. The corresponding results are presented in Figs. 8, 9 and 10

The evolution of the normalized population diversity is presented in Fig. 8. The population begins with a high diversity due to the Sobol sequence initialization, which distributes the search agents more uniformly across the search space. As iterations progress, the diversity decreases steadily, from an initial value close to 1 to approximately 0.36 at the final iteration, rather than dropping abruptly, indicating that the population gradually concentrates around promising regions while still maintaining sufficient variation among individuals. This behavior suggests that DASO preserves exploration during the early stages of the search and avoids the rapid loss of diversity that often leads to premature convergence.

The balance between exploration and exploitation is illustrated in Fig. 9. At the beginning of the optimization, exploration is the dominant search behavior, allowing the algorithm to investigate a wide range of candidate solutions. As the search progresses, the exploration rate decreases steadily while exploitation increases at a similar pace. The two curves intersect near the final quarter of the optimization process, indicating a smooth and adaptive transition from global search to local refinement rather than an abrupt change between the two phases. This smooth transition demonstrates that DASO does not switch suddenly from exploration to exploitation but instead adjusts the search behavior progressively according to the state of the optimization. Such a balanced transition enables the algorithm to continue exploring the search space while increasingly refining high-quality solutions as convergence is approached.

The behavior of the proposed dimension-wise adaptive step-size strategy is presented in Fig. 101010^1^0. During the early iterations, the coefficient remains relatively small because the population is still widely distributed across the search space. As more decision variables begin to converge, the adaptive coefficient increases more noticeably, particularly during the later iterations, while the maximum coefficient also grows as more dimensions approach convergence. This indicates that the adaptive mechanism continuously adjusts the search intensity according to the convergence state of each decision variable. The different trends observed for the average, maximum, and minimum coefficients further demonstrate that each decision variable is updated independently according to its convergence state rather than being controlled by a single global step size. Dimensions that still exhibit high uncertainty continue searching with relatively conservative updates, whereas dimensions that have nearly converged receive stronger local refinement. This adaptive regulation allows different decision variables to evolve at different rates, improving convergence stability and making the search process more effective in high-dimensional optimization problems.

**Fig. 11:**
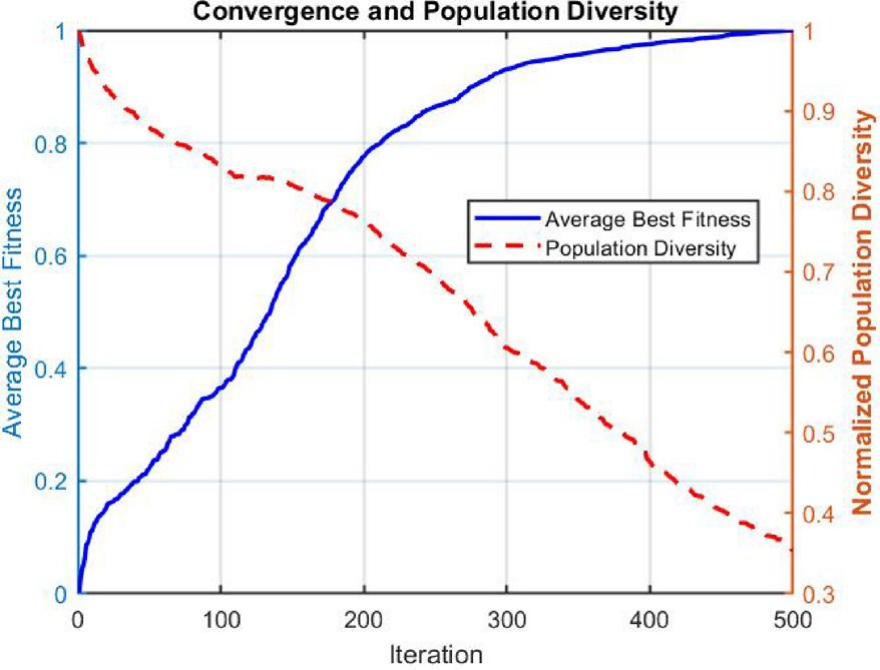
Convergence curve with the normalized population diversity.

Figure 11 combines the convergence curve with the normalized population diversity. As the average best fitness steadily improves, the population diversity decreases progressively rather than collapsing at the beginning of the search. The simultaneous improvement in solution quality and gradual reduction in diversity indicates that DASO maintains sufficient exploration while continuously refining promising regions. This balanced behavior enables the algorithm to continue improving the objective value until the final iterations without showing signs of premature convergence.

Overall, the four analyses provide a clear explanation for the improved performance of DASO. The Sobol initialization creates a diverse starting population, the adaptive threshold ensures a gradual transition between exploration and exploitation, the hybrid Lévy-flight and spiral search strategies strengthen the global and local search capabilities, and the dimension-wise adaptive step-size mechanism adjusts the search intensity according to the convergence state of each decision variable. Working together, these components enable DASO to maintain population diversity when broad exploration is required and progressively focus the search on promising regions as the optimization advances. The observed search behavior is consistent with the superior convergence performance reported in the benchmark experiments, providing further evidence of the effectiveness of the proposed algorithm.

### Ablation study

To assess the contribution of each proposed enhancement, an ablation study was carried out by removing one component from DASO while keeping all other components unchanged. The objective was to evaluate the influence of each strategy on the overall optimization performance and to determine whether the improvements observed in DASO arise from a single enhancement or from the combined effect of the proposed modifications.

Evaluating every ablated variant on all 30 CEC2017 benchmark functions would require a substantial number of additional optimizations runs. To keep the computational cost reasonable while preserving a representative evaluation, eight benchmark functions were selected. Specifically, F1, F4, F5, F8, F9, F10, F17, and F28 with 50 dimensions were chosen because they represent the main categories of the CEC2017 benchmark suite, including unimodal, multimodal, hybrid, and composition functions. These functions exhibit different search characteristics and levels of difficulty, providing a balanced assessment of the proposed enhancement strategies under diverse optimization scenarios.

In addition to the complete DASO algorithm, five ablated variants were evaluated: NoSobol, where Sobol sequence initialization was replaced with random initialization; NoThreshold, where the adaptive exploration–exploitation threshold was removed; NoLevy, which excludes the Lévy-flight exploration strategy; NoSpiral, where the spiral exploitation mechanism was disabled; and NoDASS, in which the proposed dimension-wise adaptive step-size strategy was omitted. All variants were tested using the same experimental settings to ensure a fair comparison.

**Fig. 12:**
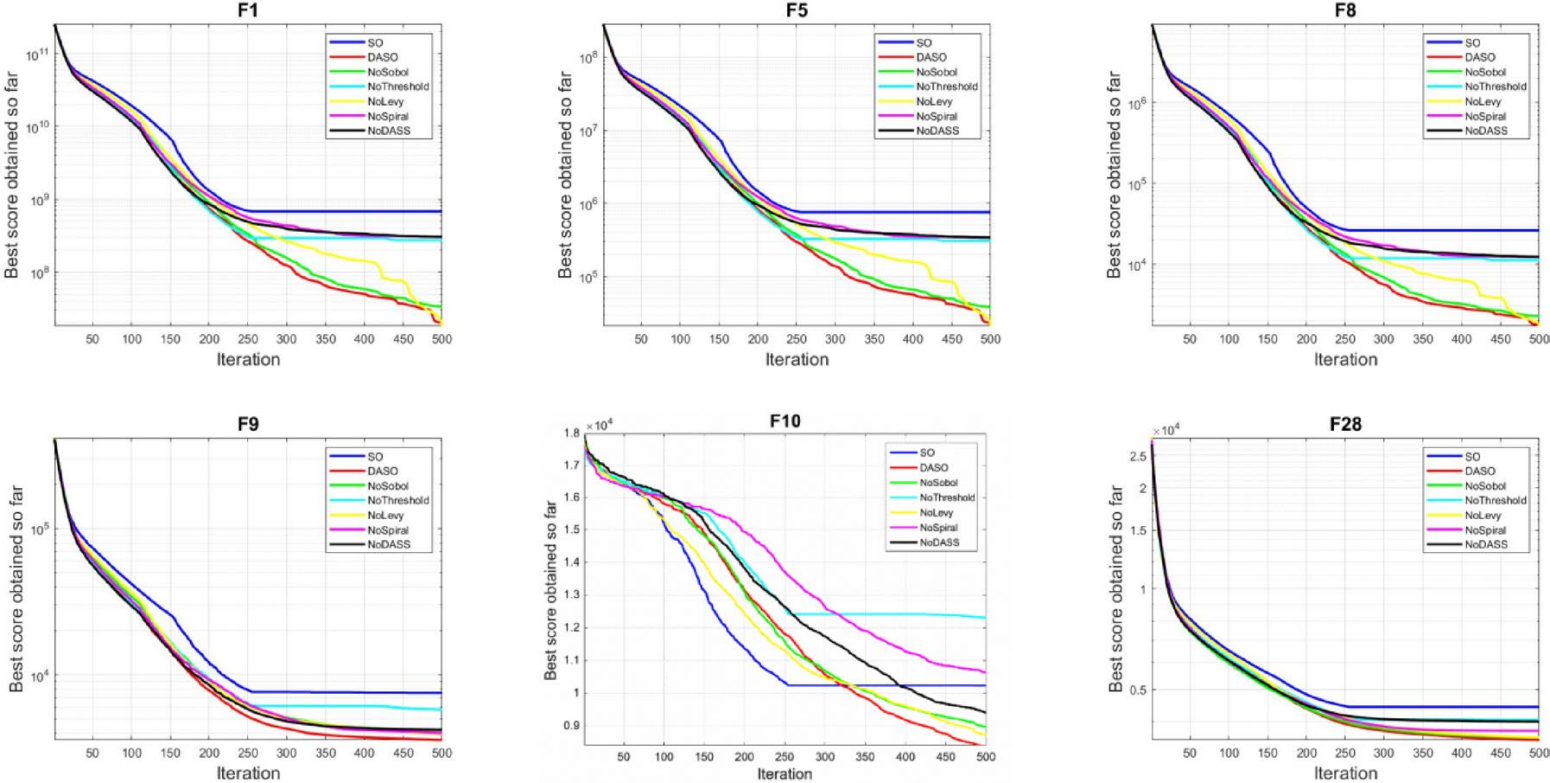
The samples of convergence curves for the ablation study.

The numerical results are reported in Table 9, which presents the mean objective value, standard deviation, performance rank, and relative performance loss for each variant. The final row summarizes the average performance loss across all representative benchmark functions, providing an overall measure of the contribution of each enhancement. The average performance loss was computed using F4–F28. Function F1 was excluded from the average because the near-zero objective value achieved by DASO produces disproportionately large relative percentage values that would dominate the summary statistics. To complement the numerical comparison, the convergence curves of DASO and its ablated variants are shown in Fig. 12, illustrating the influence of each component on the convergence behavior.

The results clearly indicate that the complete DASO algorithm provides the best overall performance across the selected benchmark functions. Removing any of the proposed mechanisms leads to a reduction in optimization performance, confirming that each enhancement plays a meaningful role in the overall search process.

Among all components, the adaptive threshold mechanism has the greatest impact. Eliminating this strategy results in the largest average performance loss (24.72%), indicating that dynamically regulating the transition between exploration and exploitation is essential for maintaining an effective search. Without this adaptive control, the optimizer loses its ability to balance global exploration and local refinement, leading to slower convergence and lower solution quality.

The spiral exploitation strategy and the proposed dimension-wise adaptive step-size mechanism (DASS) also make substantial contributions, with average performance losses of 11.56% and 11.70%, respectively. The spiral search improves the algorithm’s ability to refine promising solutions during the final stages of optimization, while DASS adjusts the search intensity independently for each decision variable according to its convergence state. This adaptive behavior allows different dimensions to evolve at different rates, resulting in a more stable and efficient convergence process, particularly for problems with complex search landscapes.

Although the Sobol sequence initialization and the Lévy-flight exploration strategy have smaller individual contributions, both remain important parts of the proposed framework. Sobol initialization provides a more uniformly distributed initial population, improving search diversity from the beginning of the optimization. Meanwhile, the Lévy-flight strategy strengthens global exploration by introducing occasional long-distance moves that help the algorithm escape local optima and explore previously unvisited regions of the search space. Removing either mechanism consistently degrades the overall performance, highlighting their supporting role within the complete algorithm.

One interesting observation can be seen for F10, where the NoDASS variant achieved a slightly better result than the complete DASO algorithm. However, this improvement was limited to a single benchmark function and was not reflected in the overall results. Across the remaining representative functions, DASO consistently outperformed the NoDASS variant, indicating that the proposed dimension-wise adaptive step-size mechanism provides a net performance benefit across different optimization landscapes. Such variations are expected in metaheuristic optimization, as individual search operators may perform differently depending on the characteristics of a particular problem.

The convergence curves presented in Fig. 12 further reinforce these findings. The complete DASO algorithm converges more rapidly during the early stages while continuing to improve steadily throughout the optimization process. In comparison, most ablated variants either converge more slowly or stagnate at higher objective values, indicating a reduced ability to balance exploration and exploitation effectively. The effect is particularly noticeable when the adaptive threshold is removed, resulting in slower convergence, and when the spiral search is excluded, reducing the algorithm’s ability to refine promising solutions during the final iterations. Likewise, removing the adaptive step-size mechanism produces a less stable convergence pattern, confirming its role in maintaining efficient search behavior as the population approaches convergence.

Overall, the ablation study confirms that the performance gains achieved by DASO are the result of the combined interaction of all proposed enhancement strategies rather than any single modification. Each component addresses a different aspect of the optimization process, including population initialization, exploration–exploitation control, global search, local refinement, and adaptive convergence. Their integration enables DASO to maintain a balanced search process, improve convergence stability, and achieve consistently superior performance across benchmark functions with different characteristics.

**Table 9:** Ablation Study.

F(x)		SO	DASO	NoSobol	NoThreshold		NoLevy	NoSpiral	NoDASS
F1	Mean	6.802E + 08	**1.577E + 06**	3.363E + 07		2.753E + 08	1.820E + 07	3.036E + 08	3.056E + 08
	Std	3.273E + 08	3.827E + 07	6.293E + 07		3.739E + 08	**2.271E + 07**	2.232E + 08	2.760E + 08
	Rank	7.00	1.00	3.00		4.00	2.00	5.00	6.00
	Loss (%)	4.30E + 04	0.00	2.03E + 03		1.74E + 04	1.05E + 03	1.92E + 04	1.93E + 04
F4	Mean	8.521E + 02	**6.110E + 02**	6.261E + 02		8.053E + 02	6.459E + 02	7.498E + 02	7.562E + 02
	Std	8.540E + 01	**5.721E + 01**	7.172E + 01		1.193E + 02	8.152E + 01	8.387E + 01	6.913E + 01
	Rank	7.00	1.00	2.00		6.00	3.00	4.00	5.00
	Loss (%)	39.46	0.00	2.47		31.80	5.71	22.72	23.76
F5	Mean	7.224E + 02	**6.660E + 02**	7.203E + 02		6.939E + 02	7.215E + 02	6.918E + 02	7.002E + 02
	Std	3.858E + 01	2.506E + 01	3.251E + 01		2.574E + 01	3.602E + 01	**2.444E + 01**	2.612E + 01
	Rank	7.00	1.00	5.00		3.00	6.00	2.00	4.00
	Loss (%)	8.47	0.00	8.15		4.19	8.33	3.87	5.14
F8	Mean	1.010E + 03	**9.724E + 02**	1.011E + 03		1.003E + 03	1.014E + 03	1.001E + 03	1.007E + 03
	Std	3.582E + 01	3.356E + 01	**2.698E + 01**		3.003E + 01	3.532E + 01	3.017E + 01	3.113E + 01
	Rank	5.00	1.00	6.00		3.00	7.00	2.00	4.00
	Loss (%)	3.97	0.00	3.97		3.15	4.28	2.94	3.56
F9	Mean	6.650E + 03	**2.833E + 03**	4.089E + 03		5.404E + 03	3.880E + 03	3.588E + 03	3.793E + 03
	Std	2.604E + 03	1.497E + 03	1.616E + 03		2.379E + 03	1.360E + 03	**9.045E + 02**	1.830E + 03
	Rank	7.00	1.00	5.00		6.00	4.00	2.00	3.00
	Loss (%)	134.73	0.00	44.33		90.75	36.96	26.65	33.89
F10	Mean	9.720E + 03	9.277E + 03	9.340E + 03		1.155E + 04	9.281E + 03	1.081E + 04	**9.130E + 03**
	Std	3.022E + 03	1.980E + 03	2.015E + 03		1.752E + 03	2.137E + 03	2.265E + 03	**1.746E + 03**
	Rank	5.00	2.00	4.00		7.00	3.00	6.00	1.00
	Loss (%)	4.76	0.00	0.68		24.46	0.04	16.55	−1.60
F17	Mean	3.224E + 03	**2.985E + 03**	3.095E + 03		3.096E + 03	3.189E + 03	3.033E + 03	3.085E + 03
	Std	3.163E + 02	2.923E + 02	2.987E + 02		3.624E + 02	**2.891E + 02**	3.390E + 02	3.794E + 02
	Rank	7.00	1.00	4.00		5.00	6.00	2.00	3.00
	Loss (%)	8.01	0.00	3.69		3.72	6.83	1.61	3.35
F28	Mean	4.317E + 03	**3.424E + 03**	3.483E + 03		3.938E + 03	3.485E + 03	3.649E + 03	3.897E + 03
	Std	3.882E + 02	**7.671E + 01**	2.576E + 02		3.578E + 02	2.171E + 02	1.263E + 02	2.467E + 02
	Rank	7.00	1.00	2.00		6.00	3.00	4.00	5.00
	Loss (%)	26.06	0.00	1.70		14.98	1.75	6.56	13.77
Avg Performance Loss (%)		32.21	0.00	9.28		24.72	9.13	11.56	11.70
Contribution		-	-	Moderate		Very High	Moderate	High	High

### Discussion

The benchmark experiments, together with the exploration–exploitation analysis and the ablation study, provide a clear picture of the behavior and effectiveness of the proposed DASO algorithm. The benchmark results show that DASO performs competitively and, in most cases, better than the original Snake Optimizer and the selected comparison algorithms. It also converges faster in many cases while remaining stable across benchmark functions with different characteristics. These results indicate that the proposed modifications improve search efficiency without reducing robustness.

The behavioral analysis explains why these improvements occur. The diversity results show that DASO starts with a well-distributed population and maintains diversity during the early and middle stages of the search before gradually focusing on promising regions. The exploration–exploitation analysis shows a similar pattern: exploration dominates at the beginning, then gradually shifts toward exploitation as the algorithm converges. This allows DASO to search broadly first and then refine solutions more effectively, which helps reduce premature convergence. The dimension-wise adaptive step-size also shows that different variables are updated according to their own convergence state, rather than using a single global step size. Together, these results confirm that the proposed adaptive mechanisms work as intended.

The ablation study further supports this conclusion. Removing any one of the proposed components reduces performance, which shows that each enhancement contributes to the final result. The adaptive threshold has the largest impact, indicating that it plays a central role in balancing exploration and exploitation. The spiral exploitation strategy and the dimension-wise adaptive step-size also contribute significantly by improving local refinement and convergence stability. Sobol initialization and Lévy-flight exploration mainly strengthen population diversity and global search. Although their individual effects differ, the results show that no single component alone explains the overall improvement.

Each strategy addresses a different part of the optimization process. Sobol initialization improves the quality of the initial population, the adaptive threshold controls the search behavior during optimization, Lévy-flight exploration helps escape local optima, the spiral search improves solution refinement, and the dimension-wise adaptive step-size adjusts the search intensity for each variable. By combining these mechanisms, DASO maintains diversity early in the search and increases accuracy as convergence progresses.

Overall, the benchmark results, behavioral analysis, and ablation study consistently show that DASO is effective because of the complementary interaction of its components. The algorithm achieves a better balance between exploration and exploitation, which leads to improved convergence accuracy, robustness, and optimization performance. These findings support the effectiveness of DASO on the benchmark functions considered in this study and provide a strong basis for its evaluation on the photovoltaic parameter estimation problem in the following section.

Despite the encouraging results, several limitations of the present study should be acknowledged. First, the scalability of DASO was evaluated on benchmark problems with dimensions up to 50; therefore, its performance on very large-scale optimization problems remains to be investigated. Second, the proposed adaptive parameters were selected according to preliminary experiments and showed stable performance throughout this work, however, a comprehensive parameter sensitivity analysis was not performed. Additionally, the suggested technique was verified using benchmark functions and photovoltaic parameter estimation, while the application of the proposed algorithm to other engineering optimization problems has not been investigated yet. Finally, while a computational complexity analysis has been provided, a detailed experimental comparison of runtime performance was beyond the scope of this work. Addressing these limitations will further strengthen the understanding of DASO and represent an important direction for future work.

## Experimental results and analysis on parameter identification of PV cells models

The objective of solar photovoltaic model parameter identification is to reduce the difference between the current value predicted by the model and the actual current value by establishing the optimal values of the parameters through the analysis of measured output voltage and current data. Because of this, the objective function of the system operation is the root mean square error (RMSE) between the simulated and actual currents, which transforms the issue of identifying the parameters into a search for the ideal value within a set range. Equation (45) is used to determine the objective function RMSE^42^:

45 $$\:\begin{array}{cccc}&\:\mathrm{R}\mathrm{M}\mathrm{S}\mathrm{E}=\sqrt{\frac{1}{M}\sum\:_{i=1}^{M}f{\left({V}_{L},{I}_{L},\mathbf{x}\right)}^{2}}&\:&\:\end{array}$$

Where * M* is the number of iterations, $\:f({V}_{L},{I}_{L},\mathbf{x})$ is the objective function of the PV cell model, and $\:\mathbf{x}$ is the PV cell parameter vector to be estimated. The performance of DASO in parameter identification is tested against the original the original SO^16^, GWO^12^, SCA^26^, HHO^27^, MFO^28^, and WOA^13^. The current-voltage measurements utilized are obtained from a 57 mm French R.T.C commercial solar cell at an irradiation of 1000 W/m² and a cell operating temperature of 33 °C^66^. The computational model employed in this study is an AMD Ryzen 5 3600 6-Core Processor at 3.6 GHz with 16 GB of RAM, and the simulation platform utilized is MATLAB 2023b. The size of the population is fixed at 30 and the number of iterations for all methods is fixed at 500. To ensure the accuracy of the identification findings, each algorithm is run separately thirty times. Equation (46) and Eq. (47) describe the absolute power errors and absolute current errors, respectively:

46 $$\:absolute\:power\:errors=\:\sum\:_{i=1}^{N}\left|{P}_{cal}-{P}_{mes}\:\right|$$

47 $$\:absolute\:current\:errors=\:\sum\:_{i=1}^{N}\left|{I}_{cal}-{I}_{mes}\:\right|$$

In case of the SDM, the objective function is described using Eq. (48):

48 $$\:f\left({V}_{L},{I}_{L},\mathbf{x}\right)={I}_{ph}-{I}_{sd}\left\{\mathrm{e}\mathrm{x}\mathrm{p}\left[\frac{q\left({V}_{L}+{R}_{s}{I}_{L}\right)}{nkT}\right]-1\right\}-\frac{{V}_{L}+{R}_{s}{I}_{L}}{{R}_{sh}}-{I}_{L}$$

**Fig. 13:**
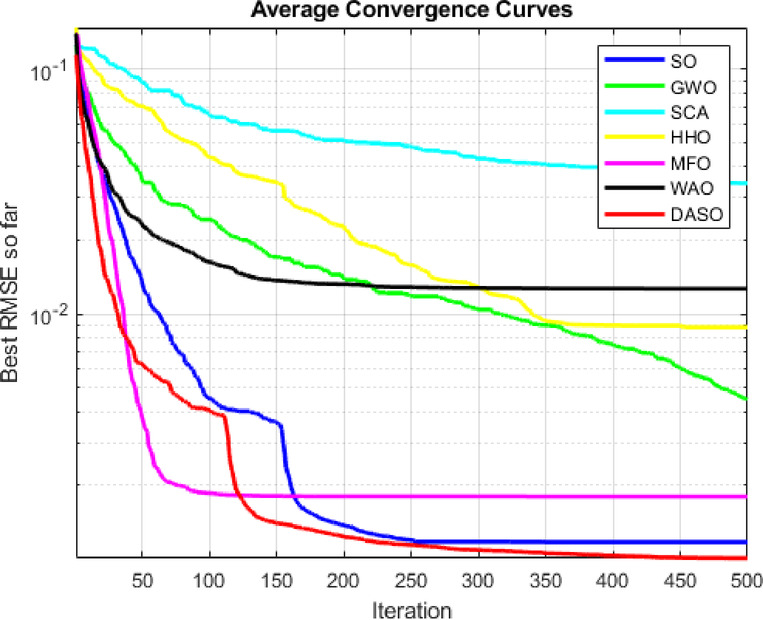
Convergence-Curve with a single-diode model for DASO vs other algorithms.

**Fig. 14:**
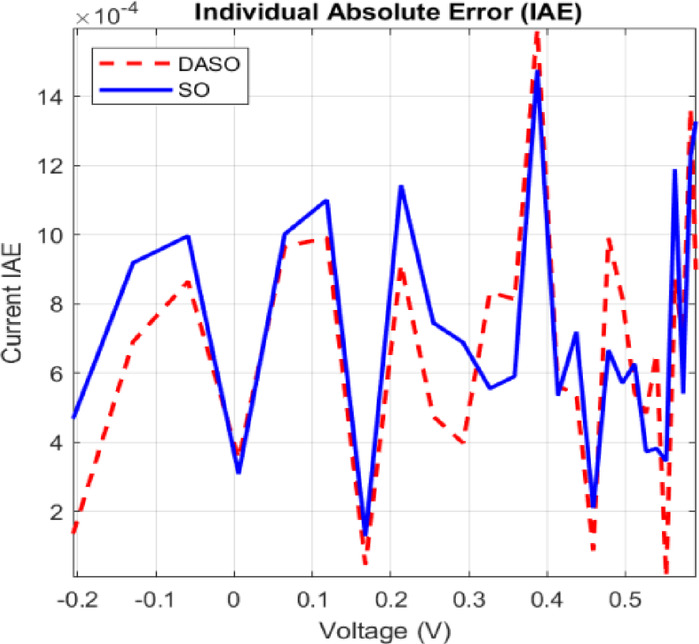
Individual absolute error with a single-diode model for DASO vs SO.

**Fig. 15:**
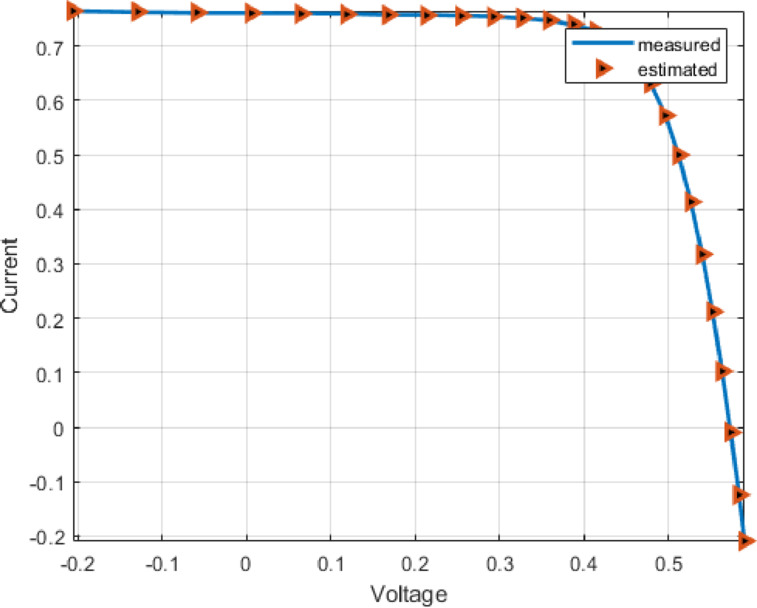
The Single-diode model I-V plot between the measured and estimated values.

**Fig. 16:**
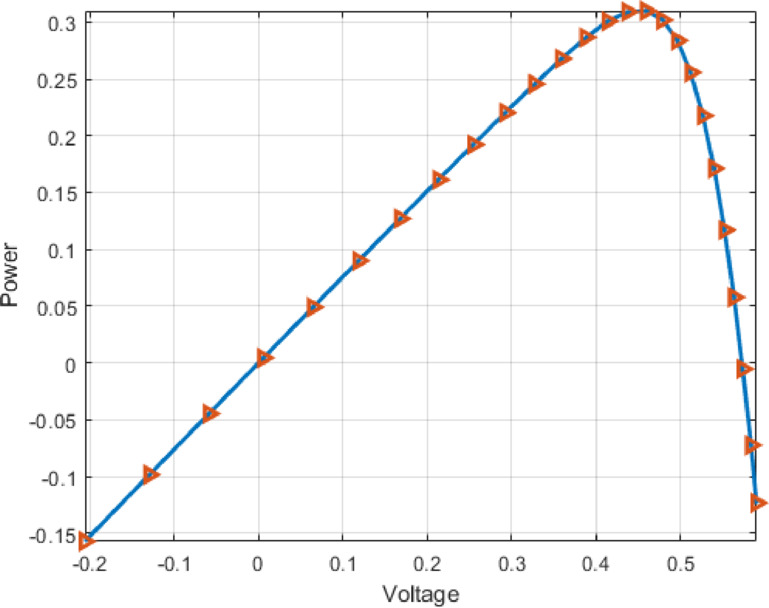
Measurements of a single diode and estimates of the consequent potential-voltage (P-V) curve.

The identified parameter vectors for the PV cell for each optimization algorithm and their RMSE values are presented in Table 10. Then, we apply the proposed DASO and the standard SO to the issue of determining solar photovoltaic model parameters. The observed and simulated currents and power, along with the absolute power errors and the absolute current error values of the proposed DASO and the original SO algorithms are presented in Table 11. These results indicate that the performance of the DASO algorithm is superior to that of the standard SO. When compared to SO, the absolute errors in power and current are lower. The results show that the obtained current values are closely in line with the experimental data throughout the whole measured voltage range. The comparison between the individual absolute current errors of DASO and SO reveals that, although DASO has larger errors than SO at some voltages, it has an overall smaller error in parameter identification. From the convergence curves shown in Fig. 13, DASO exhibits faster convergence speeds and higher solution precision. The absolute current error values computed by DASO and SO are compared in Fig. 14. The I-V and P-V characteristics of a photovoltaic cell, utilizing both actual and computed parameters by DASO, are illustrated in Fig. 15 and Fig. 16, respectively.

The objective function of the DDM is described using Eq. (49):

49 $$\:f\left({V}_{L},{I}_{L},\mathrm{x}\right)={I}_{ph}-{I}_{sd1}\left\{\mathrm{exp}\left[\frac{q\left({V}_{L}+{R}_{s}{I}_{L}\right)}{{n}_{1}kT}\right]-1\right\}-{I}_{sd2}\left\{\mathrm{exp}\left[\frac{q\left({V}_{L}+{R}_{s}{I}_{L}\right)}{{n}_{2}kT}\right]-1\right\}-\frac{{V}_{L}+{R}_{s}{I}_{L}}{{R}_{sh}}-{I}_{L}$$

**Fig. 17:**
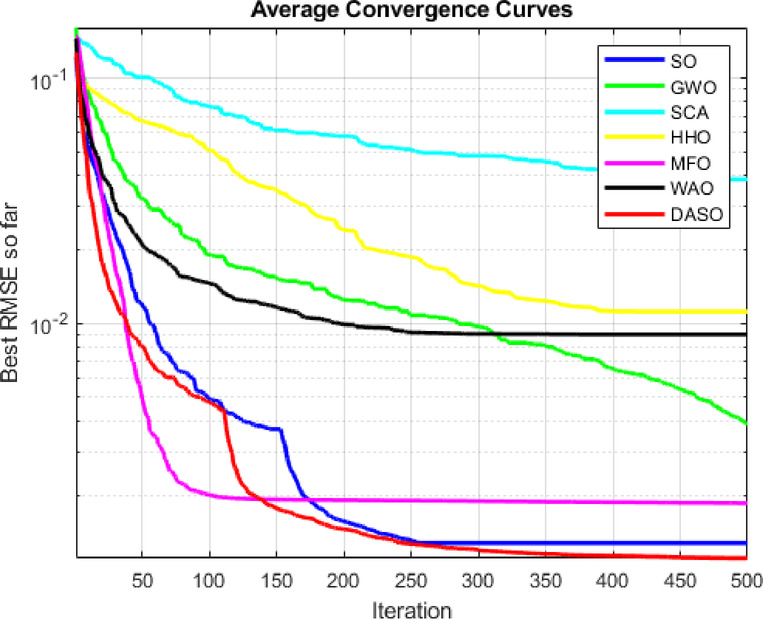
Convergence-Curve with a double-diode model for DASO vs other algorithms.

**Fig. 18:**
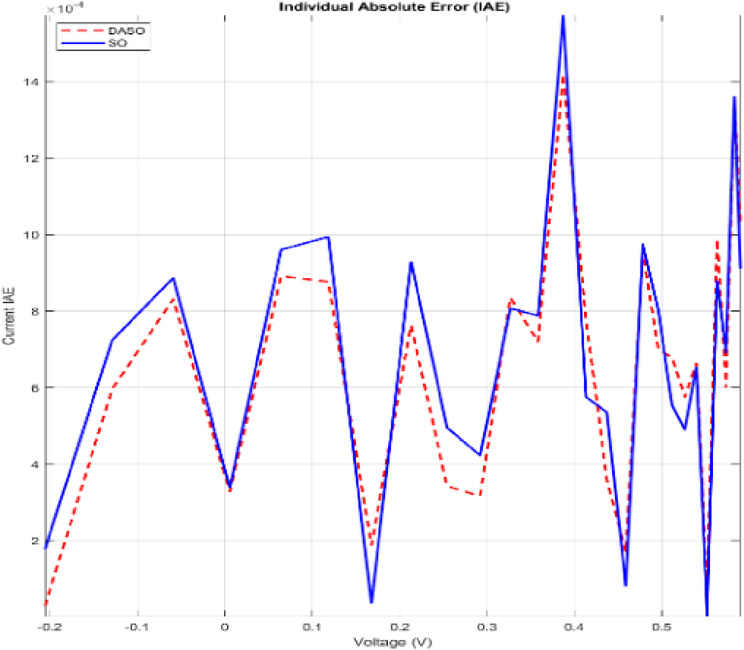
Individual absolute error with a double-diode model for DASO vs SO.

**Fig. 19:**
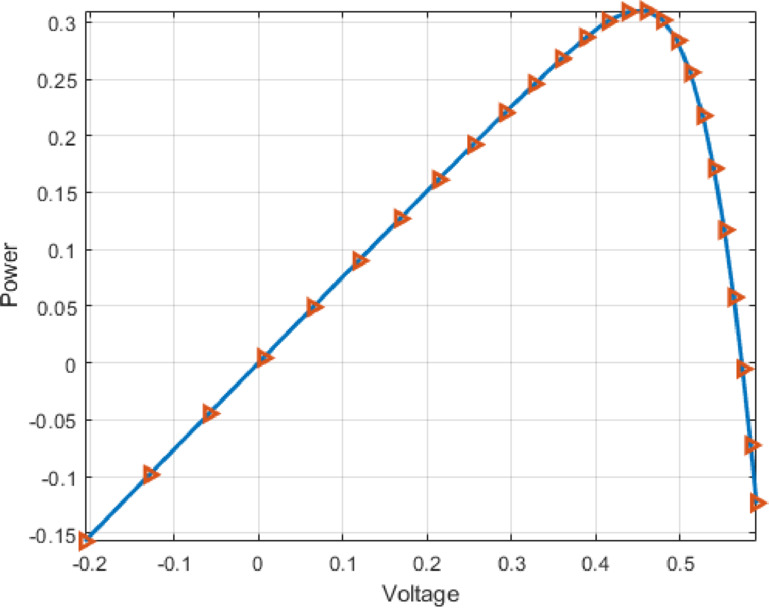
Double-diode measurements and estimates of the resulting potential-voltage (P-V) curve.

**Fig. 20:**
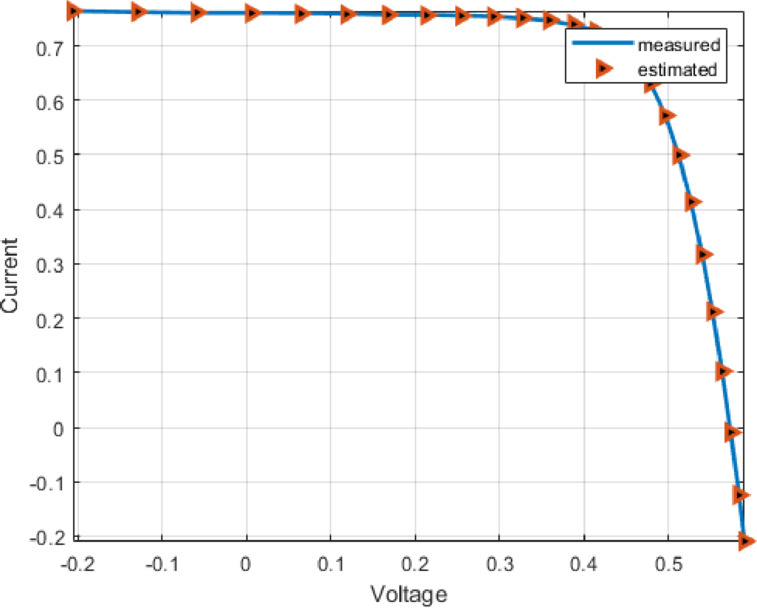
The Double-diode model I-V plot between the measured and estimated values.

The identified parameter vectors for the PV cell for each optimization algorithm and their RMSE values are presented in Table 12. The observed and simulated currents and power, along with the absolute power errors and the absolute current error values of the proposed DASO and the original SO algorithms are presented in Table 13. These results indicate that the performance of the DASO algorithm is superior to that of the standard SO. When compared to SO, the absolute errors in power and current are lower. The results show that the obtained current values are closely in line with the experimental data throughout the whole measured voltage range. From the convergence curves shown in Fig. 17, DASO exhibits faster convergence speeds and higher solution precision. The absolute current error values computed by DASO and SO are compared in Fig. 18. Figure 19 and Fig. 20 presents the I-V and P-V characteristics of a PV cell, utilizing both actual and computed parameters by DASO.

In conclusion, DASO obtained the lowest RMSE value signifying a superior alignment between the algorithm-simulated current values and the actual measured values. Accordingly, DASO has superior parameters identification accuracy for both SDM and DDM, thanks to the introduction of DASS, dynamic adaptive parameters and hybrid strategies that enhanced the precision of SO.

**Table 10:** SDM estimated parameters with DASO vs. Other Algorithms.

	$\:{\boldsymbol{I}}_{\boldsymbol{p}\boldsymbol{h}}$	$\:{\boldsymbol{I}}_{\boldsymbol{s}\boldsymbol{d}}$	$\:{\boldsymbol{R}}_{\boldsymbol{s}\boldsymbol{h}\:}$	$\:{\boldsymbol{R}}_{\boldsymbol{s}\:}$	$\:\boldsymbol{n}$	RMSE
SO	0.760879873	0.268353399	49.40823197	0.03719871	1.462739507	0.00080724
DASO	**0.760780269**	**0.310266366**	**52.9963875**	**0.036554884**	**1.477130926**	**0.000773047**
GWO	0.76024916	0.434574272	68.31154899	0.03511866	1.511706302	0.00104463
SCA	0.760873539	0.906881943	100	0.02985862	1.592389504	0.00820312
HHO	0.760022255	0.357245112	70.92997646	0.035868282	1.491151276	0.00105314
MFO	0.760711498	0.385166778	58.1333738	0.03560266	1.499132492	0.000843643
WOA	0.761279321	0.35210645	46.72016014	0.035729568	1.490158096	0.000959788

**Table 11:** Actual and simulated current and power utilizing a single-diode model for DASO vs. SO.

$\:{\boldsymbol{V}}_{\boldsymbol{t}\boldsymbol{m}}$	$\:{\boldsymbol{I}}_{\boldsymbol{t}\boldsymbol{m}}$	SO Current Estimated	SO Absolute current error	DASO Current Estimated	DASO Absolute current error	SO Power	SO Absolute power error	DASO Power	DASO Absolute power error
−0.2057	0.764	0.764132193	0.00013219	0.763955841	4.41591E-05	−0.157181992	0.04851801	−0.157145716	0.04855428
−0.1291	0.762	0.762681361	0.00068136	0.762551732	0.000551732	−0.098462164	0.03063784	−0.098445429	0.03065457
−0.0588	0.7605	0.761349756	0.00084976	0.761263003	0.000763003	−0.044767366	0.01403263	−0.044762265	0.01403774
0.0057	0.76	0.760127536	0.00012754	0.760080103	8.0103E-05	0.004332727	0.00136727	0.004332457	0.00136754
0.0646	0.76	0.759009416	0.00099058	0.758997823	0.001002177	0.049032008	0.01556799	0.049031259	0.01556874
0.1185	0.759	0.757978769	0.00102123	0.757999764	0.001000236	0.089820484	0.02867952	0.089822972	0.02867703
0.1678	0.757	0.757011814	1.1814E-05	0.757062033	6.20328E-05	0.127026582	0.04077342	0.127035009	0.04076499
0.2132	0.757	0.756049818	0.00095018	0.756125505	0.000874495	0.161189821	0.05201018	0.161205958	0.05199404
0.2545	0.7555	0.754987647	0.00051235	0.755083383	0.000416617	0.192144356	0.06235564	0.192168721	0.06233128
0.2924	0.754	0.753565478	0.00043452	0.753673591	0.000326409	0.220342546	0.07205745	0.220374158	0.07202584
0.3269	0.7505	0.751302179	0.00080218	0.751411374	0.000911374	0.245600682	0.08129932	0.245636378	0.08126362
0.3585	0.7465	0.747292604	0.0007926	0.747387653	0.000887653	0.267904399	0.0905956	0.267938474	0.09056153
0.3873	0.7385	0.74009047	0.00159047	0.740153599	0.001653599	0.286637039	0.10066296	0.286661489	0.10063851
0.4137	0.728	0.727455631	0.00054437	0.727470414	0.000529586	0.300948395	0.11275161	0.30095451	0.11274549
0.4373	0.7065	0.707078928	0.00057893	0.70703881	0.00053881	0.309205615	0.12809438	0.309188072	0.12811193
0.459	0.6755	0.675470376	2.9624E-05	0.675382314	0.000117686	0.310040903	0.1489591	0.310000482	0.14899952
0.4784	0.632	0.631071369	0.00092863	0.630959575	0.001040425	0.301904543	0.17649546	0.301851061	0.17654894
0.496	0.573	0.572234532	0.00076547	0.572131415	0.000868585	0.283828328	0.21217167	0.283777182	0.21222282
0.5119	0.499	0.499571949	0.00057195	0.499506668	0.000506668	0.255730881	0.25616912	0.255697463	0.25620254
0.5265	0.413	0.413484735	0.00048473	0.413473732	0.000473732	0.217699713	0.30880029	0.21769392	0.30880608
0.5398	0.3165	0.317131524	0.00063152	0.317173233	0.000673233	0.171187597	0.3686124	0.171210111	0.36858989
0.5521	0.212	0.211967546	3.2454E-05	0.212045158	4.51576E-05	0.117027282	0.43507272	0.117070131	0.43502987
0.5633	0.1035	0.102584302	0.0009157	0.102669901	0.000830099	0.057785738	0.50551426	0.057833955	0.50546604
0.5736	−0.01	−0.009336485	0.00066351	−0.009275023	0.000724977	−0.005355408	0.57895541	−0.005320153	0.57892015
0.5833	−0.123	−0.124367239	0.00136724	−0.124363727	0.001363727	−0.07254341	0.65584341	−0.072541362	0.65584136
0.59	−0.21	−0.209073196	0.0009268	−0.209132703	0.000867297	−0.123353185	0.71335319	−0.123388295	0.71338829
Mean Error			0.00066684		**0.000659753**		0.20151349		**0.201512025**

**Table 12:** DDM estimated parameters with DASO vs. Other Algorithms.

	$\:{\boldsymbol{I}}_{\boldsymbol{p}\boldsymbol{h}}$	$\:{\boldsymbol{I}}_{\boldsymbol{s}\boldsymbol{d}1}$	$\:{\boldsymbol{I}}_{\boldsymbol{s}\boldsymbol{d}2}$	$\:{\boldsymbol{R}}_{\boldsymbol{s}\boldsymbol{h}\:}$	$\:{\boldsymbol{R}}_{\boldsymbol{s}\:}$	$\:{\boldsymbol{n}}_{1}$	$\:{\boldsymbol{n}}_{2}$	RMSE
SO	0.760798797	0.3065121	0.003893301	52.60094338	0.036585031	1.4760242	1.772556864	0.000775834
DASO	**0.760784634**	**0.1531601**	**1**	**55.65025677**	**0.037258243**	**1.4194537**	**1.927502459**	**0.000744535**
GWO	0.759569537	0.1655832	0.282541424	57.54198818	0.03701181	1.4270805	1.728891826	0.00121285
SCA	0.766116323	0.0228893	0	12.25771588	0.041738478	1.2582161	1.381879805	0.0080164
HHO	0.760719718	0.2236591	0.152530014	53.02147431	0.034892234	1.4999695	1.492241044	0.00111651
MFO	0.760784865	0	0.313366237	53.07861599	0.036509655	1.9999971	1.478131908	0.000780038
WOA	0.759546404	0.9893797	0.078708725	76.56671888	0.037302011	1.7616496	1.381227295	0.00128164

## Conclusion and future work

Meta-heuristic optimization approaches are increasingly being employed to address a range of engineering challenges. In addition to existing algorithms, new algorithms with improved capabilities are continually introduced to rectify the weaknesses of the currently utilized methods. This study proposed a novel algorithm called DASO, which is a modification of the recently developed Snake Optimizer (SO) algorithm. DASO combines Sobol sequence initialization, an adaptive threshold mechanism, Lévy-flight exploration, spiral exploitation, and a dimension-wise adaptive step-size strategy within a unified optimization framework. These mechanisms were combined to enhance population diversity, improve the balance between exploration and exploitation, reduce premature convergence, and strengthen the search performance, particularly for challenging optimization problems on the benchmark optimization problems and photovoltaic parameter estimation considered in this study.

**Table 13:** Actual and simulated current and power utilizing a single-diode model for DASO vs SO.

$V_{{tm}}$	$I_{{tm}}$	**SO Current Estimated**	**SO** **Absolute current error**	**DASO Current Estimated**	**DASO Absolute current error**	**SO Power**	**SO** **Absolute power error**	**DASO Power**	**DASO** **Absolute power error**
−0.2057	0.764	0.764178179	0.00017818	0.763970571	2.94294E-05	−0.157148731	0.048551269	−0.157178191	0.048521809
−0.1291	0.762	0.762722923	0.00072292	0.762594921	0.000594921	−0.098451247	0.030648753	−0.098460514	0.030639486
−0.0588	0.7605	0.761387257	0.00088726	0.761332051	0.000832051	−0.044766546	0.014033454	−0.044766922	0.014033078
0.0057	0.76	0.760161304	0.0001613	0.760172089	0.000172089	0.004333014	0.001366986	0.004332711	0.001367289
0.0646	0.76	0.759039751	0.00096025	0.759108695	0.000891305	0.049038951	0.015561049	0.049032112	0.015567888
0.1185	0.759	0.758005897	0.0009941	0.758123221	0.000876779	0.089838969	0.028661031	0.089821141	0.028678859
0.1678	0.757	0.757035834	3.5834E-05	0.757187644	0.000187644	0.127058851	0.040741149	0.127028093	0.040771907
0.2132	0.757	0.756070574	0.00092943	0.756236725	0.000763275	0.161234695	0.051965305	0.161192345	0.052007655
0.2545	0.7555	0.755004614	0.00049539	0.755157304	0.000342696	0.192195845	0.062304155	0.192147831	0.062352169
0.2924	0.754	0.753577475	0.00042252	0.75368339	0.00031661	0.220389439	0.072010561	0.220346599	0.072053401
0.3269	0.7505	0.751307287	0.00080729	0.751335085	0.000835085	0.245627752	0.081272248	0.245604478	0.081295522
0.3585	0.7465	0.747288162	0.00078816	0.747219778	0.000719778	0.267896441	0.090603559	0.267906543	0.090593457
0.3873	0.7385	0.740073735	0.00157374	0.739918673	0.001418673	0.286586085	0.100713915	0.286635686	0.100664314
0.4137	0.728	0.727424917	0.00057508	0.727226267	0.000773733	0.300860368	0.112839632	0.300941677	0.112758323
0.4373	0.7065	0.707035743	0.00053574	0.706860332	0.000360332	0.309102747	0.128197253	0.309192716	0.128107284
0.459	0.6755	0.675419979	8.0021E-05	0.675330171	0.000169829	0.309952665	0.149047335	0.310022702	0.148977298
0.4784	0.632	0.631022807	0.00097719	0.631041855	0.000958145	0.301853074	0.176546926	0.301884252	0.176515748
0.496	0.573	0.572197736	0.00080226	0.572299919	0.000700081	0.283817384	0.212182616	0.283810479	0.212189521
0.5119	0.499	0.49955429	0.00055429	0.499679476	0.000679476	0.255745608	0.256154392	0.255719761	0.256180239
0.5265	0.413	0.413488912	0.00048891	0.413574684	0.000574684	0.217717383	0.308782617	0.217697954	0.308802046
0.5398	0.3165	0.317154496	0.0006545	0.317163871	0.000663871	0.171189882	0.368610118	0.171195206	0.368604794
0.5521	0.212	0.212001975	1.9753E-06	0.211934718	6.52819E-05	0.117008568	0.435091432	0.117041905	0.435058095
0.5633	0.1035	0.102620028	0.00087997	0.102512037	0.000987963	0.057755666	0.505544334	0.057803117	0.505496883
0.5736	−0.01	−0.009310304	0.0006897	−0.009400147	0.000599853	−0.005375232	0.578975232	−0.005340389	0.578940389
0.5833	−0.123	−0.124361532	0.00136153	−0.124359944	0.001359944	−0.072522449	0.655822449	−0.072536322	0.655836322
0.59	−0.21	−0.209088928	0.00091107	−0.208970547	0.001029453	−0.123279965	0.713279965	−0.123355423	0.713355423
Mean Error			0.00067187		**0.000650115**		0.201519528		**0.2015142**

Our suggested method DASO is evaluated not just against the original SO but also against contemporary algorithms, namely GWO, SCA, HHO, MFO, and WOA. We have tested its performance using different benchmark functions with varying difficulties: CEC2017 with three different dimensions, and CEC2020. Compared to the mentioned algorithms, the enhanced modification DASO exhibited a superior performance in the majority of benchmark functions, which is clearly demonstrated by several convergence curves. The experimental results were statistically validated using non-parametric statistical tests. The overall performance differences among the algorithms studied utilizing all of the benchmarks were assessed using the Friedman test. The DASO rank is compared to other algorithms. Friedman tests revealed that the suggested algorithm performs better than other algorithms considered in this study. Additionally, pairwise comparisons were performed with the Wilcoxon signed-rank test at a level of significance of α = 0.05. According to the findings of the Wilcoxon test, the suggested algorithm substantially surpasses the reference algorithms for the great majority of the benchmark functions. The statistical analyses, based on the Friedman ranking test and the Wilcoxon signed-rank test, confirmed that the observed improvements were statistically significant. In addition, the convergence characteristics demonstrated that DASO was able to achieve high-quality solutions while maintaining stable convergence throughout the optimization process.

To better understand the behavior of the proposed algorithm, an exploration–exploitation analysis and an ablation study were also carried out. The behavioral analysis showed that DASO preserves population diversity during the early stages of the search and gradually shifts its focus toward exploitation as the optimization progresses. This adaptive transition enables the algorithm to avoid premature convergence while maintaining efficient local search during the final iterations. The ablation study further confirmed that each enhancement contributes to the overall performance of DASO. Although the contribution of each individual component varies across different optimization problems, the complete framework consistently achieved the best overall performance, highlighting the effectiveness of integrating these complementary search strategies.

Finally, DASO is employed for estimating the parameters of both single-diode and double-diode PV cell models. According to the results in terms of the absolute error of the current and the power for different operating conditions, DASO achieved competitive performance compared with the reference algorithms considered in this study. Additionally, the accuracy of the identified parameters for the two models was compared using the RMSE value. DASO managed to achieve the lowest value of RMSE among the compared algorithms for both PV models, which indicated a superior accuracy in comparison with the competing algorithms. Also, the corresponding convergence curves for both models demonstrated faster and more stable convergence behavior of DASO. These results indicate that the proposed algorithm is effective not only on the benchmark optimization problems considered in this study but also on the investigated photovoltaic parameter estimation problem, highlighting its potential for practical nonlinear engineering optimization applications.

Despite these encouraging results, the present study has several limitations. The benchmark evaluation follows the standard CEC2017 experimental protocol using problem dimensions of 10, 30, and 50, and therefore the scalability of DASO for very large-scale optimization problems remains to be investigated. In addition, the practical validation is limited to photovoltaic parameter estimation, and evaluating the proposed framework on a broader range of engineering optimization problems would provide a more comprehensive assessment of its generalization capability. Although the computational complexity of DASO has been analyzed theoretically, a detailed experimental comparison of computational efficiency under identical implementation conditions would provide further insight into the practical cost of the proposed enhancements. Furthermore, while the proposed adaptive mechanisms are supported by extensive experimental validation, a rigorous theoretical convergence analysis has not been established. Another limitation of the present study is that direct comparisons with additional recent state-of-the-art optimization algorithms were not included. Consequently, the conclusions drawn from this work are limited to the benchmark problems and optimization algorithms considered in the experimental evaluation. Finally, comprehensive parameter sensitivity analysis was not conducted and should be considered in future investigations.

Future work will focus on extending DASO to more challenging optimization scenarios, including large-scale, constrained, multi-objective, and dynamic optimization problems. Another important direction is the application of DASO to a wider range of real-world engineering problems, particularly in renewable energy systems, engineering design, parameter estimation, and optimal control to further assess its generalization capability. In addition, future studies will investigate the sensitivity of the proposed control parameters, perform comprehensive runtime benchmarking, and explore more advanced adaptive learning mechanisms that further improve the balance between exploration and exploitation while preserving the robustness of the optimization process. Further work will also include formal theoretical convergence analysis, the incorporation of complementary statistical measures such as effect-size analysis, broader comparisons with recent state-of-the-art optimization algorithms, and more comprehensive evaluations on photovoltaic parameter estimation problems under varying irradiance, temperature, and noisy operating conditions to further assess the competitiveness and robustness of DASO.

## Data Availability

The datasets used and/or analyzed during the current study available from the corresponding author on reasonable request.
